# Recent Advances in Quinoline Synthesis: Sustainable Catalytic Strategies and Emerging Methodologies

**DOI:** 10.3390/molecules31122081

**Published:** 2026-06-13

**Authors:** Ignacio M. López-Coca, Shima Ghafouriraz, Silvia Izquierdo, Carlos J. Durán-Valle, Mohammad Qandalee, Alireza Soltani

**Affiliations:** 1 Department of Organic and Inorganic Chemistry, School of Technology, Universidad de Extremadura, 10003 Cáceres, Spain; 2 Research Institute for Sustainable Land Development (INTERRA), Universidad de Extremadura, 10003 Cáceres, Spain; 3 Department of Organic and Inorganic Chemistry, Faculty of Sciences, Universidad de Extremadura, 06006 Badajoz, Spain; carlosdv@unex.es; 4 Research Institute for Water, Climate Change and Sustainability (IACYS), Universidad de Extremadura, 06006 Badajoz, Spain; 5 Department of Basic Sciences, Ga.C., Islamic Azad University, Garmsar 3581631167, Iran; 6 Golestan Rheumatology Research Center, Biomedical Research Institute, Golestan University of Medical Sciences, Gorgan 4934174515, Iran

**Keywords:** quinoline synthesis, green chemistry, heterogeneous catalysis, heterocyclic compounds, Friedländer reaction, Povarov reaction

## Abstract

Quinoline derivatives constitute a privileged class of nitrogen-containing heterocycles with extensive applications in medicinal chemistry, agrochemicals, materials science, and functional organic materials. Owing to their broad biological and industrial relevance, the development of efficient, selective, and sustainable synthetic methodologies for quinoline construction remains an active area of research. This review provides a comprehensive overview of recent advances in quinoline synthesis, with particular emphasis on catalytic strategies aligned with the principles of green and sustainable chemistry. Classical transformations, including the Friedländer, Skraup, and Povarov reactions, are revisited in the context of modern catalytic developments that improve reaction efficiency, substrate scope, selectivity, and environmental compatibility. Special attention is devoted to homogeneous and heterogeneous catalytic systems based on both platinum-group and earth-abundant transition metals, highlighting the growing importance of borrowing-hydrogen and acceptorless dehydrogenative coupling methodologies. Recent progress in nanocatalysis, photocatalysis, multicomponent reactions, ionic-liquid-mediated transformations, and metal-free protocols is also critically discussed. Furthermore, solvent-free processes, microwave-assisted synthesis, and recyclable catalytic systems are examined as practical approaches toward minimizing waste generation and energy consumption. Mechanistic aspects, catalytic design principles, substrate limitations, and sustainability metrics are evaluated throughout the review to provide a critical perspective on current methodologies. Collectively, the advances summarized herein demonstrate the rapid evolution of quinoline synthesis toward more atom-economical, environmentally benign, and operationally efficient processes, while also identifying future opportunities for the development of next-generation catalytic platforms for quinoline-based heterocycle construction.

## 1. Introduction

**Figure 1:**
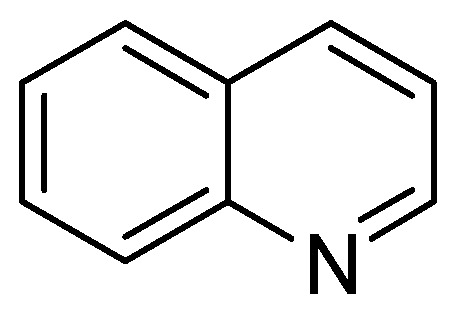
Structure of quinoline.

Heterocyclic chemistry represents one of the most significant domains of organic chemistry, providing access to a vast array of molecules with biological and functional relevance. Heterocyclic frameworks play indispensable roles in biochemistry, materials science, and medicinal chemistry, and are integral to cellular metabolism in living organisms. A large proportion of approved pharmaceuticals and bioactive compounds contain heterocyclic motifs [1]. Among these, quinoline constitutes an important class of fused *N*-heteroaromatic compounds with broad industrial and biomedical relevance. Structurally, quinoline (Figure 1) consists of a benzene ring fused to a pyridine ring at adjacent carbon atoms and is also referred to as benzopyridine or 1-benzazine. It is a hygroscopic liquid with a characteristic odor, sparingly soluble in cold water but readily soluble in hot water and most organic solvents. Although the parent quinoline has limited direct applications, its derivatives are extensively employed in pharmaceuticals, agrochemicals, functional materials, and dyes [2,3,4,5,6]. Consequently, the development of efficient, clean, and sustainable synthetic routes to quinoline scaffolds has long attracted considerable attention [7,8,9,10].

**Figure 2:**
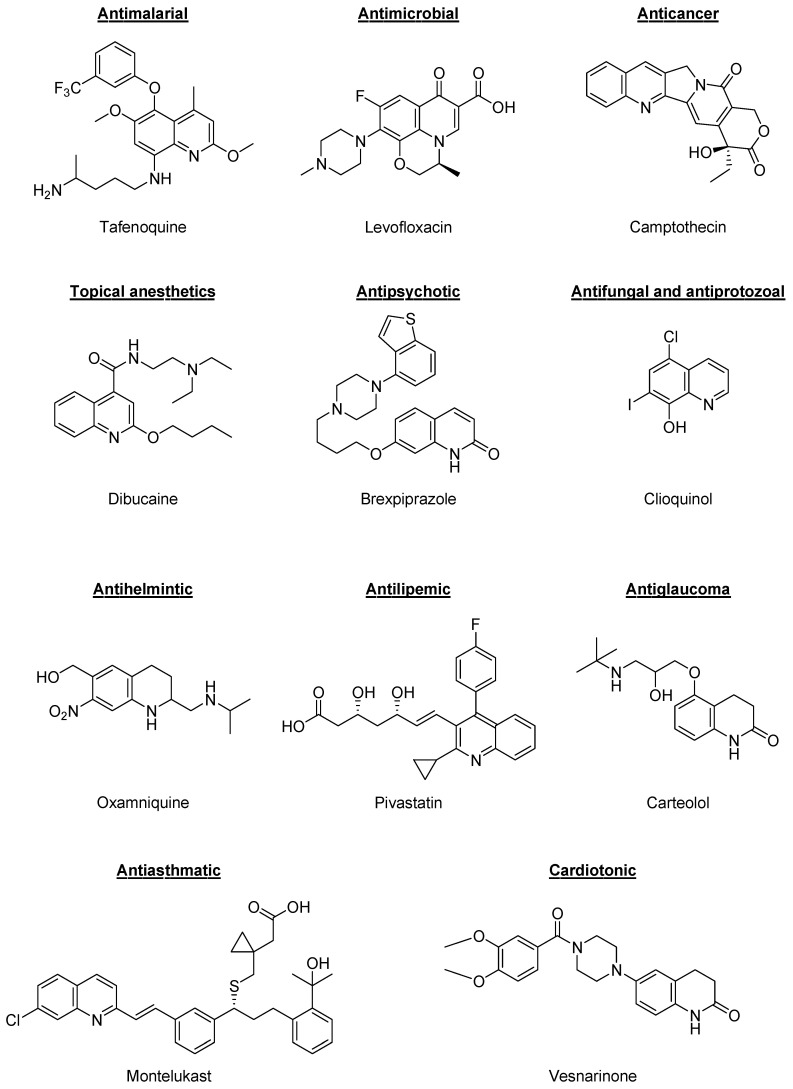
Examples of biologically active quinoline derivatives.

Quinoline derivatives display a wide spectrum of pharmacological activities, including antiparasitic, antibacterial, antiviral, and anticancer activities, among others. Some examples are illustrated in Figure 2 [11]; they highlight the quinoline nucleus as a privileged scaffold in both natural and synthetic bioactive molecules, reinforcing the importance of innovative synthetic strategies [4,10,12,13,14,15,16,17].

Numerous classical methodologies have been developed for quinoline synthesis, among which the Friedländer condensation remains one of the most widely used. This reaction involves the condensation of *o*-aminoaryl carbonyl compounds with carbonyl partners bearing an α-methylene group. Despite its synthetic utility, the traditional Friedländer approach often requires harsh conditions, elevated temperatures, and exhibits limited functional-group tolerance [18,19]. Consequently, substantial research efforts have been directed toward more efficient and sustainable quinoline-forming strategies. In this context, multicomponent reactions (MCRs) have emerged as powerful alternatives, enabling rapid and convergent assembly of quinoline frameworks [20,21].

Multicomponent reactions allow the one-pot formation of complex molecular architectures from three or more starting materials and offer notable advantages over stepwise syntheses, including high atom economy, operational simplicity, and broad functional-group tolerance. These characteristics make MCRs particularly attractive for the sustainable preparation of heterocyclic libraries and bioactive scaffolds [20,22,23,24]. Beyond MCR strategies, several named reactions remain important for quinoline construction, including the Skraup and Friedländer syntheses. Among modern approaches, the imino-Diels–Alder (Povarov) reaction, formally a [4+2] cycloaddition between 2-azadienes and electron-rich alkenes or alkynes, has recently emerged as an efficient route to quinoline and tetrahydroquinoline frameworks [8,25,26].

This review summarizes recent advances in sustainable quinoline synthesis, with emphasis on multicomponent and catalytic methodologies. Particular attention is given to metal-catalyzed, photocatalyzed, metal-free, nanocatalyzed, catalyst-free, and solvent-free processes, as well as heterogeneous catalytic systems and ionic-liquid-based approaches that enable greener access to quinoline derivatives.

## 2. Homogeneous Catalysis by Metals

This section focuses on homogeneous transition-metal-catalyzed approaches, in which the catalyst is present in the same phase as the reactants and operates through molecularly defined active species. In contrast, heterogeneous metal-based systems and nanostructured catalysts, which function in a separate phase, are discussed in a dedicated section below. Photocatalytic processes involving light-driven activation are likewise treated separately to highlight their distinct mechanistic features.

In recent decades, transition- and post-transition-metal catalysis has emerged as a central strategy for the construction of quinoline-based heterocycles, particularly in medicinal and synthetic organic chemistry. The diverse coordination chemistry and tunable reactivity of metal centers enable efficient activation of carbonyl compounds, imines, and unsaturated substrates, thereby facilitating the assembly of structurally complex quinoline frameworks. Compared with conventional stoichiometric or harsh synthetic protocols, metal-catalyzed methodologies provide improved efficiency, broader substrate scope, and access to diverse compound libraries from readily available precursors. The growing emphasis on sustainable catalysis has stimulated increasing interest in the use of earth-abundant first-row transition metals as alternatives to noble-metal catalysts in heterocycle synthesis. Beyond activity, many metal-catalyzed quinoline syntheses offer practical advantages, including high yields, operational simplicity, and reduced reaction severity. In several cases, the use of inexpensive and earth-abundant metals affords cost-effective and comparatively safer catalytic systems for quinoline construction [19,20,27,28,29,30,31,32,33].

### 2.1. Fe-Group Metals

Earth-abundant first-row transition metals such as Fe, Co, and Ni have emerged as attractive alternatives to precious-metal catalysts owing to their low cost, high natural abundance, and versatile redox properties.

Iron has the ability to facilitate a wide range of reactions and tolerate a wide range of functional groups. Iron(III) chloride (FeCl_3_) has been extensively used to promote oxidative annulation reactions in quinoline synthesis, playing a dual role as a catalyst and as an oxidizing agent [28,34].

**Scheme 1:**
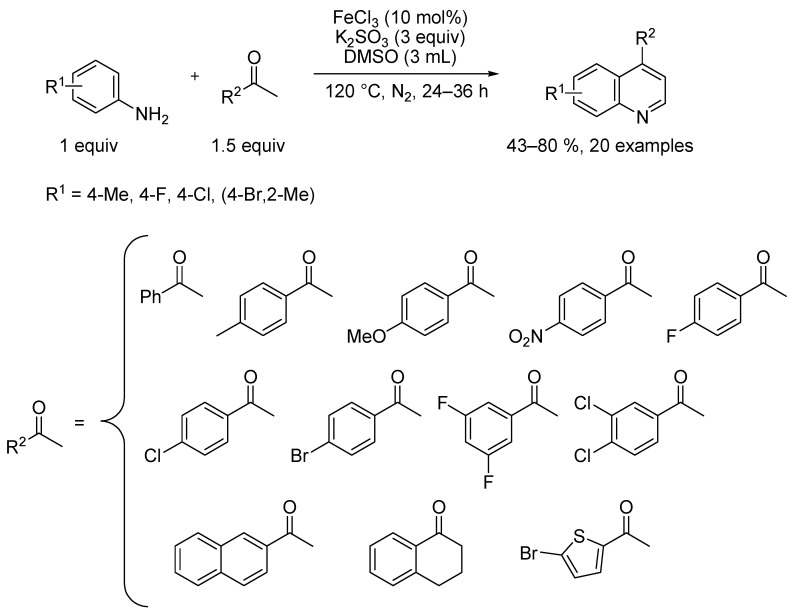
FeCl_3_-catalyzed oxidative synthesis of quinolines.

In 2017, Jadhav and Singh reported a transition-metal-free oxidative annulation strategy for the synthesis of 4-arylquinolines via a three-component coupling of anilines, acetophenones, and DMSO under K_2_S_2_O_8_-mediated conditions [35]. In this approach, DMSO plays a dual role as solvent and as a one-carbon (methine) synthon, enabling direct incorporation of a C1 unit into the quinoline framework. The optimized conditions allow the efficient formation of 4-arylquinolines from readily available starting materials, with moderate to good yields across a broad range of substrates. Electron-rich and electron-deficient acetophenones are tolerated, as are α-substituted ketones, while para-substituted anilines perform best under the oxidative conditions. The addition of FeCl_3_ significantly improves yields, particularly for electron-rich acetophenones, likely by facilitating enolization and modulating aniline nucleophilicity, thereby suppressing competitive side reactions (Scheme 1).

**Scheme 2:**
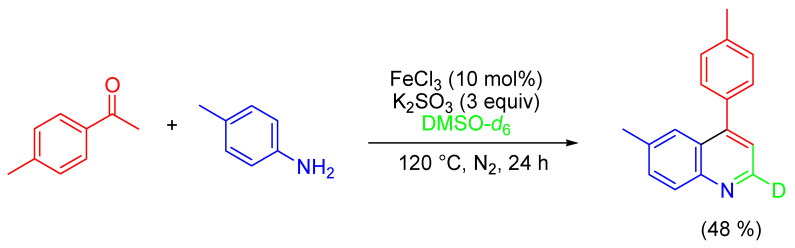
Deuterium incorporation using DMSO-*d*6.

Mechanistic experiments support a pathway initiated by persulfate-mediated activation of DMSO to generate a reactive sulfenium species, which is intercepted by aniline, forming an iminium intermediate. Subsequent C-C bond formation with the enol/enolate of the ketone, followed by cyclization, dehydration, and oxidative aromatization, furnishes the quinoline core. Deuterium-labeling studies using DMSO-*d*_6_ (deuterated dimethyl sulfoxide) afforded the deuterated quinoline derivative as shown in Scheme 2.

From a sustainability standpoint, this protocol avoids precious transition metals, employs inexpensive and readily available substrates, and constructs the quinoline scaffold through a cascade process that forms multiple bonds in a single operation. Although the use of stoichiometric persulfate and elevated temperatures may limit its overall greenness, the method represents a conceptually innovative and operationally simple approach to quinoline synthesis based on oxidative C-C and C-N bond-forming annulation.

**Scheme 3:**
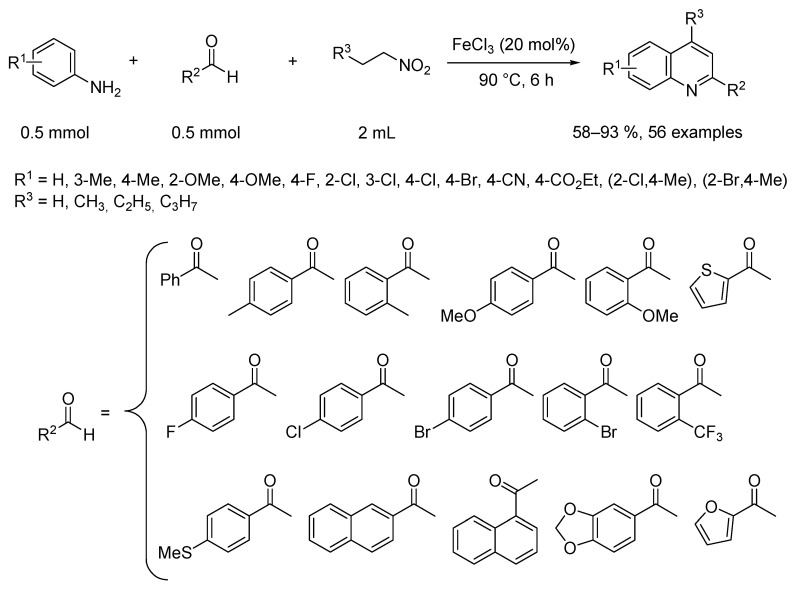
Iron(III)-catalyzed three-component synthesis of quinolines.

In 2019, Mahato and co-workers reported an iron(III)-catalyzed multicomponent cascade strategy for the synthesis of substituted quinolines through a one-pot reaction of anilines, aldehydes, and nitroalkanes [32]. The method employs a catalytic amount of FeCl_3_ under ambient air, enabling a domino sequence that integrates C-C and C-N bond formation in a single operation. The protocol proceeds efficiently from commercially available starting materials and furnishes a broad library of quinoline derivatives in generally high yields. The reaction is proposed to follow a sequential aza-Henry reaction, intramolecular cyclization, and denitration pathway, ultimately delivering the aromatized quinoline framework. The operational simplicity, mild aerobic conditions, and tolerance toward diverse functional groups highlight the robustness of the transformation (Scheme 3).

From a sustainability perspective, this approach is particularly attractive due to the use of an inexpensive, earth-abundant iron catalyst, avoidance of precious metals, and the multicomponent domino design that minimizes purification steps and waste generation. The one-pot nature of the process enhances atom economy and step efficiency, while the use of readily available feedstocks broadens its practical applicability.

**Scheme 4:**
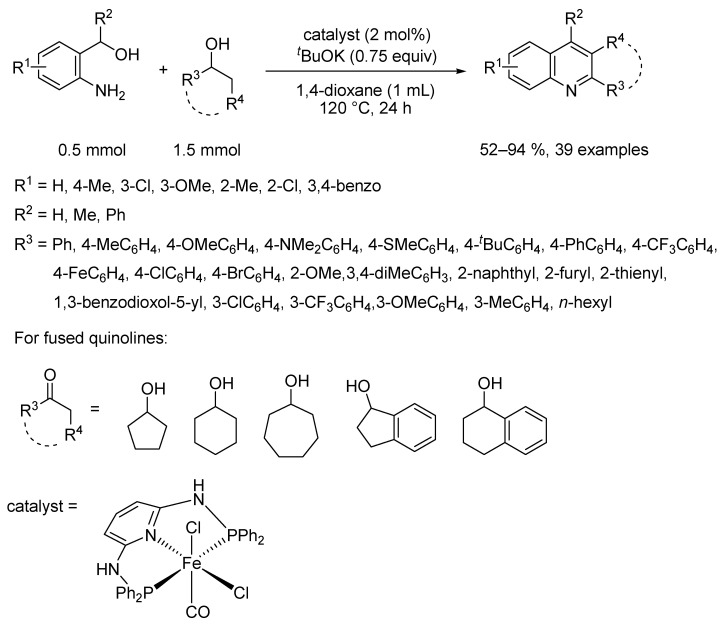
Iron-catalyzed quinoline synthesis via ADC.

Recent advances in homogeneous iron catalysis have also demonstrated the potential of earth-abundant iron complexes for sustainable quinoline synthesis through acceptorless dehydrogenative coupling (ADC) pathways. In a 2022 study, Yu and co-workers reported the synthesis of quinoline derivatives from 2-aminobenzyl alcohols and ketones catalyzed by an iron PNP pincer complex under relatively mild conditions [34]. The methodology proceeds through a borrowing-hydrogen/ADC mechanism in which the amino alcohol substrate is initially dehydrogenated to the corresponding amino aldehyde, followed by condensation with the ketone partner, cyclization, and final aromatization to furnish the quinoline core. The catalytic system afforded a broad substrate scope and good to excellent yields while generating molecular hydrogen as the sole stoichiometric by-product, thereby improving atom economy and sustainability (Scheme 4).

Mechanistic investigations supported the involvement of iron hydride intermediates and metal–ligand cooperative processes characteristic of PNP pincer complexes. Notably, this type of ADC strategy has recently become a central approach in quinoline synthesis using pincer-type catalysts based on platinum-group metals such as ruthenium, iridium, and palladium, which are discussed in detail in a subsequent subsection. Overall, this work highlights the growing potential of homogeneous iron catalysis as a cost-effective and environmentally benign alternative to noble-metal systems for quinoline synthesis.

**Scheme 5:**
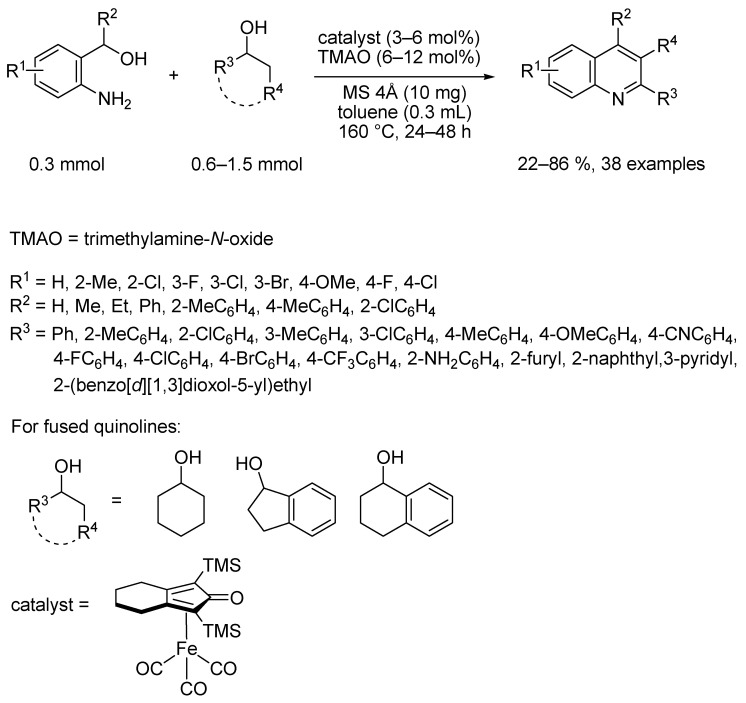
Iron-catalyzed acceptorless dehydrogenative annulation.

Building upon earlier borrowing-hydrogen strategies based on iron PNP pincer complexes, recent studies have further expanded the role of homogeneous iron catalysis in quinoline synthesis through hydrogen-transfer methodologies. In a 2023 study, researchers developed an iron-catalyzed acceptorless dehydrogenative annulation protocol for the synthesis of quinoline derivatives from o-aminobenzyl alcohols and ketones under relatively mild conditions [36]. The methodology operates through sequential alcohol dehydrogenation, condensation, cyclization, and aromatization steps, generating molecular hydrogen as the sole stoichiometric by-product. The catalytic system displayed broad substrate tolerance and provided access to structurally diverse quinolines in generally good yields while employing an earth-abundant and comparatively low-toxicity metal catalyst (Scheme 5).

Mechanistic investigations suggested the involvement of iron hydride intermediates and metal–ligand cooperative processes characteristic of hydrogen-transfer catalysis. Importantly, this work highlights the increasing convergence between homogeneous iron catalysis and catalytic paradigms traditionally dominated by platinum-group metals, particularly borrowing-hydrogen and acceptorless dehydrogenative coupling strategies based on pincer-type Ru, Ir, and Pd complexes discussed in the following subsection.

Among earth-abundant first-row transition metals, cobalt has emerged as a particularly attractive platform for homogeneous catalysis owing to its low cost, relatively low toxicity, variable oxidation states, and rich coordination chemistry. In recent years, cobalt complexes have demonstrated remarkable potential in borrowing-hydrogen and acceptorless dehydrogenative coupling (ADC) methodologies, enabling the efficient synthesis of quinoline derivatives from readily available alcohols and ketones while generating only H_2_ and/or H_2_O as by-products. These transformations frequently rely on metal–ligand cooperative catalysis involving pincer or pincer-like ligand frameworks, often incorporating redox-active ligands that facilitate hydrogen-transfer events and stabilize reactive intermediates. Consequently, homogeneous cobalt catalysis has evolved into an important and rapidly developing area within sustainable quinoline synthesis.

**Scheme 6:**
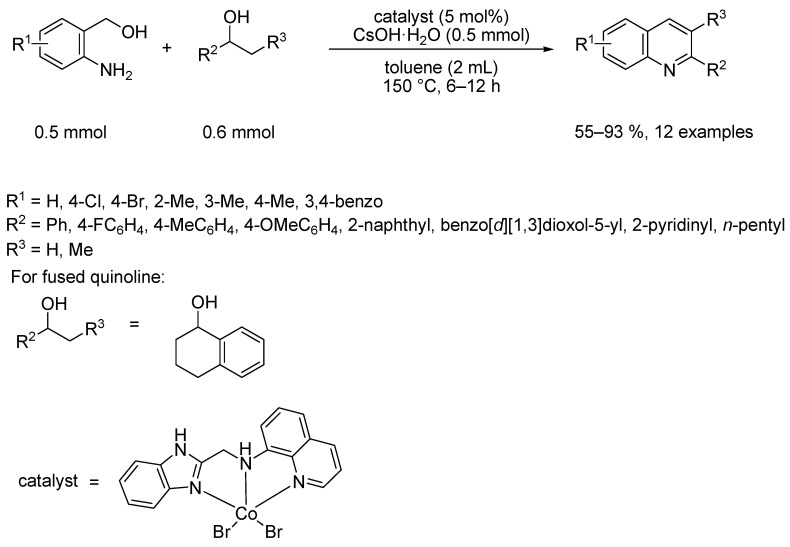
Co-catalyzed synthesis of quinolines via dehydrogenative coupling.

One of the earliest examples of homogeneous cobalt-catalyzed quinoline synthesis through dehydrogenative methodologies was reported by Shee et al. in 2018 [37], who developed a phosphine-free Co(II) complex-catalyzed atom-economical protocol for the synthesis of quinolines via dehydrogenative coupling reactions. The catalytic system enabled the efficient annulation of 2-aminobenzyl alcohols with ketones through tandem alcohol dehydrogenation, condensation, cyclization, and aromatization steps (Scheme 6).

The methodology demonstrated broad substrate scope and good functional-group tolerance while employing a base-metal catalyst under relatively mild conditions. Importantly, the transformation generated molecular hydrogen as the sole stoichiometric by-product, illustrating the sustainability advantages of ADC-based quinoline synthesis. This work established cobalt as a viable alternative to Ru- and Ir-based systems in hydrogen-transfer heterocycle synthesis.

**Scheme 7:**
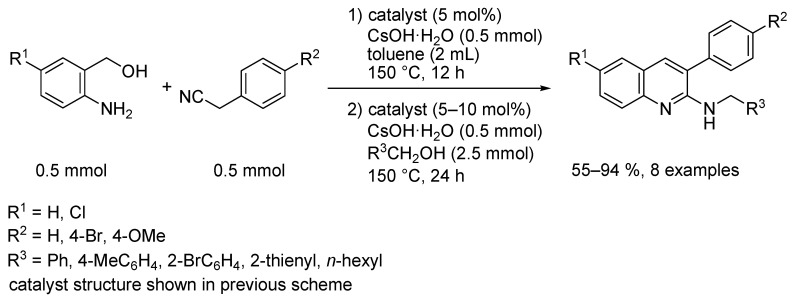
Co-catalyzed one-pot synthesis of 2-alkylaminoquinolines.

The catalytic system was also successfully applied to the one-pot synthesis of 2-alkylaminoquinolines from 2-aminobenzyl alcohol, benzyl cyanide, and benzyl alcohol, affording the desired product in excellent yields. The transformation proceeds through an acceptorless dehydrogenative annulation followed by *N*-alkylation. This study represents the first reported example of Co(II)-catalyzed synthesis of 2-alkylaminoquinolines via this strategy, highlighting the potential of cobalt catalysts as cost-effective alternatives to Ru(II)-based systems (Scheme 7).

**Scheme 8:**
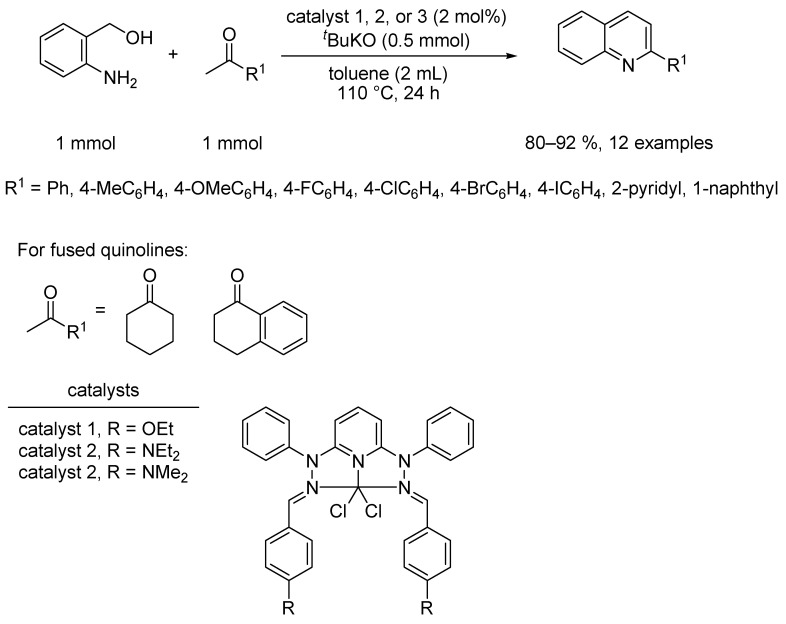
Co-catalyzed dehydrogenative cyclization of 2-amino benzyl alcohol.

Subsequent developments focused increasingly on ligand-enabled cobalt catalysis. In 2021, Singh and co-workers reported a series of pincer-ligand-supported Co(II) complexes capable of promoting alcohol dehydrogenation and quinoline synthesis through borrowing-hydrogen pathways [38]. The catalytic systems efficiently mediated the coupling of 2-aminobenzyl alcohols with ketones, providing quinoline derivatives in good yields with any of the three catalysts tested (Scheme 8).

Mechanistic investigations indicated that the catalytic activity relied strongly on metal–ligand cooperative effects, particularly involving reversible deprotonation processes within the ligand framework that facilitate hydrogen transfer during the catalytic cycle.

**Scheme 9:**
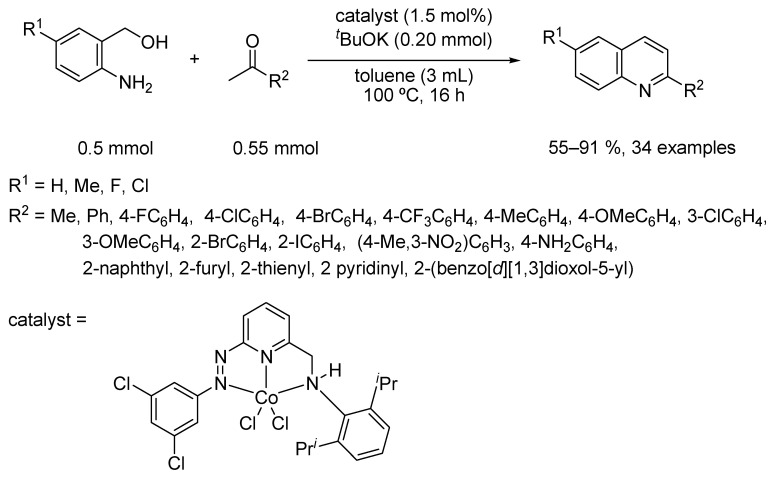
Phosphine-free cobalt(II)-catalyzed synthesis of quinolines.

In a 2023 study, researchers reported a family of phosphine-free pincer-like azo-aromatic cobalt(II) complexes as efficient homogeneous catalysts for quinoline synthesis dehydrogenative oxidation of 2-aminobenzyl alcohol and subsequent coupling with a ketone [39]. The catalytic system enabled the annulation of 2-aminobenzyl alcohols with ketones under relatively mild conditions, affording a broad range of substituted quinolines in moderate to excellent yields (Scheme 9).

Mechanistic studies indicated that the redox-active azo-pyridine ligand governs the catalytic activity by promoting hydrogen-atom abstraction from the α-C-H bond of the alcohol substrate. Subsequent Co(II)/Co(I) reduction generates the corresponding aldehyde, which undergoes base-mediated C-C bond formation and cyclization to afford quinolines. Notably, the catalytic cycle involves a low-valent Co(I) intermediate, and the ligand-assisted radical alcohol oxidation pathway represents a relatively unexplored mode of cobalt catalysis.

**Scheme 10:**
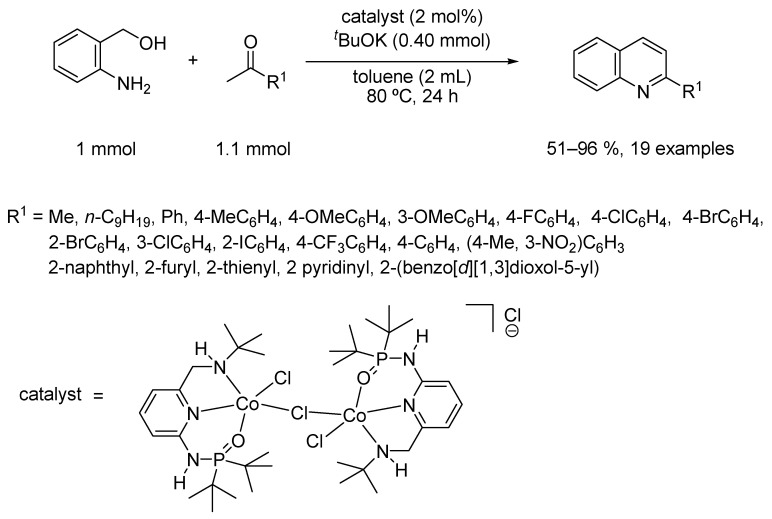
Phosphine-oxide cobalt(II)-catalyzed synthesis of quinolines.

In a 2024 study, the same group developed phosphine-oxide-derived cobalt(II) pincer complexes as efficient homogeneous catalysts for the synthesis of quinoline derivatives through acceptorless dehydrogenative coupling reactions [40]. The catalytic system promoted the annulation of 2-aminobenzyl alcohols with a broad range of ketones under relatively mild conditions, affording substituted quinolines in good to excellent yields. The methodology tolerated electron-donating and electron-withdrawing aromatic ketones, heteroaromatic substrates, and aliphatic ketones, demonstrating broad substrate scope and good functional-group compatibility. Notably, the phosphine-oxide-containing cobalt complex exhibited enhanced catalytic stability and activity under aerobic conditions compared with related phosphine-based systems, an uncommon feature for phosphine-derived transition-metal catalysts (Scheme 10).

Mechanistic investigations combining experimental observations and DFT calculations revealed that α-hydrogen abstraction constitutes the rate-determining step under aerobic conditions. The study further demonstrated that the phosphine oxide ligand acts as a pincer-type coordinating framework throughout the catalytic cycle. Although phosphine-derived pincer complexes have been extensively explored in catalysis, the corresponding phosphine oxide analogues remain largely unexplored.

**Scheme 11:**
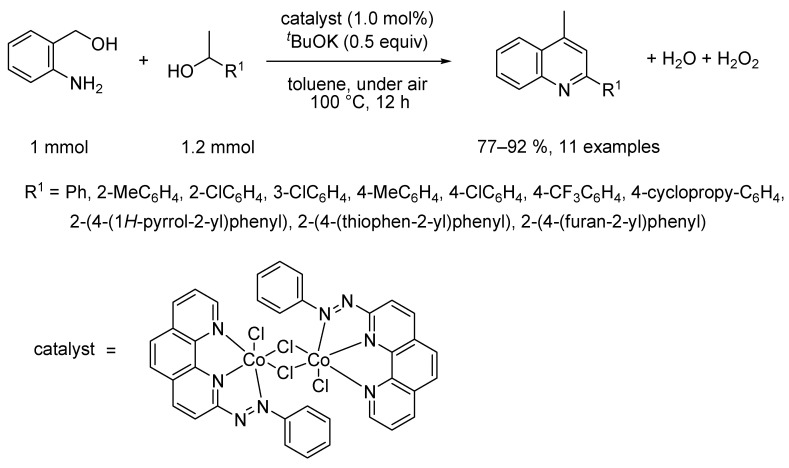
Co-catalyzed synthesis of substituted quinolines.

Using a well-defined Co(II) complex bearing a 2-(phenyldiazenyl)-1,10-phenanthroline ligand, Monda et al. achieved efficient synthesis of quinoline derivatives through borrowing-hydrogen catalysis involving 2-aminobenzyl alcohols and secondary alcohols [41]. Detailed mechanistic studies suggested that the reaction proceeds through a hydride-transfer pathway in which the azo-aromatic ligand acts cooperatively as a hydrogen-storage reservoir. Control experiments, isotopic-labeling studies, and spectroscopic investigations supported the involvement of transient ligand-assisted hydrazido intermediates during alcohol dehydrogenation and substrate hydrogenation steps. The work demonstrated the potential of redox-noninnocent ligand frameworks to expand the catalytic capabilities of cobalt complexes in sustainable heterocycle synthesis (Scheme 11).

**Scheme 12:**
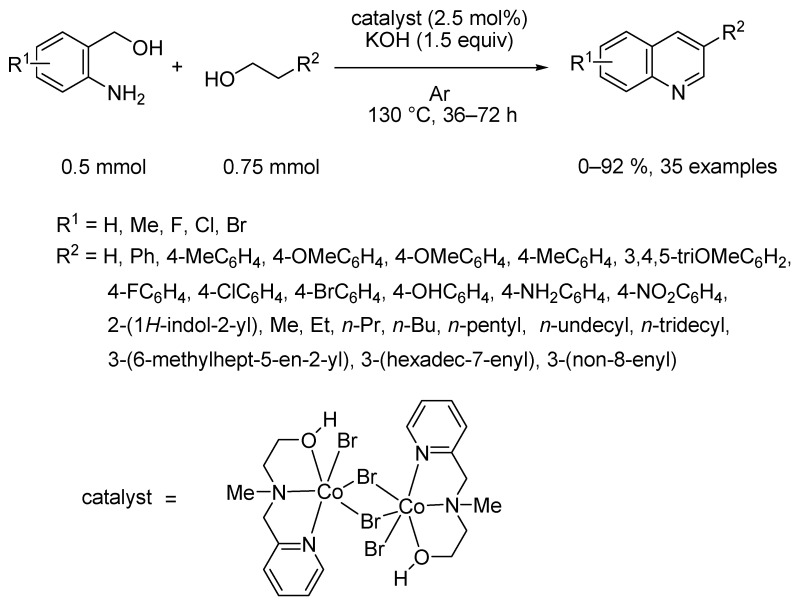
Cobalt(II)-catalyzed tandem dehydrogenative synthesis of quinolines.

In another 2024 contribution, Pal et al. designed structurally tunable Co(II) complexes for the dehydrogenative synthesis of C-3-substituted quinoline and quinazoline derivatives [42]. The catalysts enabled efficient annulation of 2-aminobenzyl alcohols with primary alcohols through tandem dehydrogenation–condensation sequences, providing a broad range of heterocycles in high yields. Importantly, the methodology addressed a challenging synthetic problem, namely the direct preparation of C-3-substituted quinolines from primary alcohol feedstocks, which is often complicated by competing Guerbet-type alcohol coupling reactions. The catalytic system also tolerated unsaturated alcohol substrates, allowing selective heterocycle synthesis without hydrogenation of distal C=C bonds (Scheme 12).

Mechanistic studies indicated that steric modulation of the ligand framework strongly influences catalytic reactivity and selectivity, further illustrating the central role of ligand design in homogeneous cobalt catalysis.

**Scheme 13:**
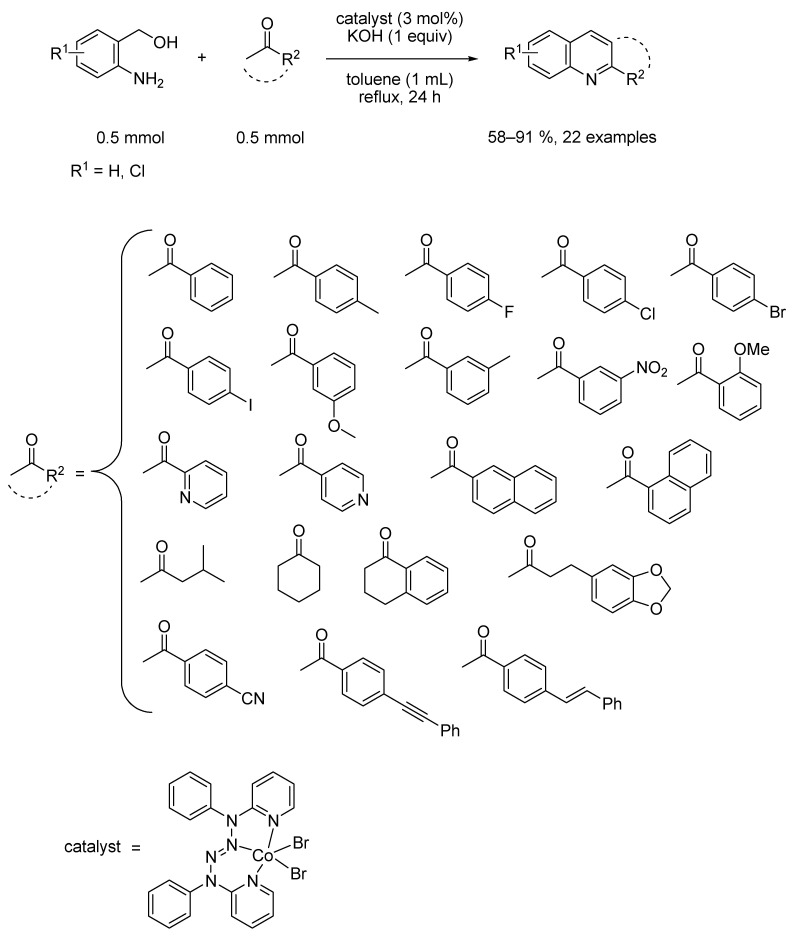
Synthesis of quinolines via co-catalyzed dehydrogenative coupling reaction.

The same year, another homogeneous cobalt-catalyzed strategy for the synthesis of quinoline derivatives through acceptorless dehydrogenative annulation reactions employing readily accessible alcohol feedstocks was reported by Kumari and co-workers [43]. The methodology relies on well-defined cobalt complexes capable of promoting tandem alcohol dehydrogenation, condensation, cyclization, and aromatization processes under relatively mild conditions, affording a broad range of substituted quinolines in moderate to excellent yields. The catalytic system demonstrated good tolerance toward both aromatic and aliphatic substrates, including electron-rich and electron-deficient substituents, highlighting the versatility of the transformation (Scheme 13).

Mechanistic investigations suggested that the reaction proceeds through borrowing-hydrogen pathways involving cobalt-hydride intermediates and metal–ligand cooperative hydrogen-transfer steps. Importantly, the methodology generates molecular hydrogen as the sole stoichiometric by-product, thereby improving atom economy and sustainability. This work further reinforces the growing relevance of homogeneous cobalt catalysis as an environmentally benign alternative to noble-metal systems for quinoline synthesis and highlights the continued expansion of first-row transition-metal borrowing-hydrogen methodologies in heterocyclic chemistry.

Overall, recent developments clearly demonstrate the growing maturity of homogeneous cobalt catalysis for quinoline synthesis. Modern cobalt systems increasingly rely on borrowing-hydrogen and acceptorless dehydrogenative coupling strategies enabled by sophisticated pincer and redox-active ligand architectures. These catalysts combine the advantages of earth-abundant metals with atom-economical hydrogen-transfer methodologies traditionally dominated by ruthenium and iridium complexes. Moreover, mechanistic studies have revealed diverse catalytic paradigms involving hydride transfer, ligand-centered redox activity, hydrogen atom transfer, and metal–ligand cooperation. Collectively, these advances position homogeneous cobalt catalysis as one of the most promising sustainable approaches for the future development of quinoline synthesis.

**Figure 3:**
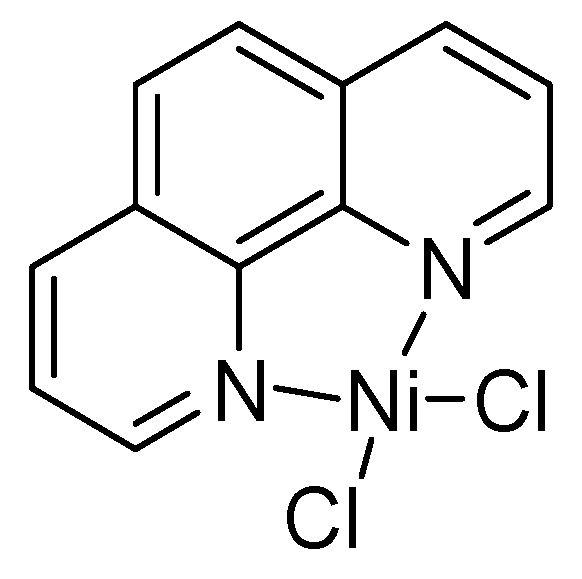
Ni-catalyst generated in situ in the reaction mixture.

The expansion of borrowing-hydrogen methodologies beyond iron has also been demonstrated using homogeneous nickel catalysis. In a 2023 study, researchers reported a nickel-catalyzed dehydrogenative annulation strategy for quinoline synthesis based on hydrogen-transfer processes involving *o*-aminobenzyl alcohols and ketones [44]. The catalytic transformation proceeds through tandem alcohol dehydrogenation, condensation, cyclization, and aromatization steps, affording a variety of quinoline derivatives under relatively mild conditions without requiring external oxidants. The catalyst is generated in situ from NiCl_2_·DME and 1,10-phenanthroline (Figure 3).

**Scheme 14:**
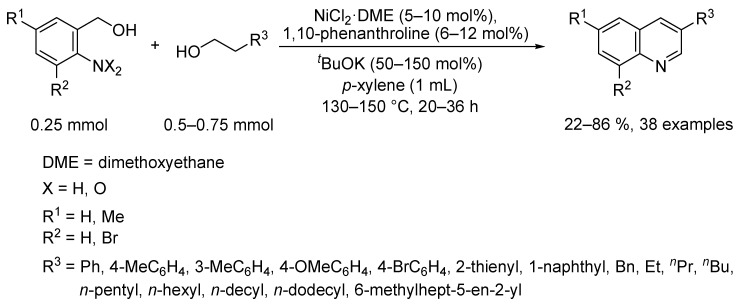
Nickel-catalyzed synthesis of quinolines.

The methodology broadens the use of first-row transition metals in acceptorless dehydrogenative quinoline synthesis, an area previously dominated by ruthenium and iridium catalysts (Scheme 14).

Mechanistic studies suggested that the nickel catalyst mediates hydrogen-transfer events through transient Ni-hydride intermediates, enabling efficient annulation while generating molecular hydrogen as the only stoichiometric by-product. The use of an earth-abundant and inexpensive metal further reinforces the growing interest in replacing noble-metal catalysts with more sustainable first-row transition-metal systems for quinoline synthesis.

In conclusion, the increasing demand for sustainable catalytic methodologies has stimulated growing interest in the use of earth-abundant first-row transition metals such as iron, cobalt, and nickel as alternatives to noble-metal catalysts in quinoline synthesis. In recent years, these metals have demonstrated remarkable potential in hydrogen-transfer, borrowing-hydrogen, and acceptorless dehydrogenative coupling (ADC) strategies, enabling efficient quinoline formation through tandem dehydrogenation–condensation–cyclization sequences. The development of pincer-type and redox-active ligand frameworks has played a central role in expanding the catalytic capabilities of Fe-group metals, allowing mechanistic paradigms traditionally associated with Ru, Ir, and Pd catalysis to be extended to more sustainable and economically attractive catalytic systems. These advances also illustrate the growing convergence between first-row and platinum-group-metal catalysis in modern quinoline synthesis, particularly in the context of atom-economical hydrogen-transfer methodologies discussed in the following section.

### 2.2. Pt-Group Metals

Platinum-group metal-based homogeneous catalysts have attracted considerable attention in the synthesis of quinoline derivatives due to their high catalytic activity, tunable electronic properties, and ability to promote multiple bond-forming steps within a single catalytic cycle. Among them, ruthenium complexes have attracted more attention than other metals in this group. They can operate through different oxidation states, most commonly Ru(II) and Ru(III), and are particularly effective in facilitating dehydrogenation, condensation, and cyclization processes. Pincer ligands have emerged as a prominent class of supporting ligands in coordination and organometallic chemistry owing to their synthetic accessibility, structural versatility, and strong coordination ability. These tridentate ligands typically adopt a meridional coordination mode, providing a rigid and well-defined environment around the metal center [45,46]. The strong chelating character of pincer frameworks affords highly stable transition-metal complexes, making them particularly attractive for catalytic applications. Over the past decades, pincer complexes have attracted considerable attention in homogeneous catalysis because their steric and electronic properties can be readily tuned through ligand design, enabling the development of efficient and selective catalytic systems [47]. In particular, transition-metal complexes bearing NNN- and PNP-type pincer ligands have shown remarkable activity in a wide range of organic transformations, including C-C and C-N bond-forming reactions [48]. The robustness, tunability, and catalytic efficiency of these complexes have therefore established pincer ligands as key platforms for the design of sustainable and high-performance catalysts for organic synthesis. The incorporation of multidentate ligands, especially pincer-type frameworks, has significantly enhanced catalyst performance by enabling metal–ligand cooperativity (MLC), which plays a key role in hydrogen transfer steps and substrate activation [49].

Recent developments in this area have been largely driven by the implementation of acceptorless dehydrogenative coupling (ADC) strategies. In these methodologies, alcohols are employed as starting materials, offering advantages in terms of availability, safety, and potential renewability. Compared to classical quinoline syntheses, which often require prefunctionalized carbonyl compounds and stoichiometric oxidants, ADC approaches are inherently more atom-efficient, as they proceed without external oxidants and generate only hydrogen and water as by-products.

A representative example is the use of triazine-based NNN-pincer ruthenium complexes for the synthesis of quinolines via coupling of 2-aminobenzyl alcohols with secondary alcohols [50]. Under optimized conditions (typically 120 °C, aerobic atmosphere), these systems provide high yields with relatively low catalyst loadings (down to 0.1 mol%) and short reaction times. Notably, very high turnover numbers (TONs), up to 440,000, have been reported, highlighting the efficiency of these catalytic systems.

**Scheme 15:**
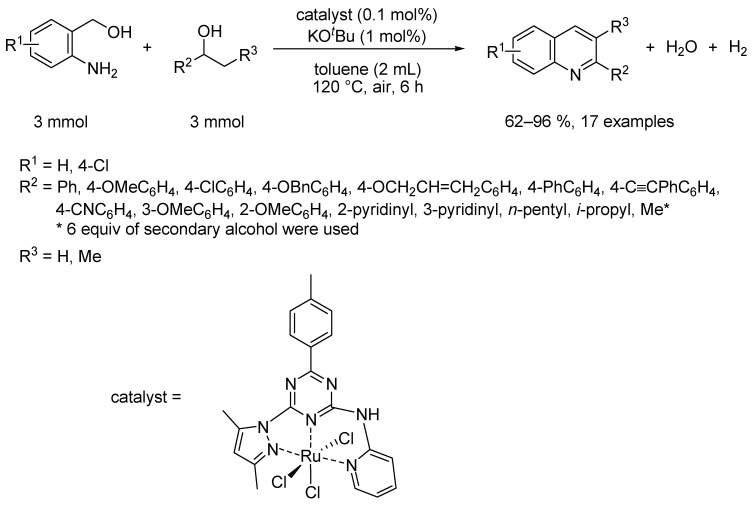
Ruthenium pincer complex catalyzed synthesis of quinolines.

The substrate scope of such methodologies is generally broad. Secondary alcohols bearing electron-donating or electron-withdrawing substituents are well tolerated, and the reaction can accommodate heteroaromatic and aliphatic substrates. Furthermore, good functional group tolerance has been observed, including the preservation of reducible groups such as alkenes, alkynes, and nitriles. This versatility enhances the applicability of ruthenium-catalyzed protocols in the synthesis of structurally diverse quinoline derivatives (Scheme 15).

Mechanistic studies indicate that these transformations proceed through initial dehydrogenation of alcohol substrates to generate the corresponding aldehydes or ketones, followed by condensation with 2-aminobenzyl alcohol-derived intermediates and subsequent cyclization. The catalytic cycle typically involves the formation of ruthenium hydride species and relies on metal–ligand cooperative pathways, where ligand functionalities (e.g., -NH groups) facilitate proton and hydride transfer. Such cooperative effects are essential for achieving high catalytic efficiency.

**Scheme 16:**
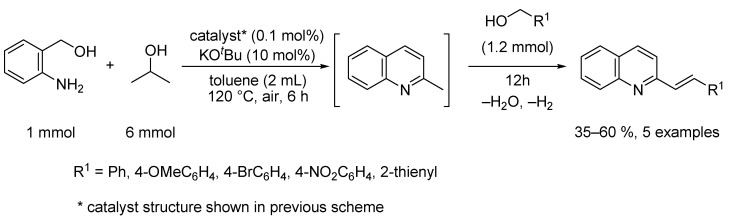
Ruthenium-catalyzed three-component synthesis of 2-styrylquinolines.

In addition to two-component reactions, ruthenium catalysts have also been applied in multicomponent and tandem processes. For example, one-pot three-component reactions involving 2-aminobenzyl alcohols, secondary alcohols, and primary alcohols have been developed for the synthesis of 2-styrylquinoline derivatives. These transformations improve step economy and reduce the need for intermediate isolation, contributing to more sustainable synthetic protocols (Scheme 16).

**Scheme 17:**
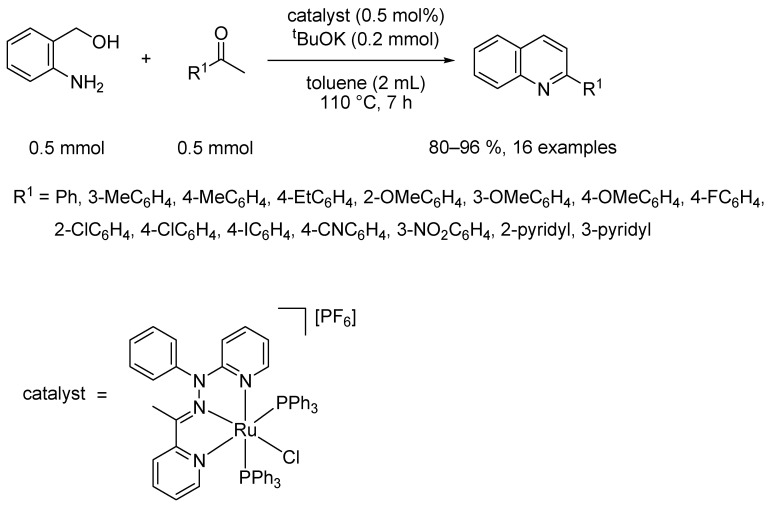
Synthesis of Quinolines by Dehydrogenative Reaction.

Recent advances in homogeneous platinum-group-metal catalysis have further highlighted the effectiveness of ruthenium pincer-like complexes for sustainable quinoline synthesis through borrowing-hydrogen and acceptorless dehydrogenative coupling pathways. In a 2023 study, researchers reported a family of Ru pincer-like complexes capable of efficiently promoting the annulation of *o*-aminobenzyl alcohols with ketones to afford quinoline derivatives under relatively mild conditions [51]. The catalytic methodology proceeds through tandem alcohol dehydrogenation, condensation, cyclization, and final aromatization steps, generating molecular hydrogen as the sole stoichiometric by-product. A broad range of quinoline derivatives bearing electron-donating, electron-withdrawing, and heteroaromatic substituents was synthesized in good to excellent yields, demonstrating good substrate tolerance and catalytic versatility (Scheme 17).

Mechanistic investigations suggested that the Ru complexes operate through metal–ligand cooperative processes involving Ru-hydride intermediates characteristic of borrowing-hydrogen catalysis. The pincer-like ligand framework was found to play a crucial role in stabilizing catalytic intermediates and facilitating hydrogen-transfer events throughout the catalytic cycle. Overall, this work further reinforces the growing importance of Ru-based pincer architectures in the development of atom-economical and sustainable methodologies for quinoline synthesis.

**Scheme 18:**
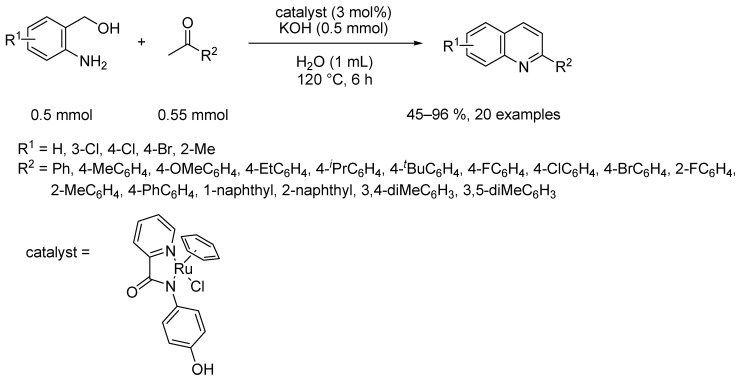
Quinoline Synthesis Catalyzed by Ruthenium Complexes in Water.

Further advances include the development of water-soluble ruthenium catalysts, such as functionalized amidato complexes, which facilitate a homogeneous ruthenium-catalyzed strategy for the synthesis of quinoline derivatives through an acceptorless dehydrogenative annulation process in aqueous media [52]. The use of water as a solvent represents a significant step toward greener processes, reducing reliance on volatile organic solvents and improving safety profiles. In these systems, ligand design plays a critical role in maintaining catalyst stability and activity in aqueous environments, while also promoting efficient alcohol dehydrogenation pathways. The methodology employed Ru-based catalytic systems to promote tandem alcohol dehydrogenation, condensation, cyclization, and aromatization steps, enabling the efficient construction of quinoline frameworks from readily available aminoaryl alcohols and ketone substrates under comparatively mild conditions. The protocol provided a broad substrate scope and afforded the desired quinolines in good to excellent yields while generating molecular hydrogen as the sole stoichiometric by-product, thereby improving atom economy and sustainability (Scheme 18).

Mechanistic investigations suggested that the reaction proceeds through initial Ru-catalyzed dehydrogenation of the alcohol substrate to the corresponding carbonyl intermediate, followed by imine formation, intramolecular cyclization, and final dehydrogenative aromatization.

**Scheme 19:**
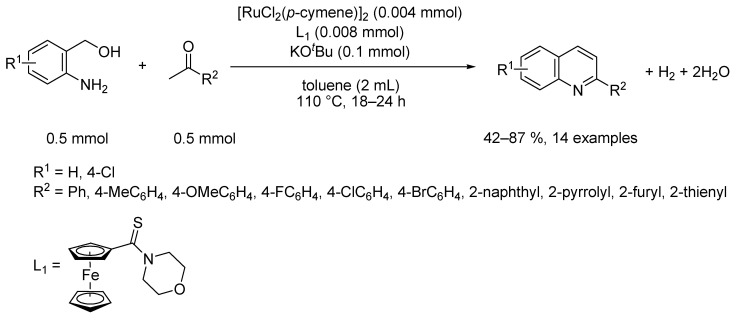
Synthesis of quinolines via mono dehydrogenation reaction.

**Scheme 20:**
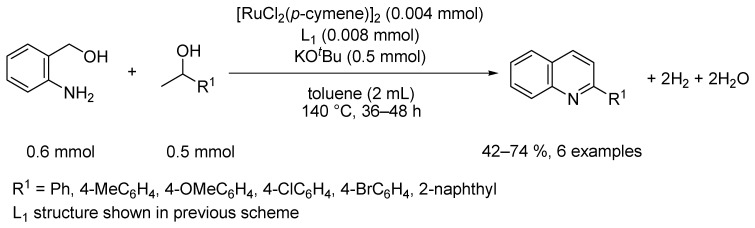
Synthesis of quinolines via double dehydrogenation reaction.

Building upon earlier Ru-catalyzed acceptorless dehydrogenative annulation methodologies, Ravindran and Karvembu developed a homogeneous Ru(II)-based catalytic system for the synthesis of quinoline derivatives through both mono- and double-dehydrogenative coupling pathways involving 2-aminobenzyl alcohol with ketones or secondary alcohols [53]. The catalytic protocol employed in situ-generated Ru(II)-*p*-cymene complexes bearing ferrocenyl thioamide ligands, with the morpholine-substituted ligand providing the highest catalytic activity. Under optimized conditions, a broad range of aromatic, heteroaromatic, and substituted ketones or alcohols underwent efficient annulatio to furnish quinoline derivatives in moderate to excellent yields *via* mono dehydrogenation (Scheme 19) or double dehydrogenation (Scheme 20).

Mechanistic studies indicated that the Ru catalyst promotes initial dehydrogenation of 2-aminobenzyl alcohol to 2-aminobenzaldehyde via Ru-alkoxide and Ru-hydride intermediates, followed by aldol-type condensation, cyclization, and final aromatization to generate the quinoline core. Control experiments demonstrated a synergistic effect between the Ru precursor and the Fc-TA ligand, while computational studies supported the in situ formation of the active catalytic species. Notably, the methodology proceeds through borrowing-hydrogen/acceptorless dehydrogenation pathways without requiring external oxidants, reinforcing the growing relevance of homogeneous Ru catalysis for sustainable quinoline synthesis.

**Scheme 21:**
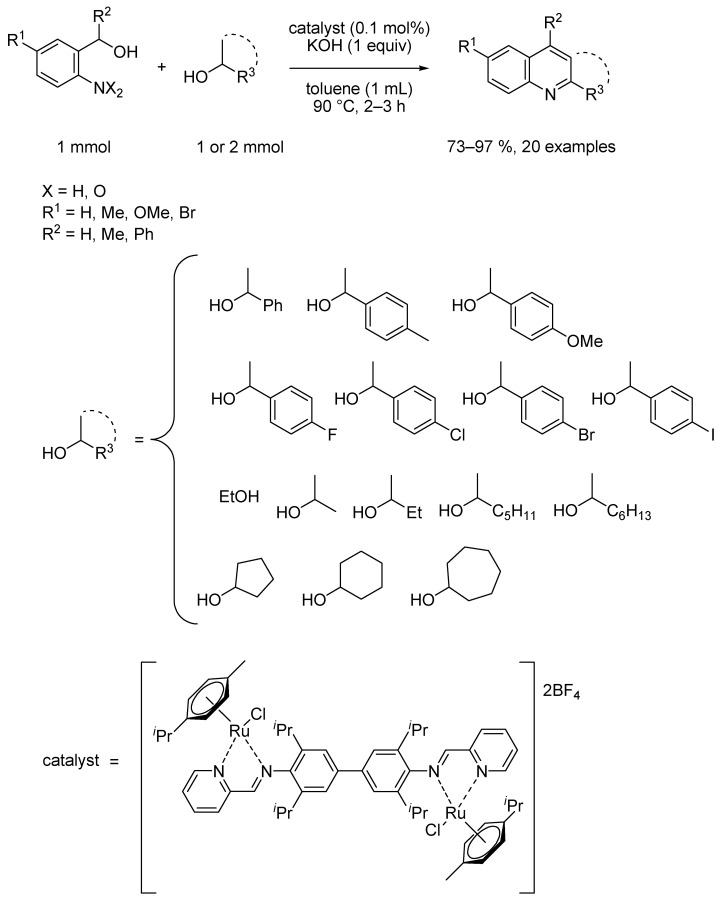
Dinuclear Ru(II) Complex-Catalyzed Synthesis of Quinolines.

Further expanding the scope of homogeneous Ru-catalyzed quinoline synthesis, Deshmukh and co-workers reported a highly efficient dinuclear Ru(II) catalytic system for the direct synthesis of quinolines from 2-nitrobenzyl alcohols or 2-aminobenzyl alcohols and secondary alcohols through tandem transfer-hydrogenation and dehydrogenative annulation pathways [54]. The catalysis was mediated by air- and moisture-stable dinuclear *p*-cymene Ru(II) complexes bearing Schiff-base ligands, with one catalyst exhibiting outstanding activity even at extremely low catalyst loadings. Under optimized conditions, a wide variety of quinoline derivatives, including structurally complex and steroid-modified substrates, were obtained in good to excellent yields under relatively mild conditions (90 °C) without the need for external oxidants, high-pressure hydrogen, or phosphine ligands. Remarkably, catalyst loadings as low as 0.001 mol % afforded turnover numbers up to 67,000 and turnover frequencies of 22,333 h^−1^, highlighting the exceptional efficiency of the dinuclear Ru system (Scheme 21).

Mechanistic investigations supported a catalytic cycle involving Ru-mediated β-hydride elimination from both the secondary alcohol and the 2-nitrobenzyl alcohol substrates to generate carbonyl intermediates and Ru-hydride species, followed by transfer hydrogenation of nitroso intermediates, aldol-type condensation, cyclization, and final aromatization to furnish the quinoline core. Spectroscopic, GC-MS, ESI-MS, and in situ ReactIR studies further suggested that cooperative interactions between the two Ru centers facilitate the generation of the active catalytic species and enhance overall catalytic performance relative to analogous mononuclear systems.

**Scheme 22:**
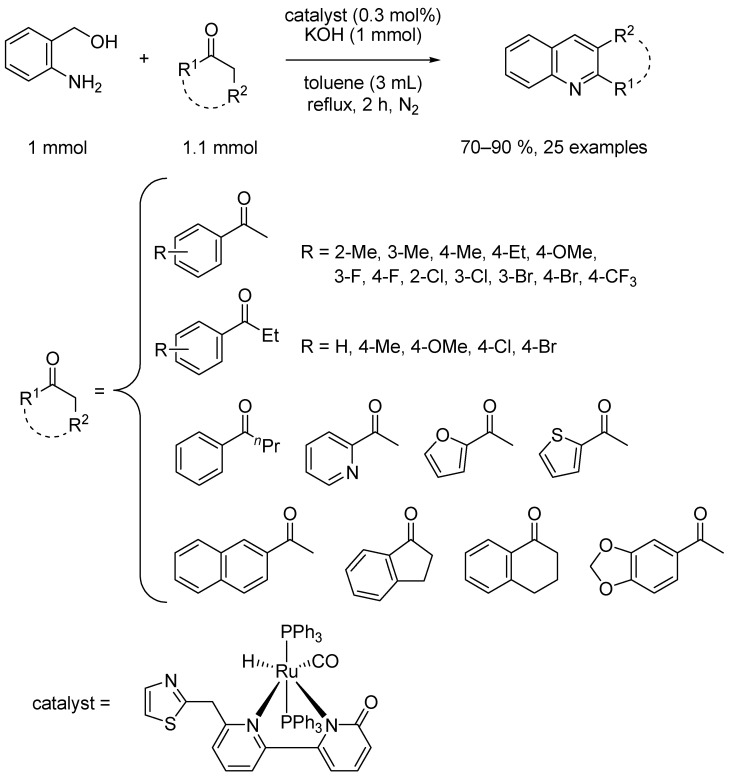
Bidentate ruthenium(II) complex-catalyzed synthesis of quinolines.

The recent work by Zhang and co-workers describes an efficient ruthenium(II)-catalyzed acceptorless dehydrogenative condensation (ADC) methodology for the synthesis of quinoline derivatives from *o*-aminobenzyl alcohols and ketones [55]. Using a series of bidentate Ru(II) complexes bearing 2-pyridonate or 2-hydroxypyridyl ligands, the authors identified the complex bearing 2-pyridonate with a pendant thiazolyl group as the most active catalyst for the transformation. Under optimized conditions, a broad range of quinolines was synthesized in good to excellent yields (Scheme 22). The protocol tolerated both electron-donating and electron-withdrawing substituents on the ketone component, including halogenated, heteroaryl, and fused aromatic substrates, demonstrating considerable substrate versatility. Sterically hindered substrates showed only a moderate decrease in efficiency.

Mechanistically, the methodology follows a borrowing-hydrogen/ADC pathway in which the amino alcohol undergoes dehydrogenation to generate the corresponding amino aldehyde, followed by condensation with ketones and subsequent cyclization/aromatization, with H_2_ and H_2_O as the only by-products. The presence of pendent *N*-heterocyclic donor groups in the catalyst framework was found to significantly enhance catalytic activity, likely through metal–ligand cooperative effects.

It is worth noting that the catalytic system was not limited to quinoline synthesis. By varying the amino alcohol substrate, the same Ru(II) catalyst could also be applied to the preparation of 1,8-naphthyridines, pyridines, and pyrroles in moderate to high yields. This work therefore expands the scope of ruthenium-catalyzed ADC reactions and highlights the utility of multifunctional Ru(II) complexes for the sustainable synthesis of diverse *N*-heterocyclic frameworks under relatively mild and atom-economical conditions.

Collectively, these studies highlight the continuing evolution of homogeneous Ru catalysis toward increasingly sustainable and mechanistically sophisticated methodologies for quinoline synthesis, emphasizing the key roles of hydrogen borrowing, metal–ligand cooperation, and tandem catalytic processes in modern heterocycle construction.

**Scheme 23:**
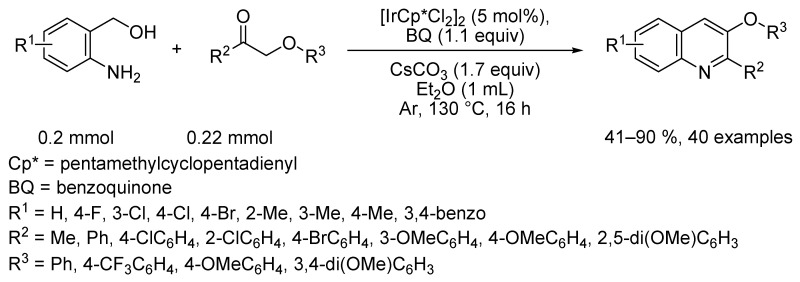
Iridium-catalyzed oxidative dehydrogenative annulation.

Recent advances in platinum-group-metal catalysis have also highlighted the potential of homogeneous iridium systems for sustainable quinoline synthesis through dehydrogenative annulation strategies. Cheng Zhang and co-workers describe an iridium-catalyzed dehydrogenative annulation strategy for the synthesis of 3-oxo quinoline derivatives from lignin model compounds (phenoxy acetophenones) and 2-aminobenzyl alcohols [56]. The methodology represents a sustainable approach for quinoline construction because it employs lignin-derived substrates as renewable feedstocks and avoids the use of unstable *o*-aminobenzaldehydes traditionally required in Friedländer-type syntheses. The optimized reaction conditions employed [IrCp*Cl_2_]_2_ as catalyst, CsCO_3_ as base, and benzoquinone as hydrogen acceptor in Et_2_O at 130 °C under argon atmosphere. A broad substrate scope was investigated using differently substituted phenoxy acetophenones and 2-aminobenzyl alcohols. Products were obtained in moderate to good yields (Scheme 23).

The study demonstrated that both the iridium catalyst and BQ were essential for efficient cyclization, while reactions conducted under air still afforded moderate yields, highlighting the robustness of the protocol. An important advantage of this methodology is the direct synthesis of 3-oxo-substituted quinolines, a relatively underexplored subclass of quinoline derivatives that is often difficult to access using conventional methods. The authors specifically note that classical quinoline syntheses such as the Pfitzinger and Conrad–Limpach reactions typically require multistep procedures and often suffer from low efficiency and limited substrate applicability. In contrast, the present iridium-catalyzed protocol proceeds through tandem dehydrogenation and annulation using stable 2-aminobenzyl alcohols as starting materials, thereby overcoming the instability issues associated with o-aminobenzaldehydes. The method also demonstrated good synthetic practicality. Gram-scale synthesis of 3-phenoxy-2-phenylquinoline was successfully achieved using only 2.5 mol % catalyst loading, affording the product in 65% yield. Additionally, the synthesized quinoline derivatives could undergo further derivatization, including Suzuki coupling and aldol-type condensation reactions, demonstrating their synthetic utility as valuable intermediates.

Mechanistic studies suggested that the reaction proceeds via initial iridium-catalyzed dehydrogenation of 2-aminobenzyl alcohol to generate o-aminobenzaldehyde in situ, followed by nucleophilic condensation with the phenoxy acetophenone substrate and subsequent cyclization to furnish the quinoline core. Competition experiments revealed that electron-deficient aminobenzyl alcohols undergo dehydrogenation more readily, whereas electron-rich phenoxy acetophenones react more efficiently during the annulation step.

Overall, this work provides an efficient and operationally simple route toward structurally diverse 3-oxo quinolines through iridium-catalyzed dehydrogenative annulation. The methodology combines the use of renewable lignin-derived substrates, broad functional-group compatibility, access to otherwise challenging quinoline architectures, and scalability, making it a valuable contribution to recent advances in sustainable quinoline synthesis.

**Scheme 24:**
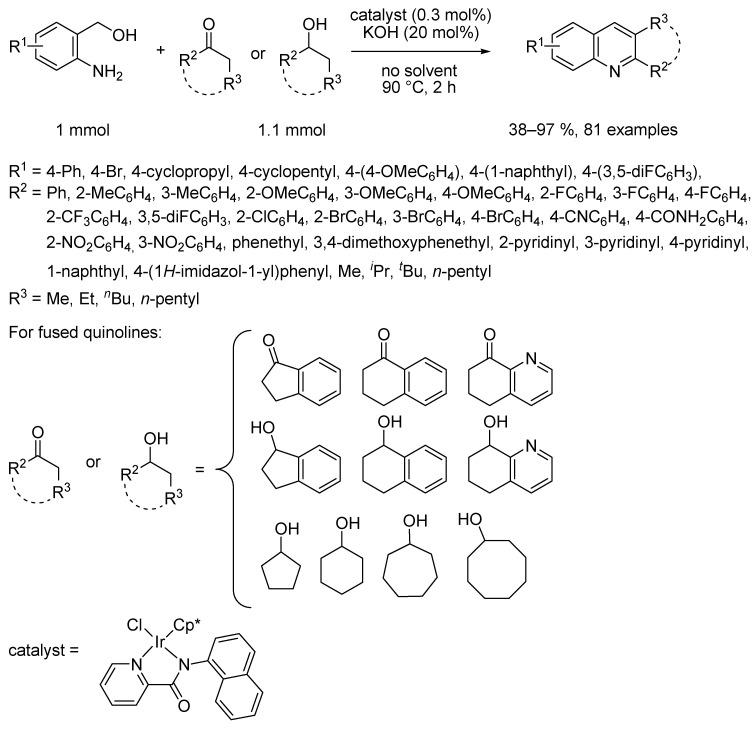
Ir(III) complex-catalyzed synthesis of quinolines.

In a 2024 study, Bakibillah and co-workers reported a family of novel Ir(III) complexes capable of efficiently catalyzing the solvent-free synthesis of quinoline derivatives from amino alcohols and ketones under relatively mild conditions [57]. The catalytic protocol enabled the formation of a broad range of quinolines in very good to excellent yields, displaying tolerance toward aromatic, heteroaromatic, and aliphatic ketones (Scheme 24).

Mechanistic and DFT studies supported a catalytic cycle initiated by Ir-mediated dehydrogenation of the amino alcohol substrate to the corresponding carbonyl intermediate, followed by condensation, cyclization, and final aromatization steps. Hydrogen liberation experiments further confirmed the operation of an acceptorless dehydrogenative coupling (ADC) pathway, in which molecular hydrogen is generated as the sole stoichiometric by-product. The high efficiency of the Ir(III) complexes was attributed to the favorable electronic properties of the ligand framework, which facilitate hydride transfer and catalyst regeneration during the catalytic cycle. Overall, this work demonstrates the effectiveness of homogeneous Ir catalysis for atom-economical quinoline synthesis under solvent-free conditions, while further emphasizing the growing relevance of hydrogen-transfer methodologies in sustainable heterocyclic synthesis.

**Scheme 25:**
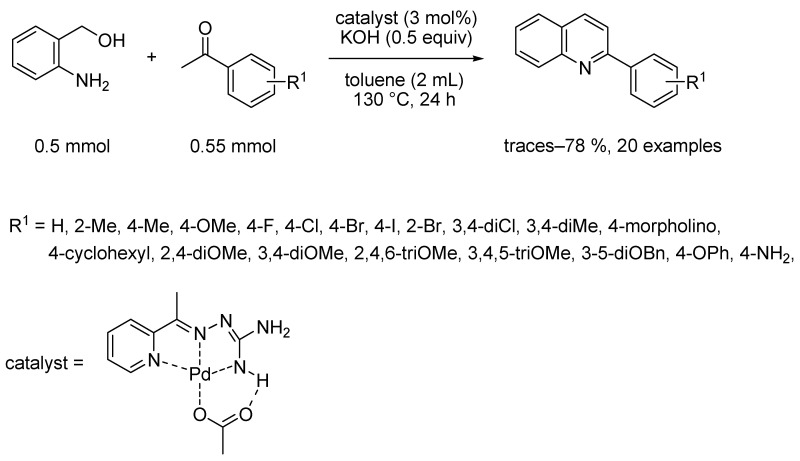
Pd(II) complex-catalyzed synthesis of quinolines.

The utility of homogeneous palladium systems for quinoline synthesis through acceptorless dehydrogenative annulation pathways has been recently reported, as well. In a 2025 study, researchers reported a family of NNN-type Pd(II) pincer complexes bearing 1,1-diaminoazine ligands as efficient catalysts for the synthesis of quinoline derivatives from *o*-aminobenzyl alcohols and ketones [58]. The catalytic methodology proceeds through borrowing-hydrogen/acceptorless dehydrogenative coupling processes under relatively mild conditions, enabling the preparation of structurally diverse quinolines from traces to good yields without requiring external oxidants or hydrogen acceptors (Scheme 25).

Mechanistic investigations suggested that the Pd(II) complexes promote the initial dehydrogenation of the amino alcohol substrate to generate the corresponding amino aldehyde intermediate, followed by condensation with the ketone partner, cyclization, and final aromatization to furnish the quinoline framework.

Despite these advantages, certain limitations remain. Many platinum-group metal-catalyzed systems still require relatively high temperatures and the presence of base additives, and, in some cases, organic solvents, which may affect overall process sustainability. Furthermore, catalyst synthesis can also involve multistep procedures, potentially reducing the overall environmental benefit. Additionally, the in situ generation of hydrogen may lead to undesired side reactions, such as over-reduction of quinoline products, requiring careful control of reaction conditions.

From a sustainability perspective, however, these methodologies compare favorably with traditional approaches. High atom economy, low catalyst loadings (down to ppm levels in some cases), and the use of readily available alcohols contribute to improved green metrics. Hence, ruthenium, iridium, or palladium-catalyzed homogeneous methodologies represent a well-developed and efficient approach for quinoline synthesis, particularly through acceptorless dehydrogenative coupling strategies. These systems combine high catalytic activity with broad substrate scope and favorable sustainability profiles. Ongoing research is expected to focus on reducing reaction temperatures, minimizing or eliminating base requirements, and expanding the use of environmentally benign solvents such as water. In addition, the development of more accessible and robust ligand frameworks will be important for enhancing the practical applicability of these catalysts. Overall, these catalytic systems are likely to remain central to future advances in sustainable quinoline synthesis.

### 2.3. Other Earth-Abundant Transition Metals

Copper-catalyzed annulation reactions have emerged as powerful and flexible tools for assembling quinoline and isoquinoline derivatives. Copper salts combine low cost, relative abundance, and comparatively low toxicity with the ability to promote diverse C-C and C-N bond-forming processes under milder conditions than many traditional protocols. As a result, copper catalysis has gained prominence as an attractive alternative to precious transition-metal systems in heterocycle synthesis [59].

**Scheme 26:**
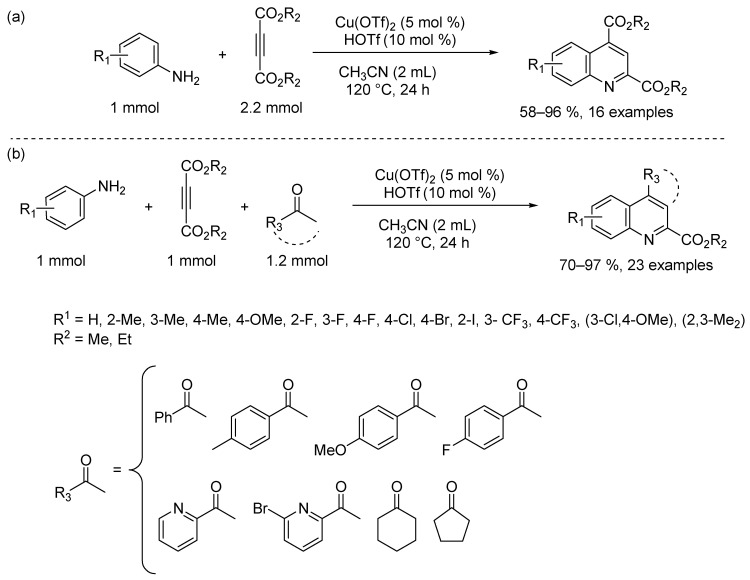
Cu(OTf)_2_-catalyzed synthesis of 2,4-disubstituted quinolines. (**a**) From aniline plus alkyne. (**b**) From aniline plus alkyne plus ketone.

Wu and co-workers reported a highly regioselective copper(II)-catalyzed cascade annulation that enables the one-pot construction of 2,4-disubstituted quinolines from simple anilines and alkyne esters [30]. The reaction tolerates a broad range of electron-donating and electron-withdrawing substituents on the aniline ring, as well as disubstituted anilines, demonstrating both electronic and steric robustness. Electron-rich anilines typically give higher yields than electron-poor analogues, but all substrates show excellent regio-control (Scheme 26a).

A key extension of the methodology is that the second alkyne equivalent can be replaced by ketones, which serve as a two-carbon synthon for the quinoline C-4 substituent. Both aryl and heteroaryl ketones (including pyridyl ketones) as well as cycloalkyl ketones are compatible, delivering structurally diverse 2,4-disubstituted quinolines (Scheme 26b). This feature greatly expands structural diversity beyond what is achievable with other classical protocols.

**Scheme 27:**
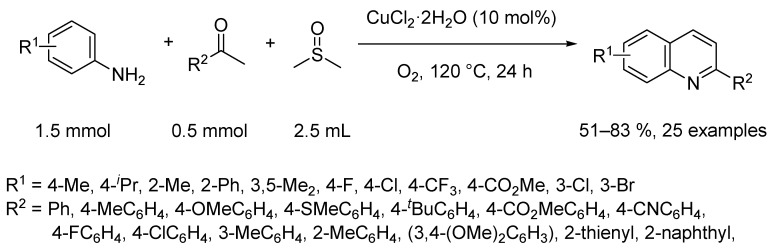
Synthesis of quinolines by Cu-catalyzed C-H cyclization.

In the same year, Liu and co-workers introduced a copper-catalyzed method for synthesizing 2-arylquinolines using molecular oxygen as an oxidant and DMSO as both solvent and carbon source. This aerobic cyclization of simple anilines and aryl ketones in DMSO tolerates a wide range of substituents, affording moderate to good yields (Scheme 27). The mechanism involves DMSO activation, leading to a series of intermediates that culminate in annulation and aromatization to form the final product [29].

**Scheme 28:**
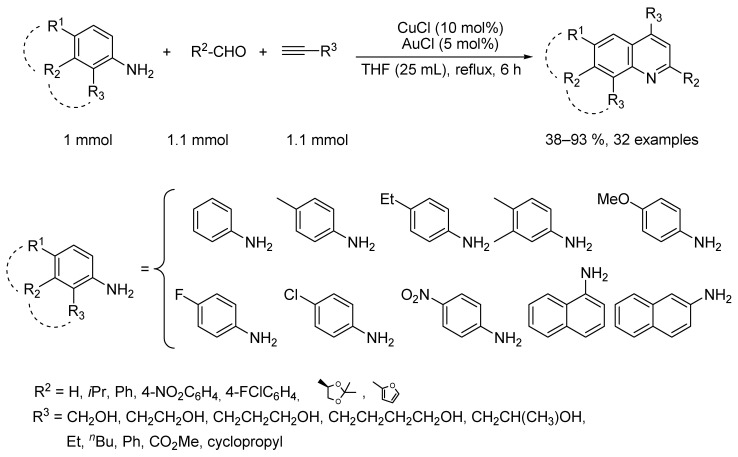
CuCl and AuCl-catalyzed coupling reaction.

Jiang and co-workers developed a dual-metal, three-component cascade strategy for the efficient synthesis of 4-hydroxyalkyl-substituted quinolines, a structurally and biologically important but synthetically underexplored class of quinoline derivatives [60]. The method combines Cu(I)- and Au(I)-catalyzed steps in a single reaction vessel to couple anilines, aldehydes, and terminal aliphatic alkynes into highly functionalized quinoline products bearing a hydroxylated alkyl chain at C-4 (Scheme 28).

The protocol exhibited a broad substrate scope. A wide variety of aromatic aldehydes and anilines bearing electron-donating or electron-withdrawing substituents (Me, OMe, F, Cl, NO_2_) furnished the corresponding 4-hydroxyalkyl quinolines in high yields. Electron-rich anilines generally performed better than electron-deficient ones, consistent with their higher nucleophilicity in imine formation. Sterically demanding substrates such as naphthylamines were also tolerated, albeit with somewhat reduced yields.

Sequential catalysis experiments unambiguously demonstrated the division of labor between Cu and Au. CuCl catalyzes the A^3^-coupling of aniline, aldehyde, and alkyne to give a propargyl amine, while AuCl is required for the subsequent cyclization and aromatization to the quinoline. When AuCl was omitted, only the propargylamine intermediate was isolated; addition of AuCl in a second step converted it efficiently into the quinoline product. Mechanistic studies and DFT calculations support a pathway in which Cu(I) first forms an alkynyl-Cu species that adds to the in situ-generated imine, yielding a propargylamine. The Au(I) catalyst then coordinates to the alkyne, triggering intramolecular cyclization via an allene–dihydroquinoline intermediate, followed by aerobic oxidation and protodeauration to deliver the aromatized 4-hydroxyalkyl quinoline.

Recent reviews on metal-catalyzed quinoline synthesis have confirmed the continued relevance of Cu-catalyzed methodologies, particularly for cascade annulation and oxidative coupling processes, although comparatively fewer fundamentally new homogeneous Cu systems have been reported in recent years relative to rapidly expanding borrowing-hydrogen strategies based on Ru, Ir, and Fe catalysts [61,62].

**Scheme 29:**
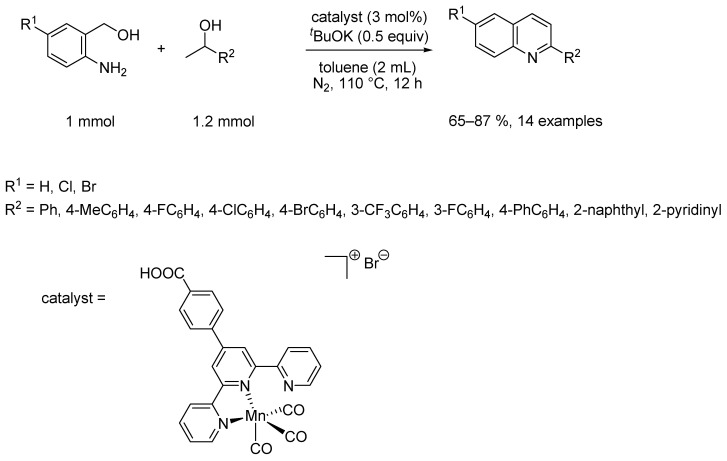
Synthesis of Quinolines Catalyzed by a Mn(I) Complex.

In a 2024 study, researchers reported a homogeneous manganese-catalyzed methodology for the synthesis of quinoline derivatives through acceptorless dehydrogenative coupling (ADC) reactions involving 2-aminobenzyl alcohols and ketones [63]. Using a well-defined manganese pincer complex as a catalyst, the transformation proceeds through sequential alcohol dehydrogenation, condensation, cyclization, and aromatization steps, enabling the efficient construction of quinoline frameworks under relatively mild conditions. The catalytic system exhibited broad substrate scope and tolerated a variety of aromatic and aliphatic ketones bearing electron-donating and electron-withdrawing substituents, affording the desired quinolines in generally good to excellent yields. Importantly, the methodology generates molecular hydrogen as the sole stoichiometric by-product, thereby enhancing atom economy and sustainability (Scheme 29).

Mechanistic investigations suggested the involvement of manganese-hydride intermediates and metal–ligand cooperative processes characteristic of borrowing-hydrogen catalysis. This work further illustrates the growing potential of earth-abundant first-row transition metals beyond iron, cobalt, and nickel in sustainable quinoline synthesis and highlights the rapid expansion of manganese-based hydrogen-transfer catalysis as an attractive alternative to noble-metal systems.

**Scheme 30:**
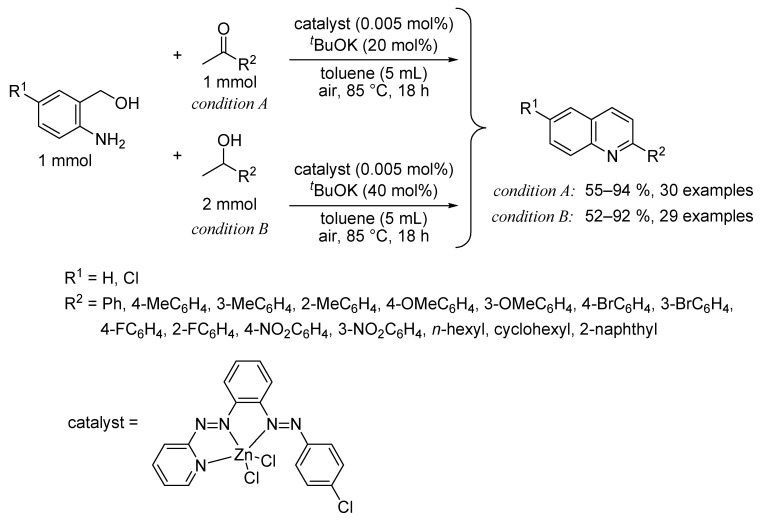
Zinc-catalyzed homogeneous Friedländer quinoline synthesis.

In another study, researchers reported a homogeneous zinc-catalyzed methodology for the synthesis of quinoline derivatives through tandem condensation–cyclization reactions under relatively mild and environmentally benign conditions [64]. The catalytic system employed was an air- and moisture-stable, homogeneous zinc catalyst, stabilized using an electron-deficient NNN pincer-type ligand, molecular capable of efficiently promoting the annulation of 2-aminoaryl carbonyl compounds with activated ketones or related coupling partners, affording a broad range of substituted quinolines in moderate to excellent yields. The methodology exhibited good functional-group tolerance toward both electron-rich and electron-deficient aromatic substrates and proceeded with operational simplicity under comparatively mild reaction conditions (Scheme 30).

Mechanistic investigations suggested that the Zn catalyst acts primarily as a Lewis acid, activating carbonyl groups and facilitating imine formation, cyclization, and subsequent aromatization steps leading to the quinoline framework. Importantly, the use of zinc, an abundant, inexpensive, and relatively low-toxicity metal, highlights the growing interest in replacing noble-metal catalysts with more sustainable homogeneous catalytic systems for heterocycle synthesis. This work further demonstrates that, beyond hydrogen-transfer methodologies dominated by Fe-group and platinum-group metals, Lewis-acid-mediated homogeneous catalysis with main-group transition metals remains a valuable strategy for the development of greener quinoline syntheses.

**Scheme 31:**
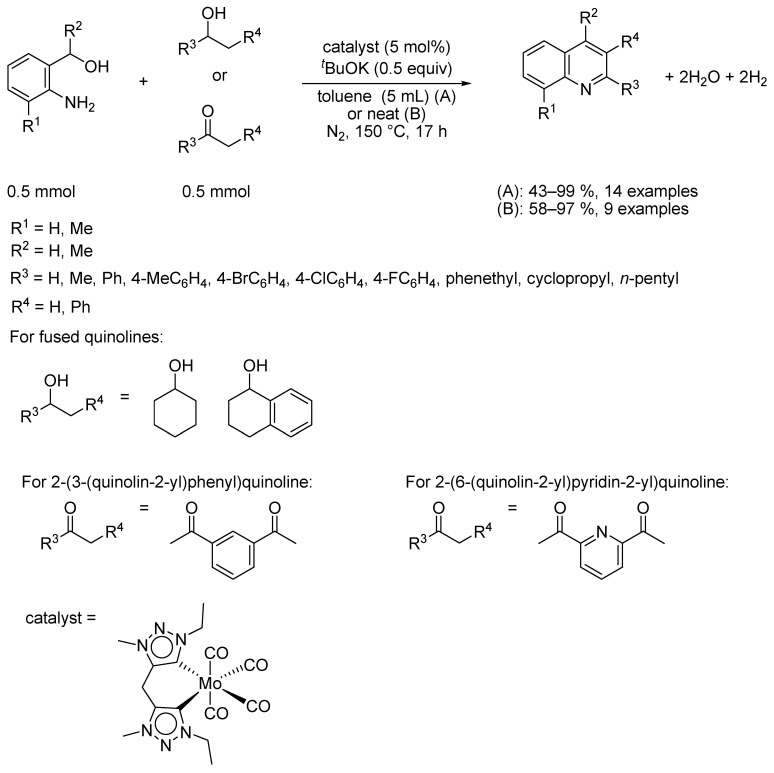
Molybdenum-catalyzed synthesis of quinolines.

Also in 2024, the first molybdenum triazolylidene complex catalyzed synthesis of quinolines through acceptorless dehydrogenative coupling of alcohols was reported [65]. The catalytic system enabled the efficient annulation of 2-aminobenzyl alcohols with ketones via tandem alcohol dehydrogenation, condensation, cyclization, and aromatization steps, affording a broad range of quinoline derivatives in moderate to excellent yields. The methodology proceeded under relatively mild conditions and generated molecular hydrogen as the sole stoichiometric by-product, thereby enhancing atom economy and sustainability (Scheme 31).

Mechanistic studies suggested that the catalytic transformation involves metal–ligand cooperative hydrogen-transfer processes characteristic of borrowing-hydrogen catalysis, with the triazolylidene ligand framework playing a crucial role in stabilizing reactive molybdenum intermediates during the catalytic cycle. Importantly, this work demonstrates the growing potential of non-noble early transition metals in homogeneous hydrogen-transfer catalysis and expands the scope of sustainable quinoline synthesis beyond the more commonly explored Fe-, Co-, Ni-, and platinum-group-metal catalytic systems.

Overall, recent developments involving other earth-abundant transition metal catalysts clearly demonstrate the expanding diversity of homogeneous catalytic strategies available for sustainable quinoline synthesis. While Cu- and Zn-based systems continue to highlight the importance of Lewis-acid-mediated annulation and oxidative cyclization processes, more recent Mn- and Mo-catalyzed methodologies illustrate the rapid emergence of borrowing-hydrogen and acceptorless dehydrogenative coupling (ADC) paradigms employing earth-abundant transition metals. These advances collectively reflect a broader shift in modern quinoline synthesis toward atom-economical tandem transformations that minimize waste generation and avoid the use of stoichiometric oxidants. Moreover, the increasing implementation of pincer-type and metal–ligand cooperative catalytic systems beyond traditional noble-metal chemistry underscores the growing convergence between sustainability, mechanistic sophistication, and catalyst design in the development of next-generation quinoline synthetic methodologies.

### 2.4. Main-Group Metals

In addition to classical transition metals, certain post-transition metals have demonstrated notable performance. For example, indium-based catalysts exhibit high activity in quinoline synthesis under both solvent and solvent-free conditions, highlighting the versatility of main-group metal catalysis in this field [33].

The use of indium trichloride (InCl_3_) as a catalyst in the synthesis of a broad range of heterocyclic compounds has attracted considerable attention in recent years. InCl_3_ is a water-tolerant Lewis acid of relatively low toxicity that enables quinoline formation without the need for strong Brønsted acids, stoichiometric oxidants, or precious-metal catalysts. Moreover, the in situ generation of organoindium species from indium (III) salts and organic substrates has largely obviated the use of sensitive and toxic organometallic reagents. Consequently, numerous InCl_3_-catalyzed transformations have been reported in the literature, underscoring the emergence of InCl_3_ as a versatile and valuable catalyst for a wide range of organic reactions, particularly owing to its stability under moist conditions and in aqueous media [33].

Chanda et al. investigated the use of indium(III) chloride as a catalyst for quinoline synthesis [66]. Although this work falls slightly outside the temporal scope of the present review, defined broadly as the past decade, it has been deliberately included due to its seminal and comprehensive contribution to the development of InCl_3_-catalyzed quinoline synthesis. In this study, the authors established a unified and versatile catalytic platform capable of enabling multiple quinoline-forming pathways under mild reaction conditions. Notably, several transformations could be performed under solvent-free conditions, thereby anticipating key concepts that later became central to green and sustainable heterocyclic synthesis. The methodology demonstrates broad substrate scope, high efficiency, and significant generality, and it has exerted a lasting influence on subsequent strategies for quinoline construction. For these reasons, it is appropriately considered a foundational reference within this field.

**Scheme 32:**
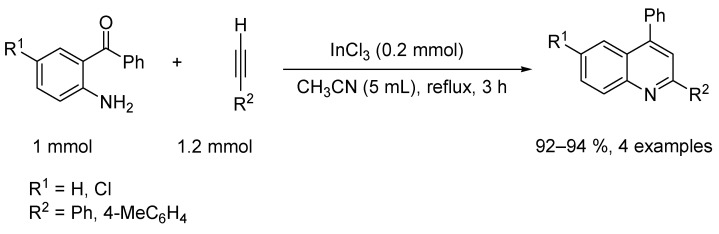
Reaction of 2-aminoarylketones and alkynes catalyzed by InCl_3_.

A variety of reaction media were examined, including acetonitrile, ethanol, toluene, and tetrahydrofuran, as well as solvent-free conditions; the results obtained under solvent-free conditions are discussed separately in the Section 6.2 of this review. Several catalysts, including different metal halides, were also evaluated. Optimal performance, defined by the highest product yields, the cleanest reaction profiles, and the simplest work-up procedures, was achieved using 20 mol% InCl_3_ in acetonitrile (Scheme 32).

**Scheme 33:**
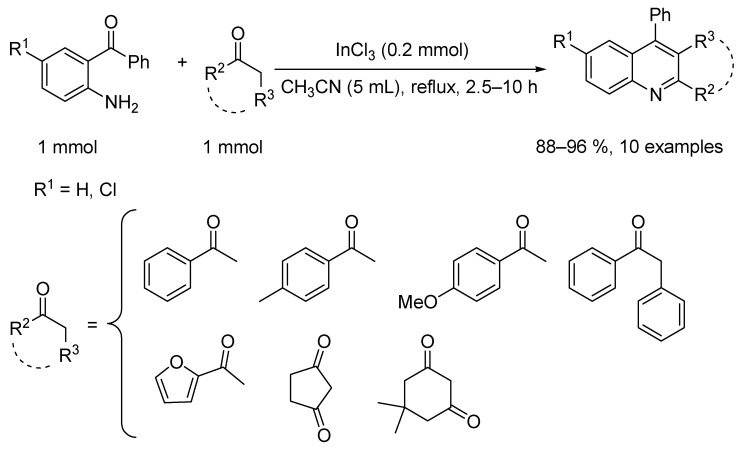
InCl_3_-catalyzed Friedländer reaction.

The scope of this InCl_3_-catalyzed annulation was further evaluated by performing the Friedländer reaction between 2-aminoaryl ketones and cyclic/acyclic active methylene compounds (Scheme 33).

**Scheme 34:**
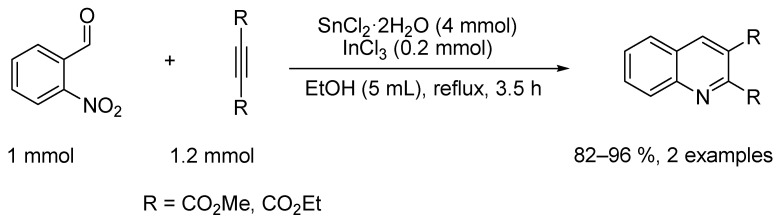
Reaction of 2-nitrobenzaldehyde and alkynes catalyzed by InCl_3_.

Furthermore, to expand the scope of this synthesis and to gain a greater insight into the reaction, the reaction of 2-nitrobenzaldehyde with symmetrical alkynes in the presence of SnCl_2_·2H_2_O and InCl_3_ in ethanol was also performed. The nitro compound is reduced by the tin (II) chloride, and the expected quinoline is obtained in good yield (Scheme 34).

Therefore, indium (III) chloride is established as an efficient and versatile catalyst for the Friedländer annulation under mild conditions. Its use avoids strong acids or bases, external oxidants, and co-catalysts, while providing high regioselectivity and excellent yields across a broad substrate scope.

**Scheme 35:**
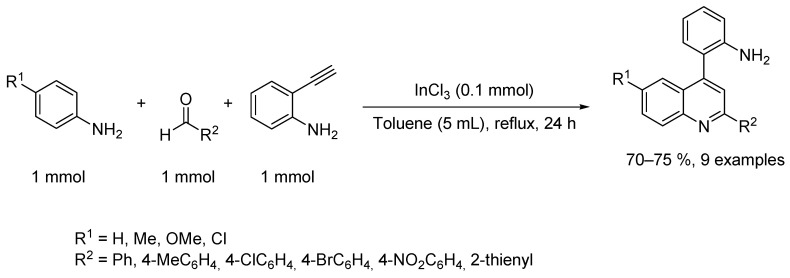
Povarov synthesis of quinolines catalyzed by InCl_3_.

Indium(III) chloride has also been employed by Singh and co-workers as a catalyst in an aza-Diels–Alder (Povarov) reaction, providing an efficient strategy for the synthesis of highly functionalized 2,4-disubstituted quinolines [67]. In this study, particular emphasis was placed on achieving mild reaction conditions while maintaining high catalytic efficiency, further highlighting the versatility of InCl_3_ as a Lewis acid catalyst for quinoline construction (Scheme 35).

Although many seminal indium-catalyzed quinoline syntheses were reported during the 2000s and early 2010s, more recent reviews continue to recognize indium catalysis as a valuable platform for heterocycle synthesis owing to the mild Lewis acidity, moisture tolerance, functional-group compatibility, and operational simplicity of In(III) catalysts [33].

**Scheme 36:**
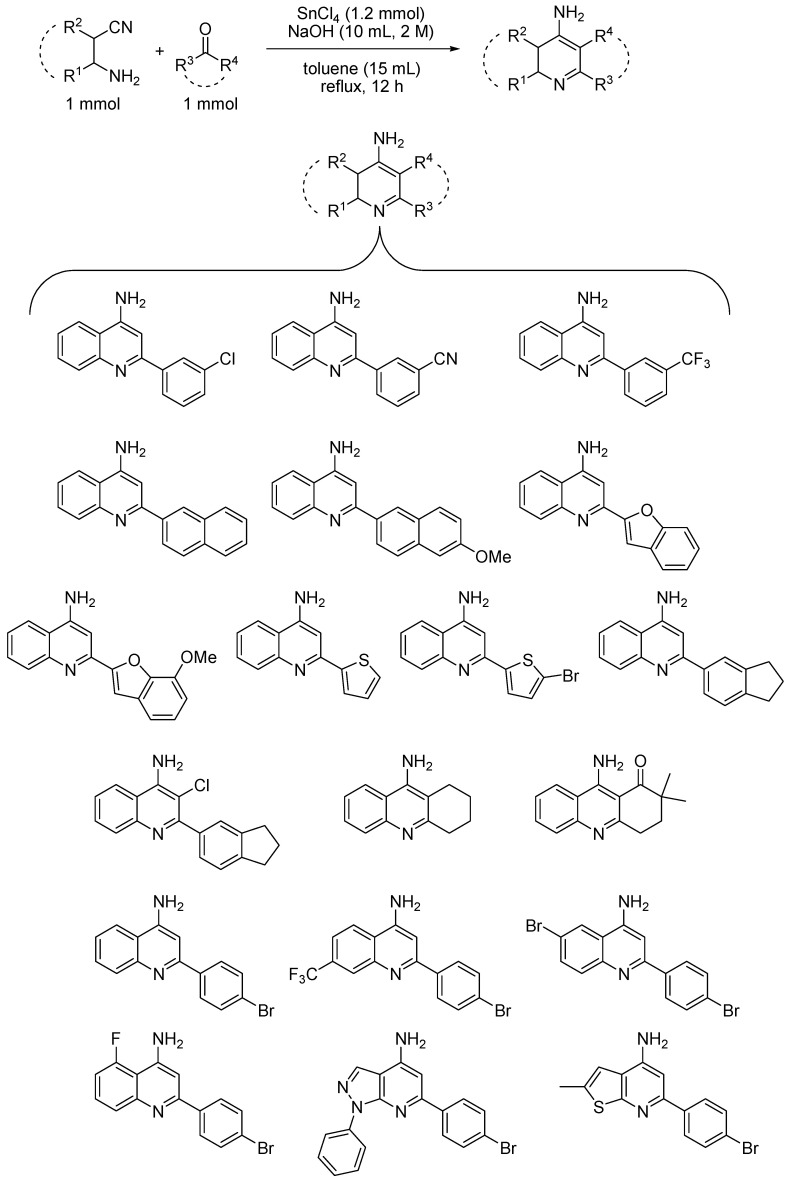
SnCl_4_-mediated synthesis of 4-aminoquinoline derivatives.

Chirra et al. developed a one-pot Lewis acid-mediated Friedländer-type annulation for the synthesis of 2-substituted 4-aminoquinolines from readily available anthranilonitriles and methyl ketones, delivering a broad range of derivatives in good to excellent yields [68]. The method relies on SnCl_4_-promoted carbonyl activation and intramolecular cyclization, avoids transition-metal catalysis, and offers notable step economy and substrate generality compared with Pd- or Cu-based protocols (Scheme 36).

From a green-chemistry perspective, the approach benefits from operational simplicity and the use of inexpensive starting materials; however, the requirement for stoichiometric SnCl_4_ and refluxing toluene represents a limitation when benchmarked against greener Lewis acids such as InCl_3_ or ZnCl_2_, which are typically effective at catalytic loadings, display lower toxicity, and often enable solvent-free or milder reaction conditions in related quinoline syntheses. Nevertheless, the methodology remains synthetically valuable, particularly for the efficient construction of biologically relevant 4-aminoquinoline scaffolds, and highlights both the potential and the challenges of Lewis-acid-driven annulation strategies in the context of sustainable quinoline synthesis.

Overall, the studies discussed in this section reveal several recurring mechanistic paradigms that currently dominate homogeneous metal-catalyzed quinoline synthesis. Classical Lewis-acid-mediated processes, particularly those involving Cu, In, Sn, and Zn catalysts, generally proceed through activation of carbonyl substrates followed by condensation, cyclization, and final aromatization steps. In contrast, more recent methodologies based on Fe-, Co-, Ni-, Mn-, Mo-, Ru-, Ir-, and Pd-catalyzed systems increasingly rely on borrowing-hydrogen or acceptorless dehydrogenative coupling (ADC) pathways, in which alcohol substrates are first dehydrogenated in situ to generate reactive carbonyl intermediates that subsequently undergo annulation. These tandem catalytic processes are especially attractive from a sustainability perspective because they improve atom economy and frequently generate molecular hydrogen as the sole stoichiometric by-product.

Another major trend emerging from recent literature is the growing importance of metal–ligand cooperative catalysis. In many modern pincer and pincer-like catalytic systems, the ligand framework actively participates in hydrogen-transfer events, proton shuttling, or redox processes, rather than functioning solely as a spectator ligand. Such cooperative effects have enabled earth-abundant first-row transition metals to emulate mechanistic pathways traditionally associated with platinum-group-metal catalysis. Furthermore, several homogeneous catalytic systems combine multiple activation modes, including oxidative annulation, radical pathways, Lewis-acid activation, and hydrogen-transfer catalysis, highlighting the increasing mechanistic sophistication of contemporary quinoline synthesis. Collectively, these developments demonstrate how advances in catalyst design, ligand engineering, and tandem catalytic strategies are reshaping homogeneous quinoline synthesis toward more sustainable, selective, and atom-economical methodologies.

## 3. Non-Metal Catalyzed Approaches

Extensive efforts have been made in this area towards the development of multicomponent reactions for construction of heterocycles [69]. The shift towards non-metal catalysis represents a significant advancement in the field of green chemistry, driven by the demand for more sustainable and environmentally conscious synthetic methodologies. Unlike traditional metal-based catalysts, organocatalysts operate efficiently under mild conditions, often without the need for inert atmospheres or anhydrous solvents. This not only reduces energy consumption but also minimizes environmental hazards and waste generation [10,18,70]. Non-metal catalysis has emerged as a compelling alternative in the synthesis of quinoline derivatives, particularly due to their low toxicity, affordability, and ease of handling. Their stability in the presence of moisture and air makes them highly attractive for large-scale applications, especially in the pharmaceutical industry, where metal contamination must be strictly avoided. Moreover, organocatalytic systems often exhibit high chemo-, regio-, and stereoselectivity, which further enhances their utility in complex molecule construction. These attributes strongly align with the principles of green chemistry and offer a promising route toward more sustainable drug development and material synthesis [20,71].

### 3.1. Iodine-Catalyzed Reactions

Iodine is one of the heaviest non-radioactive elements, and it is the largest, least electronegative, and most polarizable of the halogens in the periodic table, having high significance in chemistry as well as biology [72]. Iodine and its derivatives have proven to be highly effective and eco-friendly reagents in organic synthesis. A particularly notable advancement in this field is the recognition of iodine’s catalytic role in a variety of oxidative reactions that facilitate the construction of C-O, C-N, and C-C bonds [73]. Iodine compounds have also been adopted in several industrial contexts, including catalysis, polymers, liquid crystal displays, food processing, healthcare, and pharmaceuticals. Approximately 16% of global iodine production is employed in catalytic processes.

**Scheme 37:**
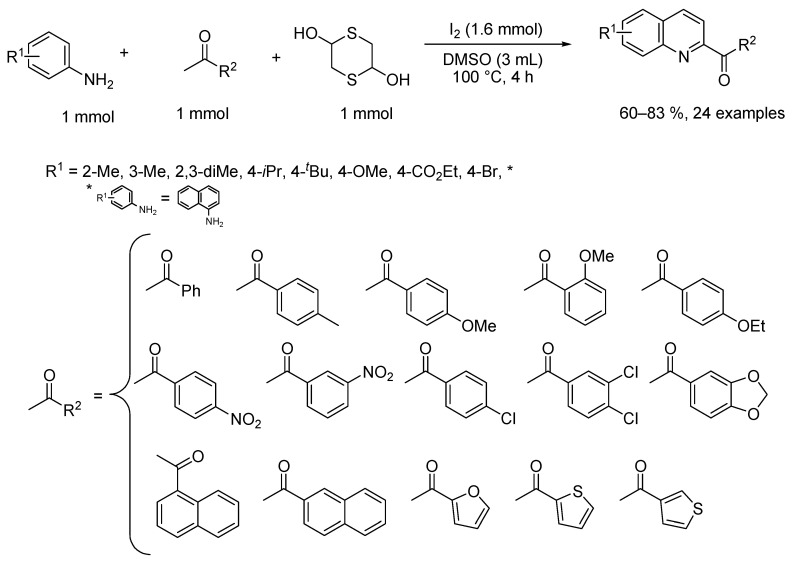
Iodine-mediated synthesis of quinoline involving 1,4-dithiane.

In a 2017 study, Wu et al. reported an iodine-catalyzed version of the Povarov reaction, a highly efficient method for synthesizing 2-acylquinolines through a [4+2] cycloaddition reaction from methyl ketones and arylamines [74]. They utilized I_2_ as a catalyst and employed 1,4-dithiane-2,5-diol as an ethylene surrogate (Scheme 37).

**Scheme 38:**
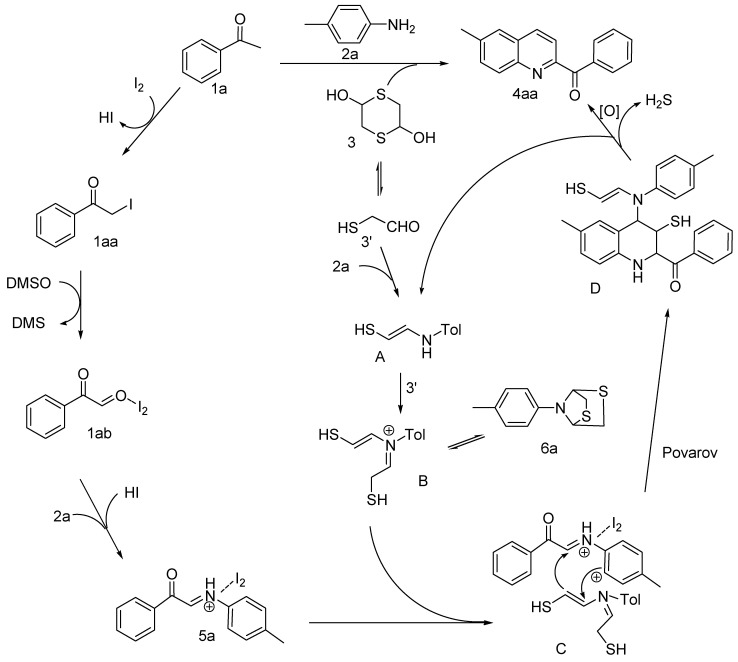
Mechanism for the synthesis of quinoline involving 1,4-dithiane.

In the proposed reaction mechanism (Scheme 38), acetophenone (1a) is transformed into phenylglyoxal 1ab, releasing HI and DMS via an iodination/Kornblum oxidation sequence. Intermediate 1ab then undergoes a dehydration reaction with *p*-toluidine (2a) to form the iminium ion 5a. The mercaptoacetaldehyde generated from 1,4-dithiane-2,5-diol under equilibrium conditions is activated by *p*-toluidine (2a) to form enamine intermediate B, which reacts with 5a to generate intermediate D. Intermediate D then undergoes an oxidative aromatization to yield the desired product 4aa via desulfurization and deamination steps.

### 3.2. Acid-Catalyzed Reactions

Acids have long served as simple, cost-effective, and efficient catalysts and promoters in a wide range of organic transformations. Commonly employed acid catalysts in organic synthesis include trifluoroacetic acid (TFA), formic acid, acetic acid, triflic acid, and *para*-toluenesulfonic acid (*p*-TSA). Among these, acetic acid and formic acid are especially popular due to their low cost, availability, and well-documented utility in the synthesis of quinoline derivatives [69,75]. Arylamines are among the most frequently utilized starting materials for quinoline construction, owing to their nucleophilic nature and structural versatility. Numerous acid-catalyzed protocols rely on classical name reactions, with the Povarov reaction being one of the most extensively studied strategies for quinoline synthesis. This reaction, which proceeds through an aza-Diels–Alder cycloaddition, represents a powerful approach for constructing the quinoline scaffold in a single step [22].

**Scheme 39:**
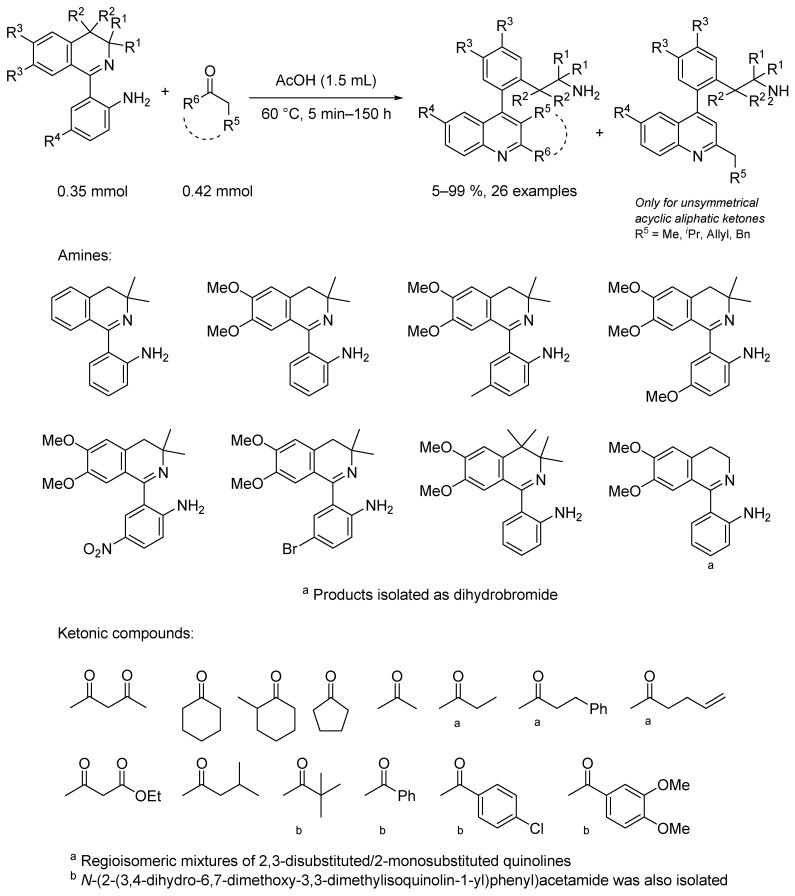
Synthesis of quinolines from 2-(3,4-dihydroisoquinolin-1-yl)anilines.

Rozhkova and co-workers reported a mild and efficient Friedländer-type annulation for the synthesis of aminoalkyl-functionalized 4-arylquinolines, based on the condensation of 2-(3,4-dihydroisoquinolin-1-yl)anilines with α-methylene ketones in acetic acid [76]. The reaction proceeds at relatively low temperature (60 °C) and generally provides the target quinolines in moderate to excellent yields (30–99%) over, typically, short reaction times, demonstrating broad tolerance toward aryl and heteroaryl ketones as well as diverse substituents on the aniline precursor (Scheme 39). From a synthetic design perspective, the method exploits cyclic Schiff bases as masked 2-aminobenzylamine equivalents, thereby extending the scope of Friedländer chemistry beyond classical 2-aminobenzophenone or 2-aminoaryl ketone substrates.

**Scheme 40:**
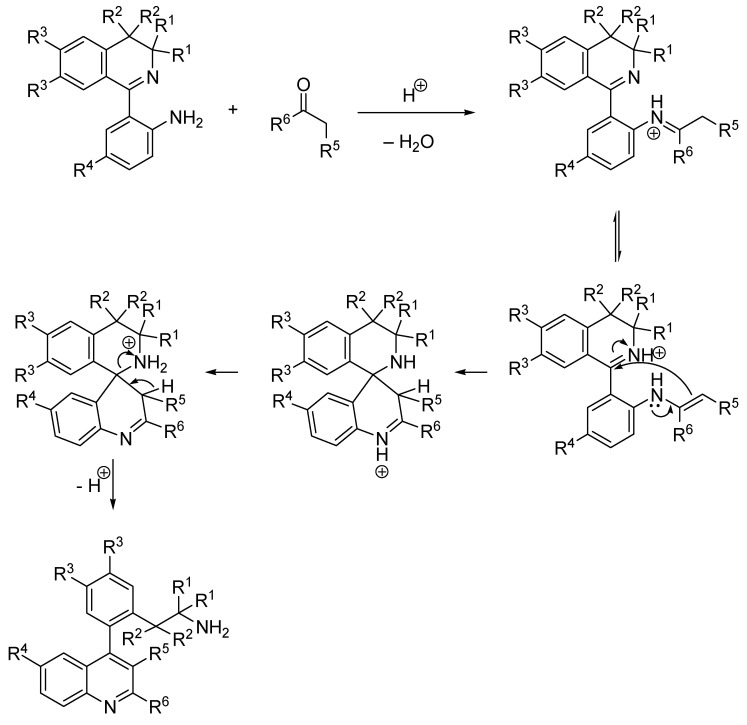
Mechanism of the Friedländer-type annulation.

Mechanistically, the process involves acid-promoted enamine formation from the α-methylene ketone followed by intramolecular cyclization onto the iminium/nitrilium center of the dihydroisoquinoline moiety, generating a transient spirocyclic intermediate that undergoes ring opening to furnish the quinoline core with a tethered aminoalkyl substituent at C-4. The use of acetic acid as both solvent and promoter, combined with the absence of metal catalysts, strong mineral acids, or prefunctionalized electrophiles, aligns the protocol with several green-chemistry principles, including safer reaction media, step economy, and structural diversification in a single operation (Scheme 40).

**Scheme 41:**
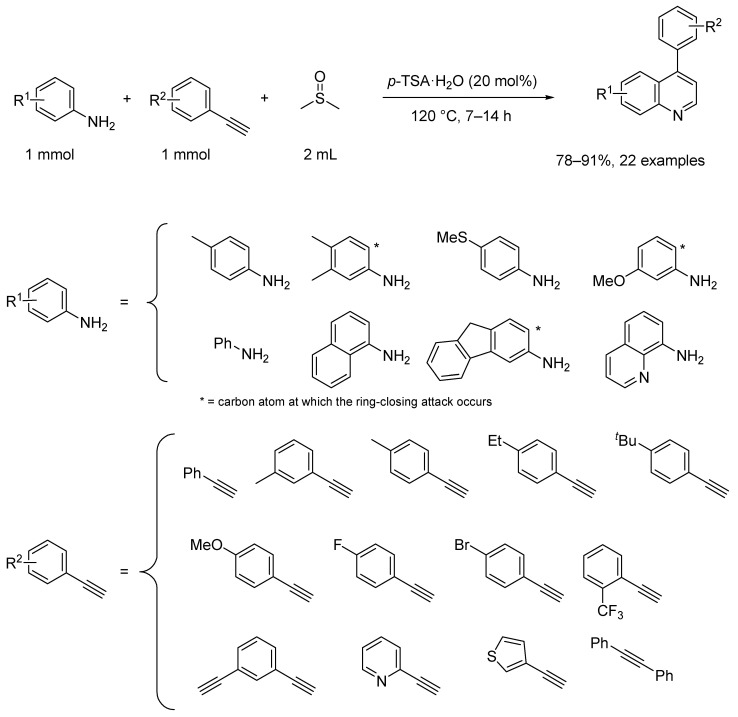
*p*-TSA·H_2_O-catalyzed synthesis of 4-aryl quinolines.

Building on their solvent-free *p*-TSA-driven quinoline chemistry, whose details can be found in the Section 6.2 of this review, Faraz and Kahn developed a conceptually more advanced three-component strategy in which DMSO acts simultaneously as solvent and C_2_ synthon, enabling the synthesis of 4-aryl quinolines from only arylamines and arylacetylenes [71]. The scope with respect to aryl acetylenes was very broad, tolerating alkyl, alkoxy, halogen, CF_3_, ethynyl, heteroaryl (pyridyl, thienyl), and even 1,2-diphenylacetylene (Scheme 41). The variation in arylamines showed that aniline, mono-substituted and polycyclic anilines, and even 8-aminoquinoline reacted smoothly, affording structurally diverse 4-aryl quinolines. However, sterically hindered (3,5-dimethyl, 3,5-dimethoxy, 3,4,5-trimethoxy) or electron-poor anilines (F, Cl, Br, NO_2_) failed, revealing mechanistic sensitivity to nucleophilicity and sterics. Other acids (MsOH, TfOH, CSA) were markedly less effective, again confirming the unique role of *p*-TSA·H_2_O.

**Scheme 42:**
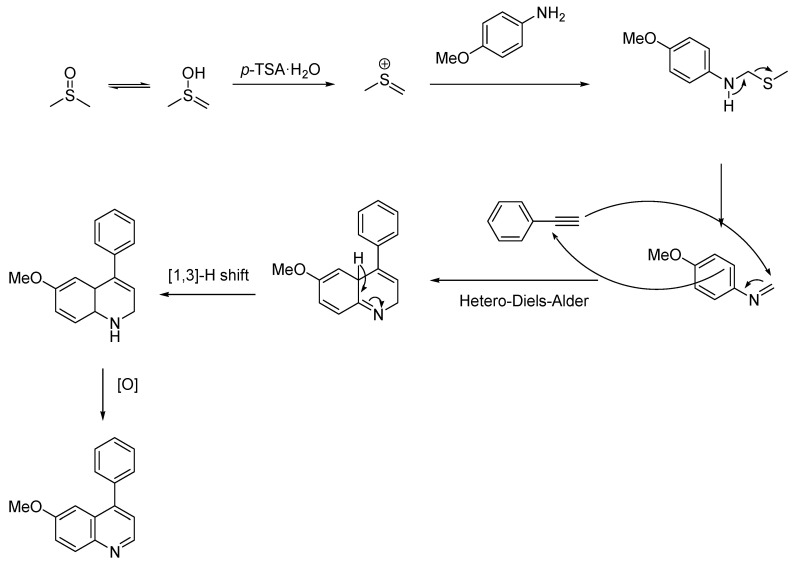
Proposed mechanism for the *p*-TSA·H_2_O-catalyzed synthesis of 4-aryl quinolines.

Mechanistically (Scheme 42), *p*-TSA·H_2_O converts DMSO into a reactive sulfenium ion, which reacts with aniline to form an iminium species. This intermediate undergoes a hetero-Diels–Alder (Povarov-type) reaction with arylacetylene, followed by [1,3]-H shift and aerobic oxidation, inserting a methylene unit derived from DMSO into the quinoline core to generate the 4-aryl quinoline. This approach achieves two C-C and one C-N bond formations plus CH_2_ insertion in a single metal-free operation, representing one of the most atom-economical DMSO-based quinoline syntheses reported.

**Scheme 43:**
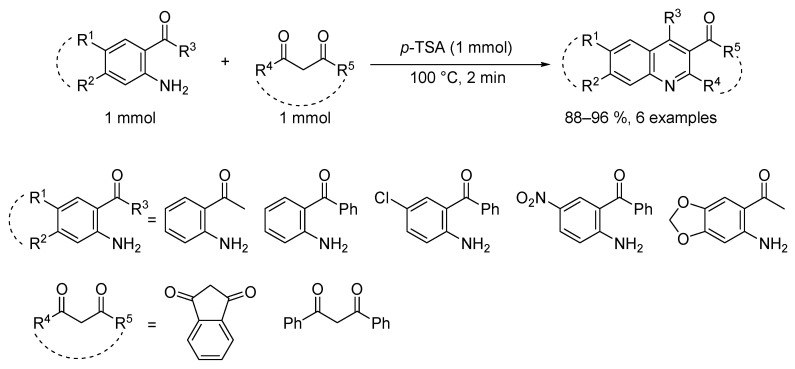
*p*-*TSA*-catalyzed Friedländer synthesis of quinolines.

George and Kannadasan employed a *p*-TSA catalyzed synthesis of 11*H*-indeno[1,2-*b*]quinolin-11-one derivatives as a first step to obtain 11-(phenylethynyl)-11*H*-indeno[1,2-*b*]quinolin-11-ol [77]. The quinoline derivatives were obtained by reacting equimolar amounts of 2-aminoarylketone and 1,3-dione in the presence of 1.0 equiv of *p*-TSA under neat conditions at 100 °C for only 2 min, affording the products in excellent isolated yields. The reaction mixture was simply cooled, treated with water, neutralized with sodium bicarbonate, and the solid product isolated by filtration, avoiding chromatographic purification at this stage. This very short reaction time, solvent-free protocol, and straightforward work-up make the method attractive from a sustainability standpoint. 2-Aminoacetophenone derivatives generally provided higher yields than the corresponding benzophenone analogues, and electronic effects were significant: electron-donating substituents (e.g., methylenedioxy) enhanced the yields, whereas electron-withdrawing groups (e.g., nitro) led to diminished conversions (Scheme 43).

Overall, despite the diversity of catalyst-free and non-metal-catalyzed quinoline syntheses reported in recent years, several common mechanistic features can be identified. Most methodologies rely on tandem condensation–cyclization–aromatization sequences initiated by the formation of imine, enamine, or related activated intermediates generated in situ from aminoaryl carbonyl compounds and carbonyl partners. In many cases, Brønsted acid activation, hydrogen-bonding interactions, or the intrinsic reactivity of activated methylene compounds facilitate cyclization without the need for transition-metal catalysts. Multicomponent domino processes are especially common, enabling the rapid assembly of quinoline frameworks through sequential C-C and C-N bond-forming events in a single operation. Collectively, these studies demonstrate that efficient quinoline synthesis can frequently be achieved through carefully designed substrate activation and cascade reactivity, even in the absence of metal catalysts, thereby contributing to the development of greener and operationally simpler synthetic methodologies.

## 4. Heterogeneous and Nanostructured Catalysts

This section covers heterogeneous and nanostructured catalytic systems, which operate in a distinct phase from the reaction medium. Photochemically active heterogeneous systems are discussed separately in the Section 5. Nanocatalysts are defined as catalytic materials possessing at least one dimension within the nanometer scale (1–100 nm), often exhibiting distinctive properties such as high surface area and size-dependent reactivity. Heterogeneous catalysts are characterized by operating in a phase distinct from that of the reactants, with nanocatalysts constituting a subclass of such systems when employed in solid form. In contrast to the homogeneous metal catalysts discussed above, these systems offer facile separation and recyclability.

Over the past decade, nanomaterials and nanocatalysts have emerged as highly effective alternatives to conventional materials across a broad spectrum of catalytic organic reactions. Various types of nanocatalysts have been explored, including nanomixed metal oxides, graphene-based systems, magnetic nanocatalysts, nanosupported catalysts, and core–shell structures, all of which have shown promising catalytic performance [78,79,80]. Nanocatalysts offer numerous advantages, such as exceptional specificity due to their unique properties, including high surface area, discrete energy state densities, and plasmonic behavior. They also exhibit high activity, excellent reusability, cost-effective synthesis, remarkable selectivity, mechanical robustness, thermal resistance, and outstanding overall stability. Consequently, the advancement of novel, sustainable catalytic methodologies not only minimizes environmental impacts associated with chemical processes but also reduces operational costs related to energy consumption and waste management [8,81].

### 4.1. Nanostructured Catalysts

The use of nanocatalysts and nanocomposites has emerged as a promising and sustainable approach for the development of greener methodologies in quinoline synthesis, often providing enhanced efficiency, selectivity, and catalyst recyclability [1,8,82]. Among the various systems explored, iron-based nanostructured catalysts have attracted particular attention in the pursuit of highly efficient and environmentally benign catalytic processes that minimize costs, waste generation, and energy consumption [83,84]. The appeal of iron derives from its natural abundance, low cost, low toxicity, and biocompatibility, making it an attractive alternative to less sustainable transition metals in catalytic applications [85,86]. Reflecting this growing interest, several recent reviews have highlighted the expanding role of iron-based heterogeneous systems in quinoline synthesis, particularly magnetic nanoparticles, supported catalysts, and recyclable nanostructured materials developed over the past decade [61,87,88].

**Scheme 44:**
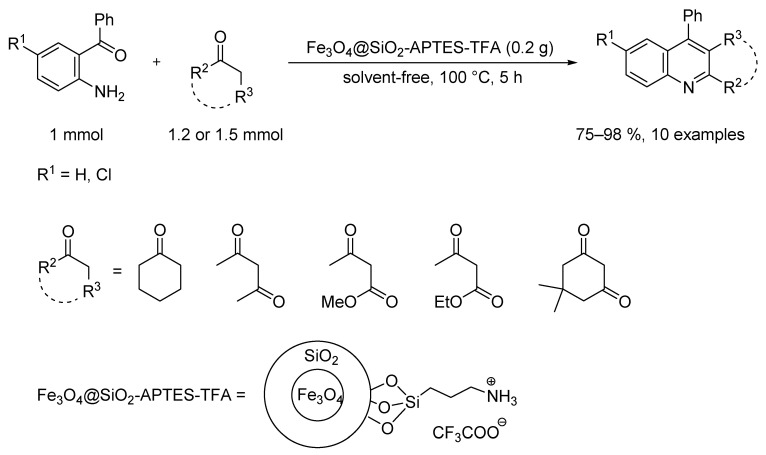
Friedländer quinoline synthesis with Fe_3_O_4_@SiO_2_-APTES-TFA as a catalyst.

Jafarzadeh and colleagues developed a magnetically recoverable core–shell nanocatalyst, Fe_3_O_4_@SiO_2_-APTES-TFA, functionalized with trifluoroacetic acid [89]. The heterogeneous nanocatalyst was thoroughly characterized by techniques such as FTIR, XRD, TGA, CHN analysis, TEM, and DLS. In the Fe_3_O_4_@SiO_2_-CH_2_CH_2_CH_2_NH_3_+CO_2_CF_3_ structure, strong ionic interactions between quaternary ammonium and trifluoroacetate groups result in the formation of ion pairs with properties similar to those of ionic liquids. The agglomerated nanocatalyst features core sizes averaging below 50 nm. This material was effectively applied in a solvent-free Friedländer annulation reaction for synthesizing quinoline derivatives, using various cyclic and acyclic 1,3-dicarbonyl compounds. Under solvent-free conditions at 100 °C and in the presence of only 0.2 g of catalyst, high yields of quinolines were obtained when 1 mmol of the 2-aminobenzophenone derivatives were reacted with either 1.2 or 1.5 mmol of the carbonyl compound. Remarkably, the yields were comparable when using either aromatic or aliphatic ketones. Additionally, the catalyst could be easily recovered using an external magnet and reused for at least four cycles without significant loss of catalytic performance (Scheme 44).

**Scheme 45:**
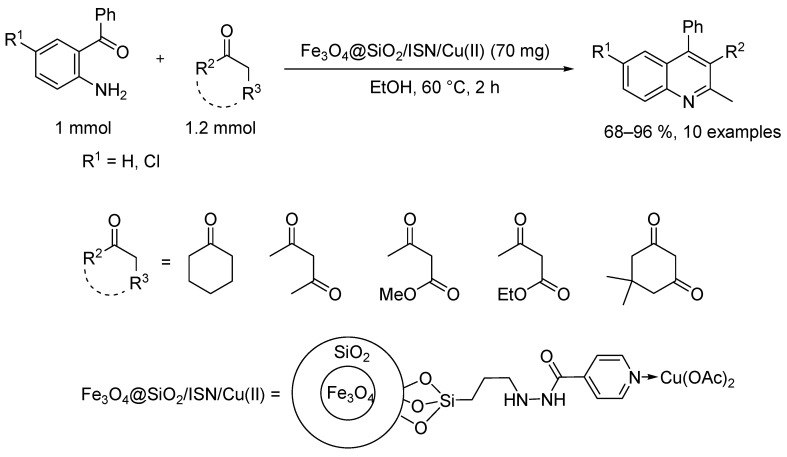
Friedländer quinoline synthesis with Fe_3_O_4_@SiO_2_/ISN/Cu(II) as a catalyst.

Lotfi and co-workers synthesized a magnetically recoverable nanocatalyst, Fe_3_O_4_@SiO_2_/isoniazid/Cu(II), characterized by various techniques such as TEM, SEM, XRD, TGA, VSM, EDS, and FT-IR [90]. The spherical nanoparticles, averaging 20–30 nm, were formed on amino-functionalized Fe_3_O_4_ cores. This catalyst efficiently promoted the Friedländer synthesis of quinoline derivatives from α-methylene ketones and 2-aminoaryl ketones in ethanol at 60 °C, yielding 68–96% within 2 h using 70 mg of catalyst. Although cyclohexanone gave lower yields, substituents on the amino aryl ketones had minimal effect. The catalyst could be reused up to four times with moderate activity loss (~24%), offering easy magnetic recovery and good stability (Scheme 45).

**Scheme 46:**
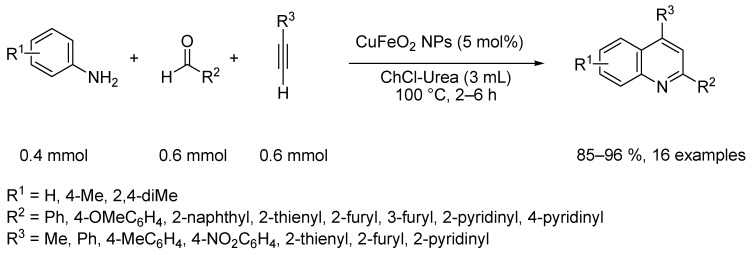
Synthesis of quinolines catalyzed by CuFeO_2_ NPs in ChCl-urea.

Abudken et al. recently reported a green and efficient protocol for the synthesis of 2,4-disubstituted quinolines using magnetically recoverable CuFeO_2_ nanoparticles as heterogeneous catalysts in a choline chloride/urea deep eutectic solvent (DES) system [91]. The quinoline synthesis was achieved through a multicomponent reaction between aryl aldehydes, aniline derivatives, and alkynes, affording a broad range of quinoline derivatives in high yields within relatively short reaction times. The catalytic system demonstrated broad substrate tolerance toward both electron-donating and electron-withdrawing substituents (Scheme 46).

The catalyst also exhibited remarkable recyclability. After nine consecutive reaction cycles, quinoline synthesis still proceeded in 92% yield, indicating excellent structural and catalytic stability. FT-IR, XRD, BET, SEM, TEM, and VSM analyses of the recovered catalyst confirmed that the morphology, crystallinity, and magnetic properties remained essentially unchanged after repeated use.

Mechanistically, the reaction proceeds through initial imine formation between the aldehyde and aniline, followed by CuFeO_2_-assisted activation of the alkyne and subsequent cyclization to furnish the quinoline framework. The authors proposed that the dual-metal nature of CuFeO_2_ enhances electrophilic activation and electron transfer processes, thereby accelerating annulation and aromatization steps.

A particularly important aspect of the work is the comparison of the CuFeO_2_ catalytic system with previously reported quinoline syntheses. The authors emphasized several advantages of CuFeO_2_ nanoparticles over conventional, green, or nanocomposite catalysts. First, the catalyst is composed of inexpensive earth-abundant metals (Cu and Fe), making it economically attractive compared with noble-metal catalysts. Second, the catalyst operates under ligand-free conditions and does not require surface functionalization or polymeric support, simplifying catalyst preparation and avoiding problems associated with ligand toxicity, leaching, or blockage of active sites. Third, the magnetic nature of CuFeO_2_ enables straightforward separation using an external magnet, facilitating catalyst recovery and reuse without filtration or centrifugation, and finally, CuFeO_2_ nanoparticles provided excellent yields under relatively mild conditions in fairly short reaction times.

Overall, this study introduces CuFeO_2_ nanoparticles in ChCl/urea DES as an efficient, reusable, and environmentally benign catalytic system for quinoline synthesis. The methodology offers several advantages over reported protocols, including high yields, operational simplicity, short reaction times, broad functional-group tolerance, easy magnetic recovery, ligand-free catalysis, and compatibility with green solvents, making it a promising sustainable approach for the synthesis of biologically relevant quinoline derivatives.

### 4.2. Solid Catalysts

The group of Pérez-Mayoral, building on earlier work in which they reported the first examples of Friedländer reactions efficiently catalyzed by a series of acidic activated carbons as environmentally friendly catalysts [92], has developed a coherent and impactful research line focused on the green synthesis of quinoline derivatives through heterogeneous catalysis, with particular emphasis on the design and application of recyclable solid materials [93,94,95]. Their work addresses key limitations of traditional quinoline syntheses, such as the use of corrosive acids, homogeneous catalysts, and harsh reaction conditions, by employing robust inorganic materials capable of promoting classical transformations (e.g., Friedländer-type condensations and related annulation processes) under milder and more sustainable conditions. By carefully tuning the acid-base properties, porosity, and surface functionality of materials, including modified metal oxides and hybrid inorganic solids, the group has demonstrated efficient catalytic systems that combine high activity with operational simplicity, facile separation, and reusability. A distinctive feature of this research line is the systematic integration of catalyst design, mechanistic understanding, and green chemistry principles. The reported methodologies typically proceed under solvent-free conditions or in environmentally benign solvents, minimize by-product formation, and enable straightforward catalyst recycling without significant loss of performance.

**Scheme 47:**
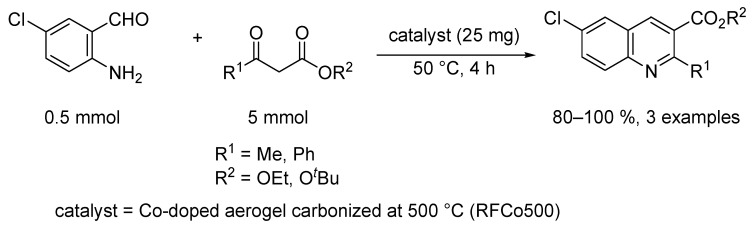
Friedländer condensation catalyzed by Co^0^-based aerogels.

In their 2017 study, the authors reported a new family of transition-metal-doped carbon aerogels as highly active and selective heterogeneous catalysts for the Friedländer synthesis of quinolines [93]. The materials were obtained through the polymerization of resorcinol and formaldehyde in the presence of transition-metal precursors, followed by carbonization and steam activation, leading to the in situ formation of well-dispersed zero-valent metal nanoparticles (Ni^0^, Co^0^, and Cu^0^) embedded within a porous carbon aerogel matrix. This synthetic strategy afforded robust nanostructured catalysts combining high surface area, controlled porosity, and homogeneous distribution of the active metal phase. Catalytic performance was evaluated using the condensation of 2-amino-5-chlorobenzaldehyde with a tenfold excess of ethyl acetoacetate as a model reaction. Under solvent-free conditions at 50°C, 25 mg of the Co^0^-doped aerogel provided the corresponding quinoline in quantitative yield after 4 h, highlighting the efficiency of these hybrid carbon-metal systems under mild and sustainable reaction conditions. Following these results, the authors reported a family of cobalt-based carbon aerogel nanocatalysts enabling efficient solvent-free quinoline synthesis via the Friedländer reaction [94]. Zero-valent Co nanoparticles were incorporated either by doping during aerogel formation or by post-impregnation onto preformed carbon aerogels. The catalysts promoted the condensation of 2-amino-5-chlorobenzaldehyde with β-ketoesters under mild conditions (Scheme 47).

Structure-activity correlations showed that catalytic performance is governed primarily by the dispersion and accessibility of Co^0^ nanoparticles, while the carbon matrix plays a secondary cooperative role. Mesoporous aerogels carbonized at a lower temperature (500 °C) exhibited highly dispersed Co nanoparticles (<4 nm) and delivered superior activity compared with samples carbonized at 1000 °C, where sintering produced larger particles (~15–17 nm) and lower yields. The optimal catalyst (Co-doped RFCo500) was recyclable for at least two cycles.

While the initial Co^0^-doped aerogels demonstrated the feasibility of cobalt-carbon systems in Friedländer quinoline synthesis, later work replaced embedded metallic Co^0^ with a series of surface-accessible cobalt-oxide nanoparticles supported on nanostructured carbons (mesoporous carbon aerogels, activated carbon, and oxidized carbon nanotubes), leading to markedly improved catalytic performance [95]. The catalysts were prepared by impregnation of carbon supports with cobalt salts followed by mild thermal treatment, yielding dispersed CoO nanoparticles anchored on the carbon surface.

**Scheme 48:**
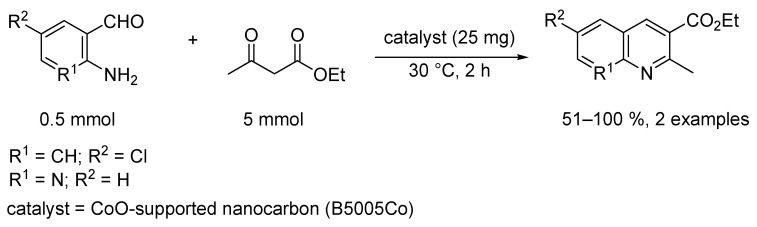
Friedländer reaction catalyzed by carbon-CoO-nanoparticles.

Under mild solvent-free conditions, CoO-carbon catalysts afforded quinolines in near-quantitative yields from 2-aminoaryl aldehydes and β-ketoesters. CoO was identified as the dominant active phase, more active than previously reported Co^0^-carbon aerogels, likely acting as a bifunctional site that activates both the carbonyl acceptor (Lewis acidic Co) and the enolizable β-ketoester (basic O) (Scheme 48).

Catalytic performance strongly depended on the porosity and morphology of the carbon support, which control nanoparticle dispersion, size, and accessibility. One point worth highlighting is that when metallic cobalt (Co^0^) is used in combination with aerogels, the carbon structure appears to have little influence on performance, as reported in the 2018 study [94]. However, when cobalt oxide (CoO) is employed with markedly different carbon materials, the carbon structure does exert a significant effect. Catalyst activity was also influenced by the cobalt precursor: when using acetate, the nanoparticles (diameter = 9 nm) are forced to be formed in the mesopores. Co-nanoparticles consequently become more accessible to the reactants than the ones located in narrower pores. The best catalyst showed high turnover frequencies and recyclability over multiple cycles with minimal deactivation.

**Scheme 49:**
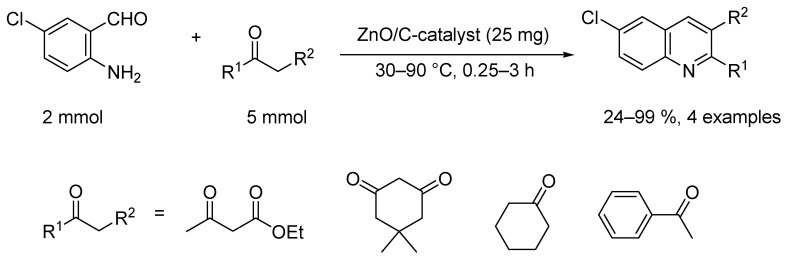
ZnO/C-catalyzed Friedländer reactions.

Following the development of Co-based carbon nanocatalysts, Pérez-Mayoral and co-workers reported a new generation of ZnO-carbon nanocomposites as heterogeneous catalysts for the Friedländer condensation [96]. The materials were prepared by dispersing ZnO nanoparticles onto porous carbon matrices derived from low-cost precursors, affording mesoporous or microporous supports with accessible Lewis-acidic Zn^2+^ surface sites. In analogy with earlier metal-carbon systems, catalytic activity was governed by the dispersion and surface accessibility of the metal oxide phase in combination with the porosity of the carbon framework. These ZnO-carbon hybrids efficiently promoted the synthesis of poly-substituted quinolines under solvent-free or otherwise green conditions, providing high yields and short reaction times with broad substrate scope. Importantly, the catalysts were readily recovered and reused over multiple cycles without significant loss of activity, confirming their structural robustness (Scheme 49).

The enhanced performance relative to unsupported ZnO was attributed to synergistic effects between dispersed ZnO active sites and the carbon support, which improves substrate concentration and mass transport at the catalytic interface.

In a 2024 study, Krishna and Roy reported the development of a Brønsted acid-functionalized graphitic carbon nitride (g-C_3_N_4_) as a metal-free heterogeneous catalyst for the Friedländer synthesis of quinoline derivatives [97]. Surface functionalization introduced strong acidic sites onto the g-C_3_N_4_ framework while preserving its layered morphology, high nitrogen content, and thermal stability. Detailed physicochemical characterization confirmed the successful incorporation of proton-donating groups and demonstrated that the catalytic performance correlates directly with the density and accessibility of these Brønsted acid sites.

**Scheme 50:**
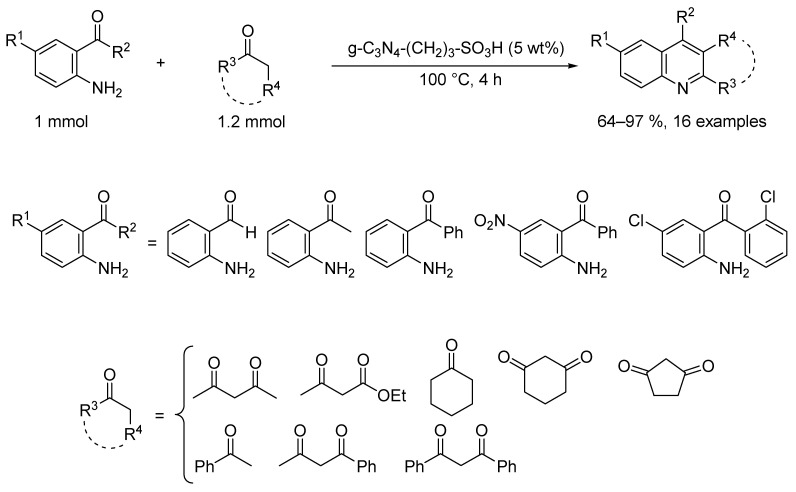
Synthesis of quinolines catalyzed by graphitic carbon nitride.

The catalytic system enabled the condensation of 2-aminoaryl ketones with activated carbonyl compounds under mild and environmentally benign conditions, affording a range of substituted quinolines in good to excellent yields. Notably, reactions were typically performed under solvent-free or green solvent conditions, and the solid catalyst could be easily recovered by filtration and reused over several cycles without significant loss of activity (Scheme 50).

Mechanistically, the catalyst promotes quinoline formation through acid-catalyzed activation of the carbonyl group, facilitating initial imine formation between the 2-aminoaryl ketone and the activated methylene carbonyl partner, followed by intramolecular cyclization and dehydration to furnish the quinoline core. The nitrogen-rich g-C_3_N_4_ matrix likely contributes cooperatively through hydrogen-bonding interactions and electronic modulation of surface acidity, enhancing substrate adsorption and transition-state stabilization.

**Scheme 51:**
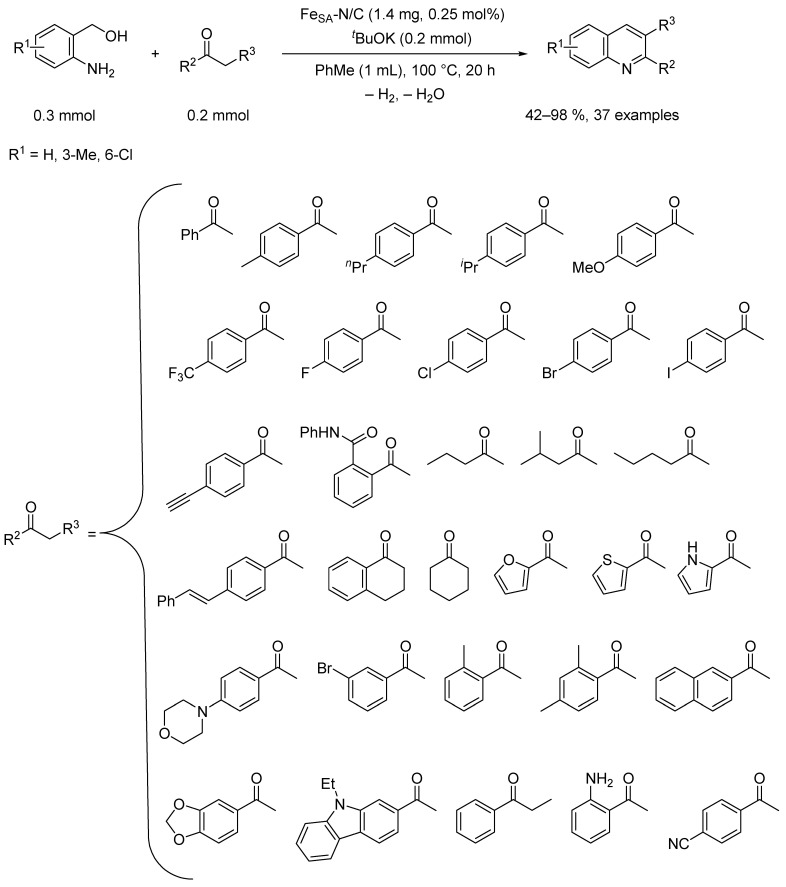
Fe_SA_-N/C-Catalyzed Coupling of 2-Aminobenzyl Alcohols with Ketones.

**Scheme 52:**
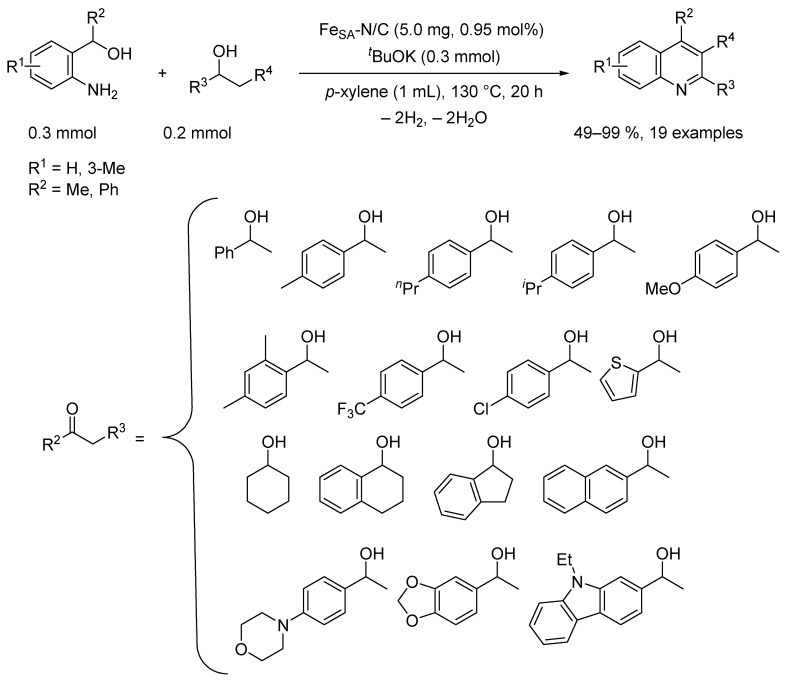
Fe_SA_-N/C-Catalyzed Coupling of 2-Aminobenzyl Alcohols with Secondary Alcohols.

In addition, single-atom catalysts (SACs), a novel category of catalytic systems that synergistically combine the advantages of homogeneous and metal nanoparticle catalysts, have emerged as a rapidly growing field of research over the past decade [98]. Lu et al. reported a highly efficient heterogeneous single-atom iron catalyst (Fe_SA_-N/C) for the acceptorless dehydrogenative coupling synthesis of quinolines from 2-aminobenzyl alcohols and ketones (Scheme 51) or secondary alcohols (Scheme 52) [99]. The catalyst consists of isolated Fe single atoms coordinated by nitrogen within a porous *N*-doped carbon matrix derived from pyrolysis of Fe/phenanthroline precursors on a sacrificial MgO template. Detailed characterization by HAADF-STEM, XANES, and EXAFS confirmed the isolated single-atom nature of the iron active sites. Under relatively mild conditions, the methodology provided a broad range of functionalized quinolines in moderate to excellent yields, demonstrating excellent tolerance toward diverse ketonic substrates, including aromatic, aliphatic, heteroaryl, and sensitive derivatives (Scheme 51).

When the acceptorless dehydrogenative coupling of 2-aminobenzyl alcohols was carried out using secondary alcohols instead of ketones, the methodology proved quite as effective in terms of both yields and substrate scope (Scheme 52).

Particularly noteworthy was the exceptionally high catalytic efficiency, with turnover numbers reaching up to 10^5^, significantly surpassing previously reported homogeneous and nanoparticle-based systems. Recycling studies demonstrated excellent catalyst stability over multiple cycles without significant loss of activity. Mechanistic investigations indicated that the reaction proceeds through initial iron-catalyzed acceptorless dehydrogenation of the alcohol substrates to the corresponding carbonyl intermediates, followed by base-promoted condensation and cyclization to furnish the quinoline core. Control experiments, including hot-filtration, acid-etching, and poisoning studies, confirmed the heterogeneous nature of the catalysis and highlighted the crucial role of the isolated Fe single-atom sites in the dehydrogenation process.

**Scheme 53:**
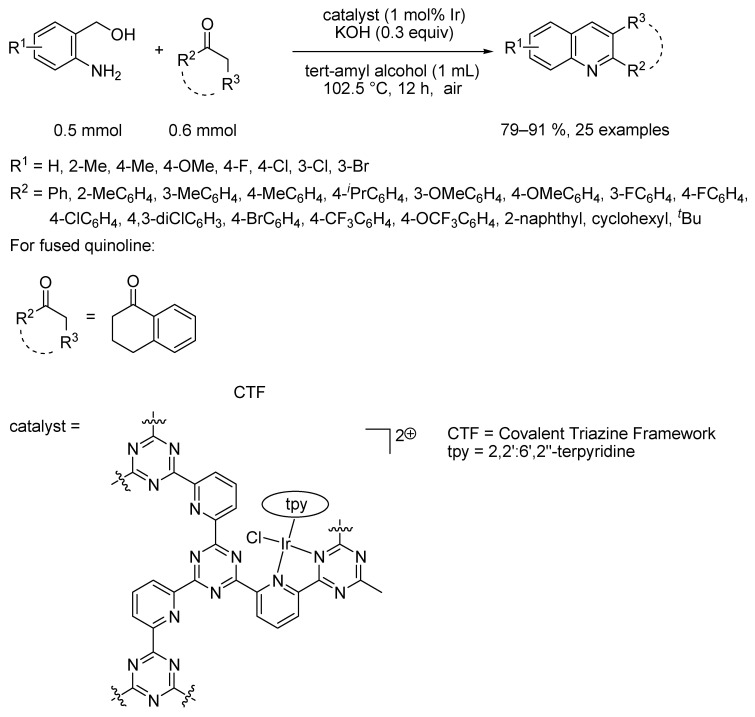
Synthesis of quinolines catalyzed by Ir(tpy)@CTF.

In a 2022 study [100], Chen et al. developed a recyclable heterogeneous Ir(III) terpyridine-based catalytic system supported on a covalent triazine framework (CTF), affording an efficient platform for the synthesis of quinoline derivatives from 2-aminobenzyl alcohols and ketones. The catalyst, termed Ir(tpy)@CTF, was prepared through immobilization of an Ir(III) terpyridine complex within the porous nitrogen-rich CTF matrix, generating a robust hybrid material combining the high activity of molecular Ir complexes with the operational advantages of heterogeneous catalysis [100]. Under optimized conditions, a broad range of quinolines was obtained in good to excellent yields through tandem dehydrogenation–condensation–cyclization sequences operating via borrowing-hydrogen/acceptorless dehydrogenative coupling pathways (Scheme 53).

Mechanistic studies suggested that the Ir catalyst promotes initial dehydrogenation of the amino alcohol substrate to the corresponding amino aldehyde, followed by condensation with the ketone partner and subsequent cyclization–aromatization to furnish the quinoline core. Notably, the heterogeneous catalyst exhibited excellent recyclability and structural stability over six catalytic cycles without significant loss of activity, highlighting the beneficial role of the covalent triazine framework in stabilizing the catalytically active Ir species. This work illustrates how heterogenized platinum-group-metal complexes can combine the efficiency of homogeneous hydrogen-transfer catalysis with the sustainability advantages associated with recyclable porous materials.

Overall, the heterogeneous and nanostructured catalytic systems discussed in this section exhibit several recurring mechanistic features despite the structural diversity of the catalysts employed. In most cases, quinoline formation proceeds through tandem condensation–cyclization–aromatization sequences promoted by cooperative surface activation of the reacting substrates. Metal oxides, supported nanoparticles, carbon-based materials, and hybrid nanocomposites generally act through Lewis-acidic or Brønsted-acidic surface sites that facilitate carbonyl activation, imine formation, and subsequent annulation steps. In several nanostructured systems, the catalytic performance is strongly influenced not only by the intrinsic activity of the metal species but also by the morphology, porosity, particle size, and surface accessibility of the support material, which collectively govern substrate adsorption, mass transport, and stabilization of reactive intermediates.

Another major mechanistic trend is the increasing implementation of bifunctional and cooperative catalytic effects. In many heterogeneous systems, the active metal center and the support participate synergistically in substrate activation, often combining acidic, basic, redox, or hydrogen-bonding functionalities within the same catalytic material. Nanostructured catalysts additionally benefit from high surface-area-to-volume ratios and enhanced dispersion of active sites, frequently leading to improved catalytic efficiency under milder and greener reaction conditions. Several methodologies also integrate photocatalytic or oxidative activation pathways, further expanding the mechanistic diversity of heterogeneous quinoline synthesis. Importantly, catalyst recoverability, recyclability, and operational stability emerge as central advantages of these systems, highlighting the growing role of heterogeneous and nanostructured catalysis in the development of sustainable quinoline synthetic methodologies.

## 5. Photocatalytic Synthesis

Recent studies have demonstrated the utility of photocatalysts, such as [Ru(bpy)_3_]^2+^ and fac-Ir(ppy)_3_, in enabling radical-based quinoline synthesis. These photocatalysts generate reactive species under visible-light irradiation, allowing for selective bond formation. In addition, visible-light-mediated photocatalysis has been widely utilized to produce various organic compounds [101,102]. They have garnered significant interest as a greener and more sustainable approach, utilizing photons as a clean energy source [103,104]. In addition to their broad biological significance, quinoline derivatives play a crucial role in fluorescent agents due to their compact structure. The nitrogen atom within the quinoline core enhances its coordination properties and hydrogen-bonding capabilities, making it a valuable scaffold in various chemical applications [20]. Quinoline-based molecules are well-known for their intramolecular charge transfer (ICT) properties and distinct photophysical characteristics. Their rigid and planar structure facilitates interactions with proteins and nucleic acids, making them highly effective for DNA and RNA imaging applications [105,106].

**Scheme 54:**
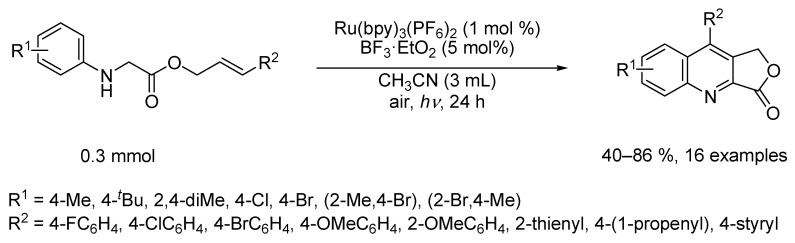
Visible-light-induced photocatalytic aerobic oxidation/Povarov reaction.

For instance, in 2016, Dong and co-workers pioneered a visible-light-induced Povarov cyclization of cinnamyl 2-(phenylamino)-acetates, leading to the formation of quinoline-fused lactones. This reaction was catalyzed using 1 mol% Ru(bpy)_3_(PF_6_)_2_ and 5 mol% BF_3_·Et_2_O, under the irradiation of a 23 W household light bulb (Scheme 54). They further developed a visible-light-driven photocatalytic aerobic oxidation/intramolecular Povarov cyclization, providing an efficient one-step route to these biologically significant core structures from readily available starting materials under mild reaction conditions [107].

**Scheme 55:**
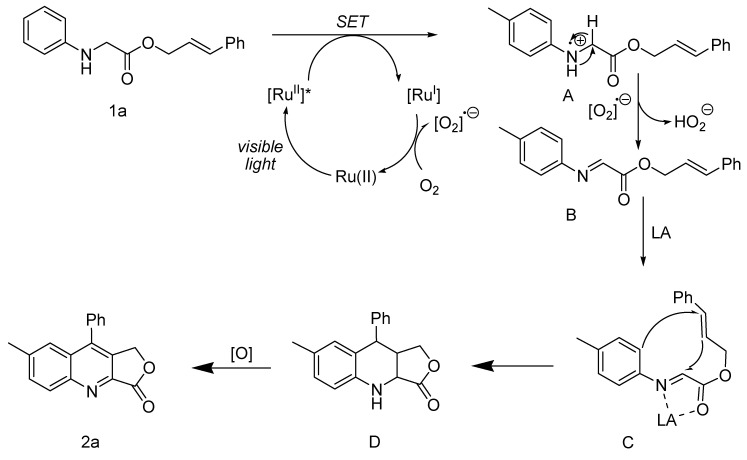
Mechanism for the intramolecular cyclization of *N*-arylglycine cinnamyl esters.

A plausible reaction mechanism is proposed in Scheme 55. Upon visible-light excitation, Ru(bpy)_3_(PF_6_)_2_ transitions to its excited Ru(II) state, which subsequently oxidizes 1a, generating the Ru(I) species and a radical amine cation (A). This intermediate undergoes further conversion to intermediate B, followed by transformation into intermediate D in the presence of a Lewis acid (LA). The desired product (2a) is then obtained via oxidation and aromatization.

**Scheme 56:**
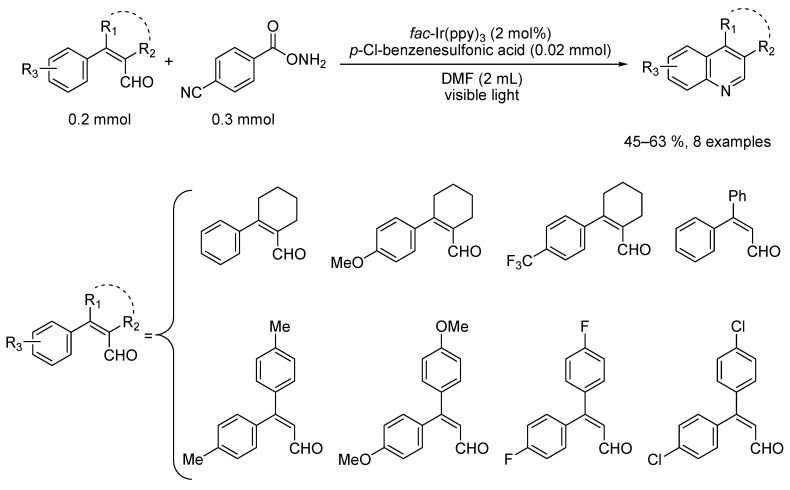
Visible-light-promoted and one-pot synthesis of quinolines.

Similarly, An and Yu reported a visible-light-mediated method for the in situ generation of *O*-acyloximes through the reaction of aldehydes with *O*-(4-cyanobenzoyl)hydroxylamine [108]. This process proceeds via the formation of an iminyl radical intermediate, which subsequently undergoes cyclization to yield the desired quinoline derivatives in good yields (Scheme 56). The method offers a mild route to access quinolines under photochemical conditions.

**Scheme 57:**
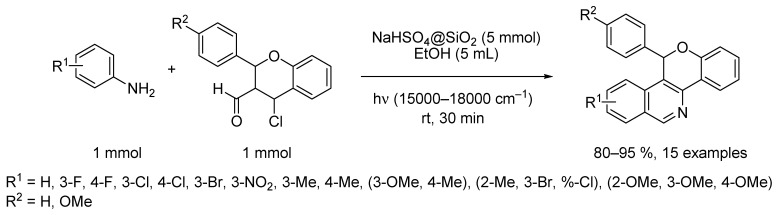
Synthesis of 6-phenyl-6*H*-chromeno [4,3-*b*] quinolines.

Suman et al. reported the synthesis of a series of chromeno[4,3-*b*]quinoline derivatives through the condensation–cyclization of chromenecarbaldehydes with anilines, employing a NaHSO_4_@SiO_2_ heterogeneous photocatalyst under visible-light irradiation (15,000–18,000 cm^−1^, ≈556–667 nm) in ethanol at room temperature. This protocol affords the target heterocycles in short reaction times and good yields [109]. The use of a reusable solid acid catalyst, a relatively benign solvent, and mild, energy-efficient conditions renders this key quinoline-forming step greener than many classical methodologies (e.g., Skraup or conventional Friedländer reactions). Nevertheless, the overall multistep sequence involves the preparation of chlorinated intermediates employing DMF/POCl_3_ and purification steps requiring chlorinated solvents and column chromatography. Consequently, although the final cyclization adheres to several green-chemistry principles, particularly catalysis, safer solvents, and reduced energy input, the synthetic route as a whole cannot be regarded as fully sustainable (Scheme 57).

**Scheme 58:**
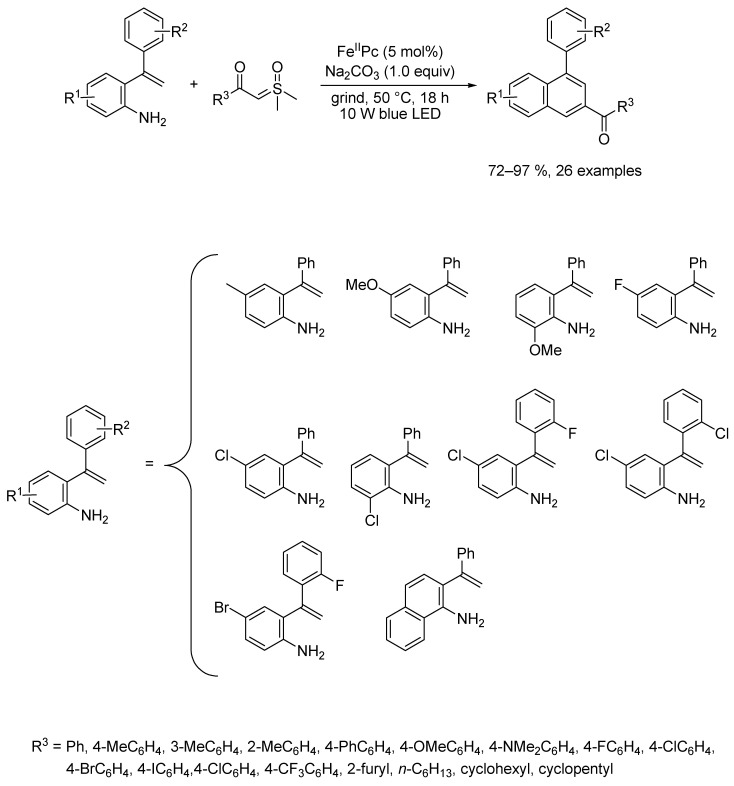
Photo-thermo-mechanochemical synthesis of quinolines.

A novel photo-thermo-mechanochemical strategy for the synthesis of quinolines catalyzed by iron(II) phthalocyanine has been reported by Liu et al. [110]. The method employs an inexpensive catalytic system, operates under solvent-free conditions, and offers operational simplicity together with broad substrate tolerance, providing a greener alternative to conventional thermal protocols (Scheme 58).

**Scheme 59:**
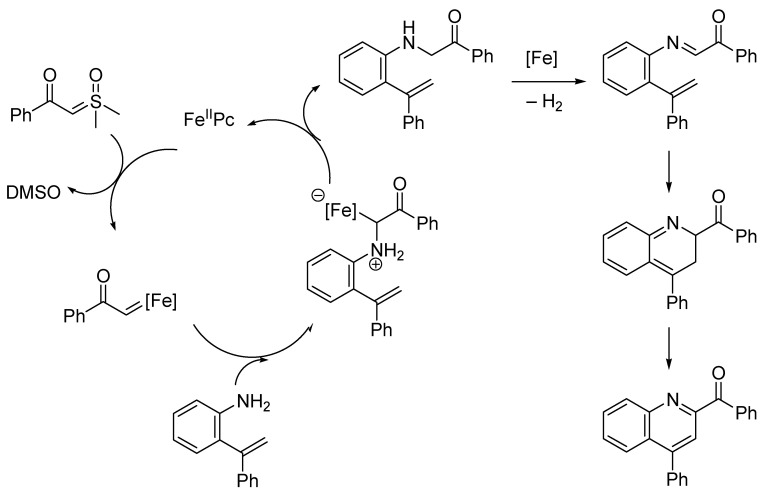
Mechanism for the photo-thermo-mechanochemical quinoline synthesis.

Control experiments suggested that the reaction proceeds through the initial formation of a secondary amine intermediate, which was identified as a key species in the transformation. Subsequent aromatization/cyclization to the quinoline product was strongly promoted by Na_2_CO_3_, while alternative bases such as K_2_CO_3_ or Cs_2_CO_3_ led to significantly reduced yields. Additional experiments demonstrated that iron(II) phthalocyanine was essential for the reaction to occur. The proposed mechanism is depicted in Scheme 59.

The photocatalytic methodologies discussed in this section share several common mechanistic features centered on visible-light-induced activation and radical-mediated annulation processes. In most cases, excitation of the photocatalyst generates highly reactive excited states capable of initiating single-electron transfer (SET) events, thereby producing radical or radical-ion intermediates that subsequently undergo C-C and C-N bond-forming transformations leading to quinoline frameworks. Depending on the catalytic system, these pathways may involve oxidative quenching, reductive quenching, hydrogen-atom transfer, or energy-transfer processes, frequently operating under aerobic conditions in which molecular oxygen serves as the terminal oxidant.

A major advantage of photocatalytic quinoline synthesis is the ability to promote annulation reactions under comparatively mild conditions while minimizing the need for harsh oxidants, elevated temperatures, or stoichiometric activating reagents. Several methodologies combine photocatalysis with tandem condensation, cyclization, and oxidative aromatization sequences, enabling efficient construction of quinoline scaffolds through cascade radical processes. Recent developments additionally highlight the growing importance of synergistic catalytic strategies integrating photoredox catalysis with transition-metal catalysis, heterogeneous photocatalysts, or nanostructured materials. Collectively, these studies demonstrate how visible-light activation has emerged as a powerful and sustainable mechanistic platform for modern quinoline synthesis.

## 6. Catalyst-Free and Green Reaction Conditions

This section brings together a range of quinoline synthesis methodologies that are unified not by the nature of the catalyst, but by the reaction conditions under which they are performed. In contrast to catalyst-driven approaches discussed in previous sections, these strategies emphasize operational parameters, such as the absence of solvent or catalyst, the use of alternative energy inputs (e.g., microwave irradiation), or the employment of non-conventional reaction media such as ionic liquids, to enhance efficiency and sustainability. Although diverse in their implementation, these methodologies share a common objective: the reduction of environmental impact through minimization of waste, energy consumption, and hazardous reagents. Ionic liquids are included within this framework as versatile reaction media that, depending on their design, may also exhibit catalytic behavior, thereby bridging the boundary between solvent and catalyst. Grouping these approaches under a single section highlights their conceptual alignment with green chemistry principles and provides a coherent perspective on condition-driven strategies for quinoline synthesis.

### 6.1. Thermal and Microwave-Assisted Catalyst-Free Methods

Recent efforts have focused on developing sustainable, catalyst-free strategies for quinoline synthesis. These approaches take advantage of the inherent reactivity of substrates under thermal or microwave conditions [4,111,112,113]. The catalyst-free reaction represents a significant step forward in pursuing efficient, sustainable, and cost-effective quinoline synthesis.

**Scheme 60:**
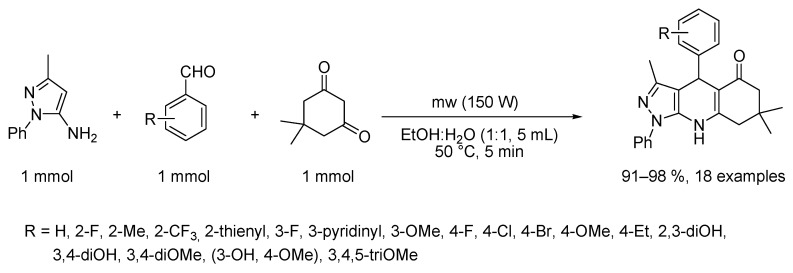
Microwave-assisted synthesis of quinolines in aqueous ethanol.

Robert Khumalo et al. have developed a facile, rapid, and environmentally friendly microwave-assisted multicomponent protocol for the synthesis of novel pyrazolo-[3,4-*b*]-quinolines without additional heating using aqueous ethanol as the reaction medium. This methodology is marked by its operational simplicity, mild conditions, short reaction time, high selectivity, and absence of byproduct formation [114]. Furthermore, the protocol demonstrates excellent reproducibility and holds great potential for application in green and sustainable organic synthesis (Scheme 60).

**Scheme 61:**
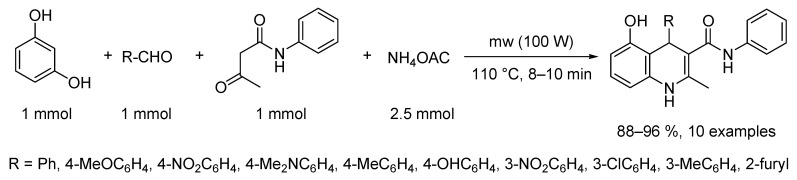
Microwave-assisted synthesis of 1,4-dihydroquinoline derivatives.

Using microwave irradiation, as well, Chidurala et al. successfully synthesized substituted 1,4-dihydroquinolines through a one-pot multicomponent reaction involving resorcinol, aromatic aldehydes, acetoacetanilide, and ammonium acetate [115]. This efficient approach is notable for being atom-economical, catalyst-free, and environmentally benign. In addition, the method offers a practical alternative for the rapid construction of quinoline derivatives under mild and green reaction conditions (Scheme 61).

**Scheme 62:**
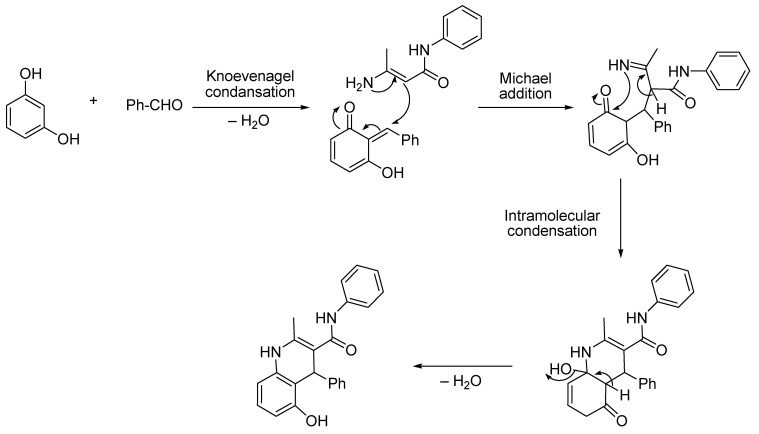
Proposed reaction mechanism for the MW-assisted synthesis of 1,4-dihydroquinoline derivatives.

A plausible reaction mechanism for the formation of substituted 1,4-dihydro-5-hydroxy-2-methyl-*N*-,4-diphenylquinoline-3-carboxamide derivatives is illustrated in Scheme 62. The process likely begins with the formation of a Knoevenagel product, followed by the generation of 3-amino-*N*-phenylbut-2-enamide through the condensation of acetoacetanilide with ammonia. These intermediates then combine to form a Michael adduct, which undergoes intramolecular cyclization via the amino group and the carbonyl of the 5-hydroxycyclohexa-2,4-dienone, yielding the final quinoline product.

In 2020, Liao and Zhu reported a mild, catalyst-free, three-component reaction for the construction of highly substituted pyrrolo[2,3-*h*]quinoline derivatives, a fused quinoline family of considerable pharmacological relevance (anti-angiogenic, aromatase-inhibitory, and STAT3-inhibitory activities) [116]. The method couples but-2-ynedioates, 4-aminoindoles, and aldehydes (or isatins) in a DMSO-water mixed solvent (*v*/*v* = 2.5:1) at 80 °C, giving access to two new series of dihydropyrrolo[2,3-*h*]quinolines and dihydropyrroloquinoline-spirooxindoles in moderate to good yields.

Solvent screening revealed that no reaction occurs in anhydrous organic solvents, whereas water is essential for reactivity, and DMSO strongly promotes the transformation. Addition of catalysts such as AcOH, TfOH, AlCl_3_, FeCl_3_, Cu(OAc)_2_, and N(Et)_3_ was detrimental, demonstrating that the reaction operates best under strictly catalyst-free conditions.

**Scheme 63:**
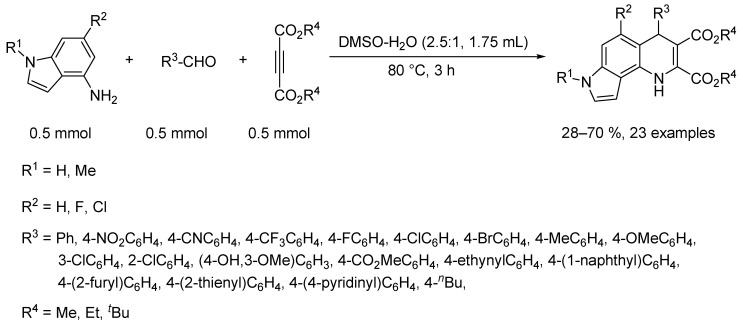
Catalyst-free 3CR synthesis of pyrrolo[2,3-*h*]quinolines.

The substrate scope showed broad tolerance for aromatic aldehydes, including electron-withdrawing (NO_2_, CN, CF_3_, halogens) and electron-donating (Me, OMe) substituents, as well as heteroaryl aldehydes (furyl, thienyl, pyridyl), furnishing the corresponding pyrrolo[2,3-*h*]quinolines. 4-Aminoindoles bearing substituents such as F, Cl, and Me were also compatible (Scheme 63). Additionally, the dihydro compound could be oxidized to the corresponding pyrrolo[2,3-*h*]quinoline at room temperature using Cu(NO_3_)_2_.

**Scheme 64:**
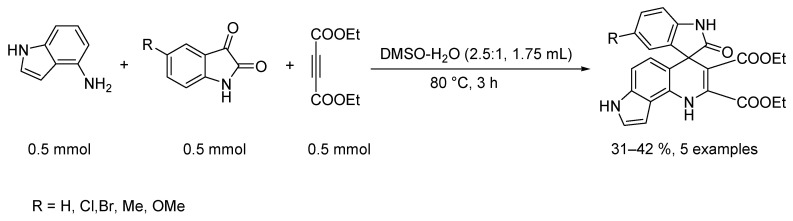
Catalyst-free 3CR synthesis of dihydropyrroloquinoline-spirooxindole derivatives.

Replacement of aldehydes by isatins enabled the synthesis of dihydropyrroloquinoline-spirooxindoles, providing access to hybrid quinoline-spiro frameworks that are attractive for medicinal chemistry (Scheme 64).

**Scheme 65:**
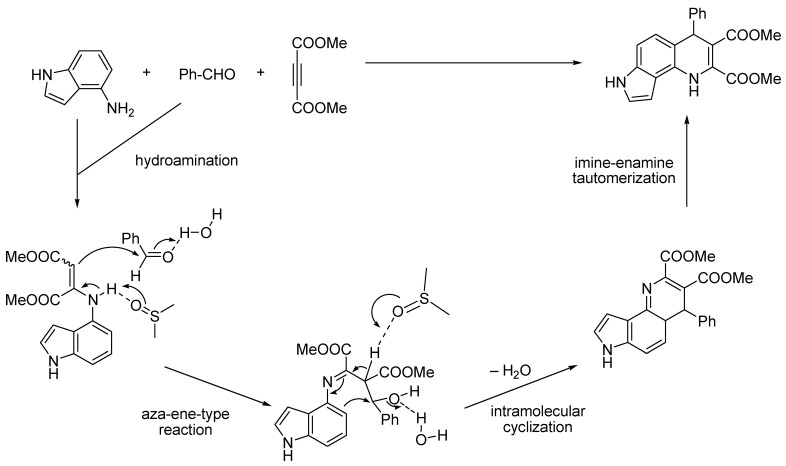
Proposed mechanism for the synthesis of pyrroloquinolines.

Considering the obtained experimental results in their previous studies [117,118], and the catalytic effect of water on the carbonyl group by hydrogen bonding [119,120], the authors propose a mechanism for the water-DMSO-promoted 3CR synthesis of pirrolo quinolines, as depicted in Scheme 65.

### 6.2. Solvent-Free Synthesis

The selection of an appropriate solvent is a crucial factor in green synthesis, as it directly influences reaction kinetics, selectivity, safety, and the environmental footprint of chemical processes. Solvents often account for a major portion of waste in synthetic protocols, and their impact extends to energy usage, toxicity, and post-reaction purification demands. In alignment with the principles of green chemistry, there is a growing emphasis on utilizing environmentally benign and sustainable solvents such as water, ethanol, and other bio-based alternatives while actively reducing or eliminating the use of harmful volatile organic compounds. In recent years, solvent-free and solvent-minimized methods have gained momentum as practical and eco-conscious strategies for synthesizing aryl quinolines. These approaches not only reduce hazardous waste and energy consumption but also align with global efforts to develop cleaner and safer chemical processes with a lower environmental burden [8,9,121].

**Scheme 66:**
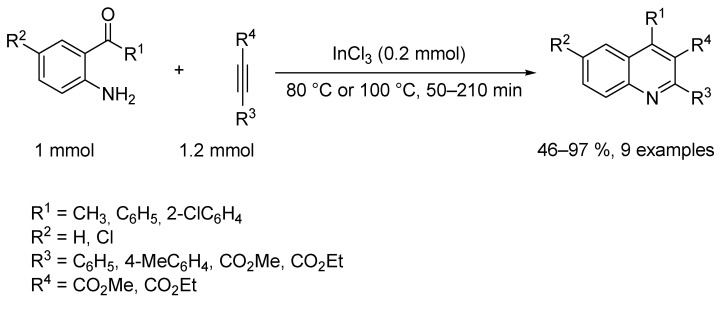
Solvent-free synthesis of quinolines catalyzed by InCl_3_.

Chanda, Verma, and Singh reported a highly versatile and regioselective quinoline synthesis catalyzed by InCl_3_, based on the coupling of 2-aminoaryl ketones with structurally diverse electrophilic partners [66]. The methodology proceeds efficiently under either refluxing acetonitrile, as discussed in the previous section on indium catalysis, or under solvent-free conditions, thereby providing an operationally simple and environmentally benign alternative to classical quinoline syntheses. Notably, the reaction of 2-aminoaryl ketones with alkynes under solvent-free conditions afforded the corresponding quinoline derivatives in moderate to excellent yields (Scheme 66).

**Scheme 67:**
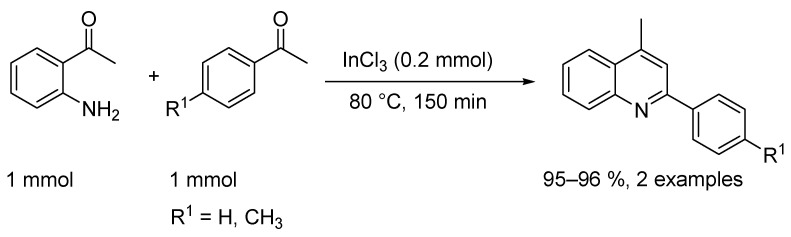
Synthesis of quinolines from acetophenone derivatives.

When 2-aminoacetophenone was reacted with acetophenone and 4-methyl acetophenone in the presence of 20 mol% of InCl_3_, the corresponding quinolines were obtained in excellent yields (Scheme 67).

**Scheme 68:**
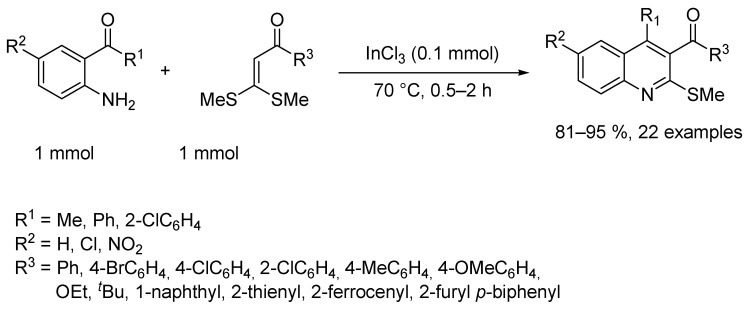
Reaction of 2-aminoacetophenones with α-oxoketene dithioacetals.

Furthermore, α-oxoketene dithioacetals were tested as electrophilic partners in the Friedländer reaction. α-Oxoketene dithioacetals are known as highly versatile intermediates in organic synthesis, yet reports on their use in quinoline synthesis remain limited [122,123]. Reactions of 2-aminoarylketones and α-aroylketene dithioacetals furnished the corresponding quinolines in excellent yields (Scheme 68).

Overall, this study establishes InCl_3_ as a general and chemoselective catalyst for diversity-oriented quinoline synthesis, representing a valuable contribution to modern heterocyclic methodology. However, the potential toxicity of indium compounds, particularly upon inhalation or parenteral exposure, despite their limited absorption via ingestion, may constrain their appeal within a sustainability-oriented framework [124,125].

This underscores the potential for further exploration and development of new synthetic routes utilizing ketene dithioacetals for accessing quinoline frameworks, especially under green and solvent-free conditions that align with sustainable chemistry practices [126].

**Scheme 69:**
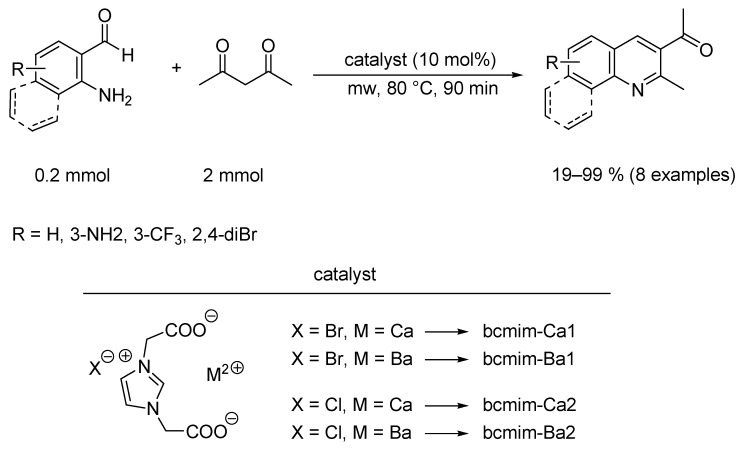
Synthesis of quinolines catalyzed by imidazolium-dicarboxylate.

Albert-Soriano et al. reported the use of heterogeneous calcium- and barium-based imidazolium-dicarboxylate (bcmim) metal–organic framework catalysts for the solvent-free synthesis of quinolines via the Friedländer reaction, offering a sustainable alternative to conventional transition-metal systems [127]. These materials are readily prepared under mild conditions and exhibit tunable catalytic activity depending on both the metal center and the linker counterion. The catalysts efficiently promote the condensation of 2-aminoaryl aldehydes or ketones with carbonyl compounds, affording quinolines in moderate to excellent yields, often under microwave irradiation with short reaction times. In general, barium-based systems display higher activity, whereas calcium analogues provide complementary reactivity, enabling substrate-dependent optimization. Furthermore, the catalysts operate under truly heterogeneous conditions with negligible metal leaching and can be reused for up to four cycles with minimal loss of activity (Scheme 69).

**Scheme 70:**
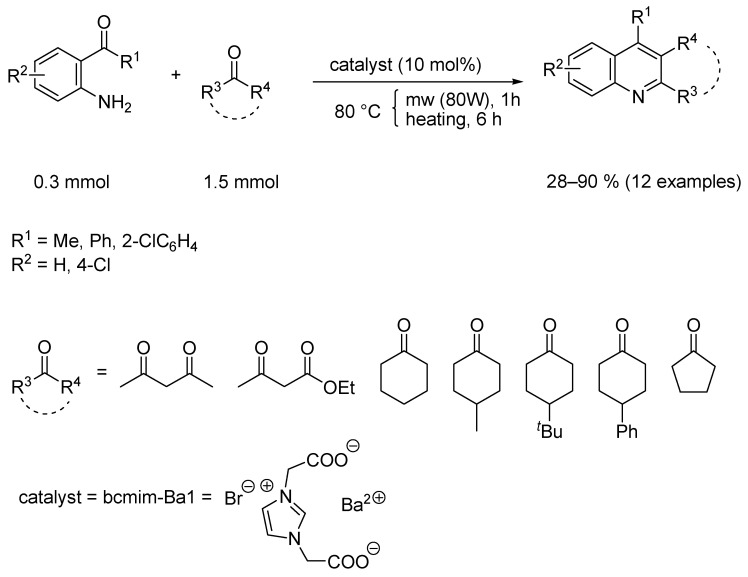
Friendländer reaction catalyzed by imidazolium-based catalyst.

Once bcmim-Ba1 was identified as the most active catalyst among the series, further investigations were conducted under both microwave-assisted and conventional thermal conditions to evaluate the scope with respect to 2-aminoacetophenones and ketones. In general, microwave irradiation provided comparable or improved conversions relative to conventional heating, while significantly reducing reaction times (Scheme 70).

**Scheme 71:**
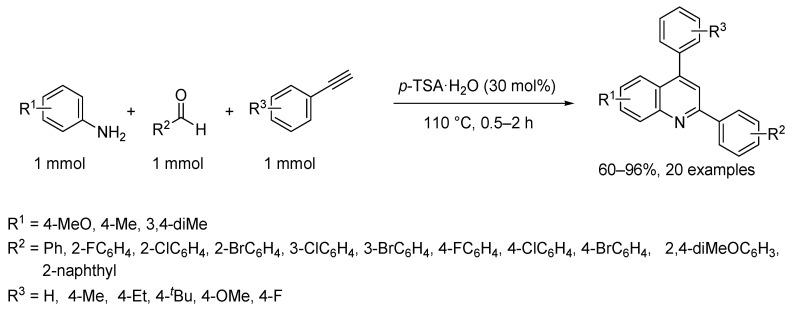
*p*-TSA-catalyzed synthesis of 2,4-diarylquinoline.

In a recent communication, Faraz and co-workers reported a highly efficient, metal- and solvent-free three-component approach to 2,4-diarylquinolines, employing arylamines, aryl aldehydes, and arylacetylenes in the presence of *p*-toluenesulfonic acid monohydrate (*p*-TSA·H_2_O) [31]. The transformation proceeds under neat conditions at 110 °C with 30 mol% *p*-TSA·H_2_O, representing a rare example of an A^3^-type quinoline synthesis that operates without a metal catalyst (Scheme 71). Optimization studies revealed that alternative Brønsted acids, including TfOH, CSA, and acetic acid, afforded significantly lower yields, thereby underscoring the distinctive efficiency of *p*-TSA·H_2_O.

The substrate scope demonstrated broad tolerance toward electron-rich arylamines (e.g., *p*-anisidine, *o*-anisidine, *p*-toluidine, and 3,4-xylidine), substituted benzaldehydes (F, Cl, Br, and naphthyl), and aryl acetylenes (Me, Et, *^t^*Bu, OMe, and F), affording 2,4-diarylquinolines in generally good yields. Notably, the majority of the products obtained were previously unreported. In contrast, electron-deficient anilines bearing halogen, NO_2_, or CF_3_ substituents were unreactive, underscoring the requirement for sufficiently nucleophilic amines. To further probe substrate scope, the use of an aliphatic aldehyde (butyraldehyde) in combination with *p*-anisidine and phenylacetylene was examined; interestingly, this reaction furnished 3-ethyl-6-methoxy-2-propylquinoline rather than the expected 6-methoxy-4-phenyl-2-propylquinoline.

**Scheme 72:**
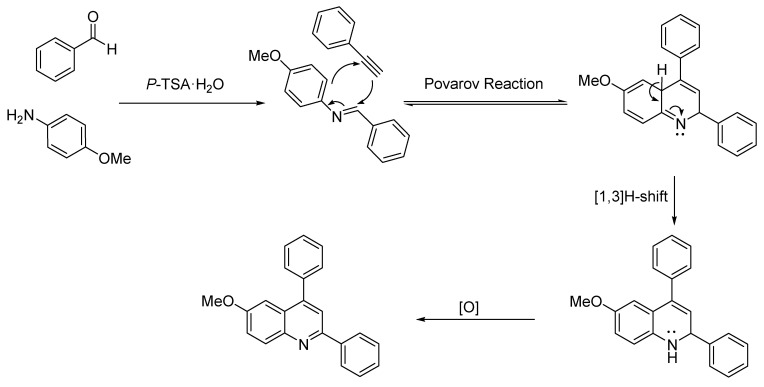
Mechanism of the *p*-TSA-catalyzed synthesis of 2,4-diarylquinoline.

Mechanistically (Scheme 72), *p*-TSA promotes initial imine formation between the arylamine and aldehyde, followed by a Povarov-type [4+2] cycloaddition with the arylacetylene, a subsequent [1,3]-hydrogen shift, and final aerobic oxidation to yield the aromatized 2,4-diarylquinoline.

### 6.3. Ionic Liquids

In recent years, ionic liquids (ILs) have gained increasing interest in the realm of green chemistry. Initially introduced as environmentally friendly reaction media, they possess unique chemical and physical properties such as nonvolatility, nonflammability, thermal stability, and tunable miscibility. Their recyclability has further enhanced their role as green solvents in various organic transformations [17]. In addition, they are excellent solvents and/or catalysts for organic synthesis, including the Povarov reaction.

**Scheme 73:**
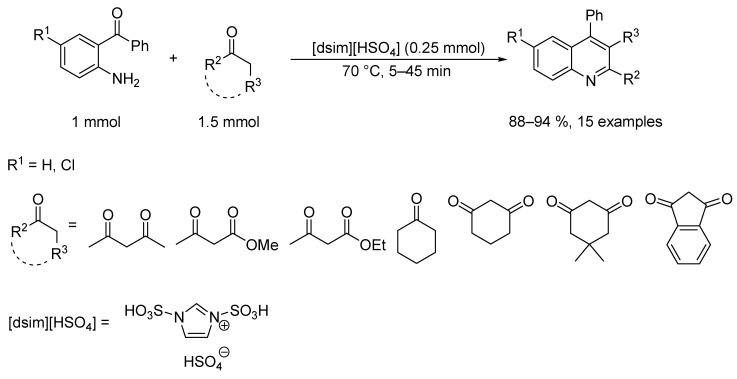
Synthesis of substituted quinolines catalyzed by [dsim][HSO_4_].

Ionic liquids have attracted considerable interest as efficient and versatile catalysts in organic synthesis, particularly in the Friedländer quinoline synthesis. They have also been used as effective alternatives to the volatile organic compounds in organic synthesis. These compounds are composed of ions and are liquid at room temperature. Their distinctive physicochemical properties such as low volatility, high thermal stability, and adjustable acidity make them well-suited for catalytic applications. A crucial factor in their catalytic behavior is the correlation between the anion’s basicity and overall catalytic efficiency. It is widely recognized that the anion’s nature affects the electrophilicity of the cation, which subsequently alters the acidity of the ionic liquid, thereby influencing its catalytic performance. The classification of reported ionic liquids is primarily based on their cationic structures [128]. Shirini and co-workers reported an environmentally benign and efficient protocol for the synthesis of substituted quinolines via the Friedländer annulation, using imidazolium-1,3-disulfonic acid hydrogen sulfate [dsim][HSO_4_] as a Brønsted-acidic ionic liquid catalyst. The method operates under solvent-free conditions at 70 °C, providing a significant improvement over classical Friedländer protocols, which typically require strong mineral acids, organic solvents, or harsh conditions [129]. The key advantages of this approach include rapid reaction times, mild reaction conditions, high conversion rates, straightforward catalyst preparation and separation, excellent yields, and the elimination of harmful organic solvents and toxic catalysts. Furthermore, [dsim][HSO_4_], being water-soluble, can be easily separated from the organic products by washing with water and reused at least four times without noticeable loss of activity (Scheme 73).

**Scheme 74:**
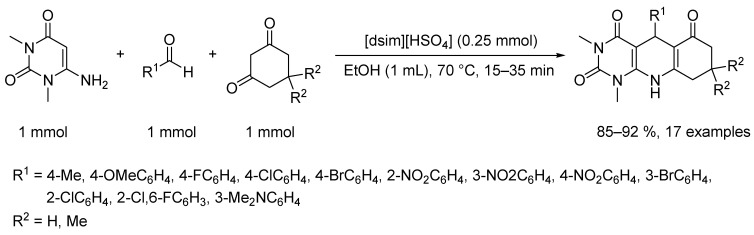
Multicomponent synthesis of pyrimido[4,5-*b*]quinolines using [dsim][HSO_4_].

In a complementary study, the same research group extended the application of [dsim][HSO_4_] to the multicomponent synthesis of fused quinoline systems, specifically pyrimido[4,5-*b*]quinoline derivatives, which are of high interest due to their anticancer, antimicrobial, antimalarial, and anti-inflammatory activities [130]. The reported method involves a three-component one-pot reaction between aromatic aldehydes, 6-amino-1,3-dimethyluracil, and cyclic 1,3-dicarbonyl compounds (e.g., dimedone), catalyzed by [dsim][HSO_4_] in ethanol at 70 °C. Under these mild conditions, a wide variety of substituted pyrimido[4,5-*b*]quinolines were obtained in high to excellent yields in short reaction times, regardless of whether the aldehyde carried electron-donating or electron-withdrawing substituents. Both dimedone-based and cyclohexanedione-based systems performed efficiently, highlighting the broad substrate scope of the protocol (Scheme 74).

**Scheme 75:**
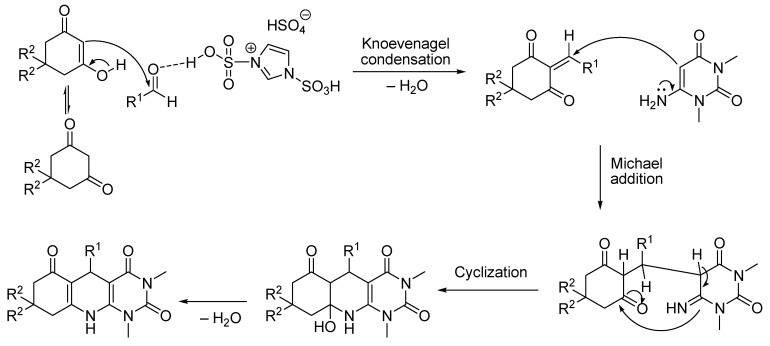
Proposed reaction mechanism for the synthesis of pyrimido[4,5-*b*]quinolines using [dsim][HSO_4_].

The authors proposed a Brønsted-acid-assisted mechanism in which [dsim][HSO_4_] activates the carbonyl group of the methylene-active compound, enabling either an initial aldol-type condensation followed by intramolecular cyclization, or imine formation between the *o*-amino ketone and the activated carbonyl compound, followed by cyclodehydration to furnish the quinoline nucleus. In both pathways, [dsim][HSO_4_] plays a dual role as proton donor and ionic reaction medium, stabilizing charged intermediates and accelerating ring closure. The proposed reaction mechanism is illustrated in Scheme 75.

Sun and co-workers reported an efficient, metal-free, three-component reaction for the construction of substituted quinoline frameworks. The transformation proceeds through initial imine formation between anilines and carbonyl partners, followed by intramolecular cyclization and final aromatization to furnish the desired product [17].

The method is based on the reaction of aromatic aldehydes, (*E*)-3-aminobut-2-enenitrile, and cyclic 1,3-dicarbonyl compounds in the ionic liquid [bmim][Br] (1-butyl-3-methylimidazolium bromide) at 50 °C.

**Scheme 76:**
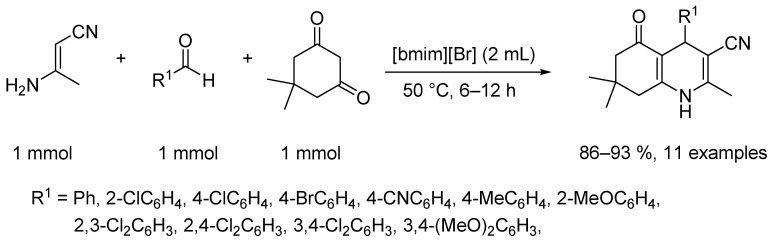
Synthesis of 4-arylquinoline-3-carbonitriles in [bmim][Br].

When dimedone was employed as the cyclic 1,3-dicarbonyl partner, a broad range of 4-arylquinoline-3-carbonitriles were obtained in excellent yields within 6–12 h. Substrates bearing both electron-donating (Me, OMe) and electron-withdrawing (Cl, Br, CN) substituents on the aromatic aldehyde were well tolerated, demonstrating the broad substrate scope of the transformation. (Scheme 76).

**Scheme 77:**
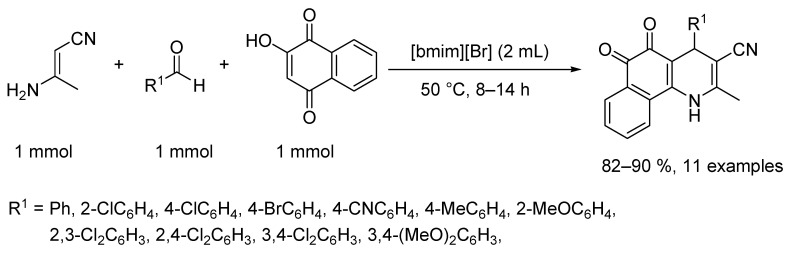
Synthesis of 4-arylbenzo[*h*]quinoline-3-carbonitriles in [bmim][Br].

Replacement of dimedone by 2-hydroxy-1,4-naphthoquinone (lawsone) led to a second family of polycyclic systems, namely 1,4,5,6-tetrahydro-2-methyl-5,6-dioxo-4-arylbenzo[*h*]quinoline-3-carbonitriles, which were also obtained in high yields (82–90%) under identical conditions (Scheme 77).

This demonstrates that the protocol is not limited to simple quinolines but can also efficiently construct extended fused quinoline frameworks.

A key practical advantage of this protocol is the recyclability of [bmim][Br]. After product isolation by simple water addition and filtration, the ionic liquid was recovered and reused at least four times without loss of activity, highlighting the sustainability of the system.

**Scheme 78:**
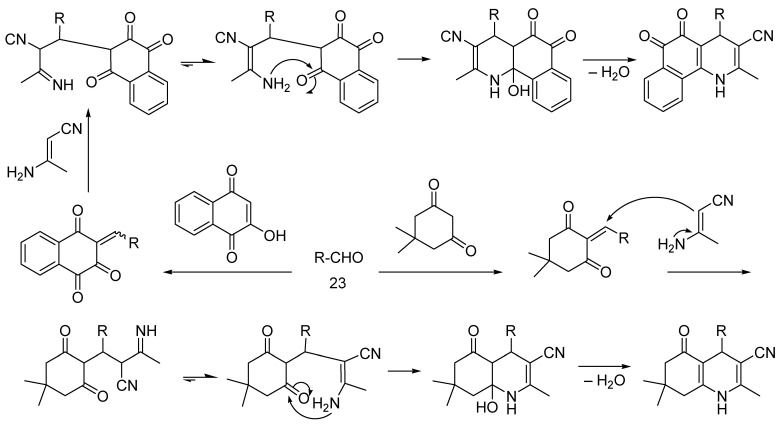
Proposed mechanism for the synthesis of 4-arylquinoline-3-carbonitrile and 4-arylbenzo[*h*]quinoline-3-carbonitrile derivatives.

The authors proposed a multistep domino mechanism involving Knoevenagel condensation between the aldehyde and the cyclic 1,3-dicarbonyl compound, Michael addition of (*E*)-3-aminobut-2-enenitrile, intramolecular cyclization, and dehydration, furnishing the quinoline or benzo[*h*]quinoline core (Scheme 78). Here, the ionic liquid acts as a highly polar, hydrogen-bonding medium that stabilizes charged intermediates and promotes these sequential transformations without the need for added catalysts.

Sarma, Saikia, and Borah reported a sustainable and efficient one-pot strategy for the synthesis of 2-styrylquinoline derivatives using -SO_3_H functionalized Brønsted acidic imidazolium ionic liquids (ILs) as recyclable catalysts and reaction media [131]. The methodology integrates a sequential Friedländer annulation followed by a Knoevenagel condensation under solvent-free thermal conditions, avoiding isolation of intermediates and significantly reducing reaction time and waste.

**Scheme 79:**
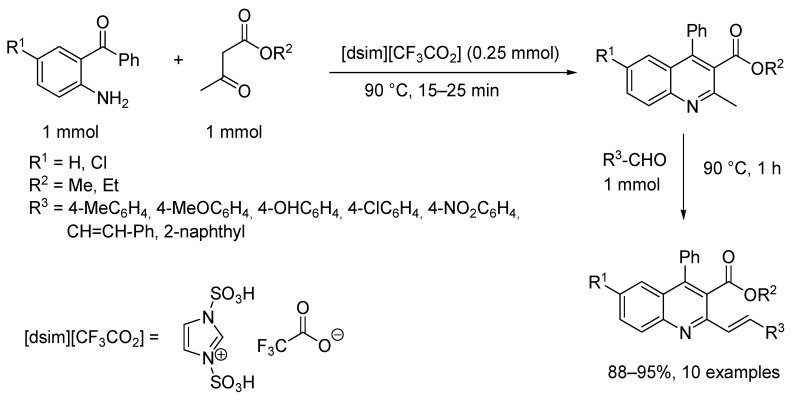
Preparation of 2-styrylquinoline derivatives with acidic IL.

Four task-specific sulfoimidazolium ILs with tunable Brønsted acidity were synthesized and systematically evaluated. Their acid strengths were quantified using Hammett acidity functions and correlated with catalytic performance. Among them, imidazolium-1,3-disulfonic acid trifluoroacetate ([dsim][TFA]) emerged as the most effective catalyst, providing excellent yields of 2-styrylquinolines using 25 mol% catalyst. Less acidic ILs required higher loadings (up to 50 mol%) to achieve comparable conversions, while the tosylate-based IL decomposed at relatively low temperature and was unsuitable under optimized conditions (Scheme 79).

Compared with traditional protocols, the present approach offers clear advantages in terms of operational simplicity, reduced reaction times, solvent-free conditions, and high product yields. The protocol works for various substituted 2-aminoaryl ketones, β-keto esters, and aromatic aldehydes, although aliphatic aldehydes were unreactive. Structural confirmation of the products, including *E*-configured styrylquinolines, was supported by spectroscopic data and single-crystal X-ray analysis. Importantly, the most active IL could be recycled for at least four consecutive runs with minimal loss of activity, highlighting its practical applicability.

Despite the methodological diversity encompassed within catalyst-free and green reaction conditions, several common mechanistic and conceptual features emerge across these approaches. Most methodologies rely on intrinsically favorable condensation–cyclization–aromatization sequences that proceed efficiently without the need for transition-metal catalysts when combined with appropriate activation strategies such as microwave irradiation, solvent-free conditions, or ionic-liquid media. In many cases, elevated local temperatures, enhanced mass transfer, hydrogen-bonding interactions, or increased substrate concentration facilitate the formation of key imine or enamine intermediates, thereby accelerating annulation and aromatization processes leading to quinoline frameworks.

From a sustainability perspective, these methodologies are unified by their emphasis on process intensification, operational simplicity, and waste minimization. Solvent-free protocols reduce solvent consumption and improve atom economy, while microwave-assisted methods significantly decrease reaction times and energy input. Ionic liquids frequently provide dual roles as solvent and promoter, often stabilizing reactive intermediates and facilitating catalyst recycling or improved selectivity. Catalyst-free methodologies additionally eliminate concerns associated with metal contamination, catalyst recovery, and toxicity. Collectively, these approaches demonstrate that greener quinoline synthesis can often be achieved not only through catalyst design but also through careful optimization of reaction media, activation methods, and process conditions.

## 7. Conclusions and Future Outlook

The synthesis of quinoline derivatives continues to represent a highly dynamic and strategically important area of heterocyclic chemistry owing to the broad relevance of quinoline scaffolds in medicinal chemistry, materials science, catalysis, and agrochemical applications. As highlighted throughout this review, recent years have witnessed remarkable progress in the development of increasingly efficient, selective, and sustainable methodologies for quinoline construction. In particular, the transition from traditional stoichiometric and harsh synthetic protocols toward catalytic, atom-economical, and environmentally benign approaches has significantly reshaped the field.

Among the most important advances is the growing implementation of catalytic systems based on earth-abundant transition metals such as iron, cobalt, and nickel, which are emerging as viable and sustainable alternatives to noble-metal catalysts. These systems have demonstrated remarkable efficiency in borrowing-hydrogen and acceptorless dehydrogenative coupling methodologies, enabling quinoline synthesis from readily available alcohol feedstocks while generating molecular hydrogen and/or water as the only stoichiometric by-products. At the same time, platinum-group-metal catalysts, particularly ruthenium- and iridium-based complexes, continue to provide highly efficient and mechanistically sophisticated platforms for tandem annulation processes, often operating through metal–ligand cooperative pathways.

Significant progress has also been achieved in heterogeneous catalysis, nanostructured materials, photocatalytic methodologies, multicomponent reactions, and metal-free synthetic strategies. The increasing use of recyclable catalysts, solvent-free protocols, microwave-assisted synthesis, ionic liquids, and visible-light-driven transformations clearly reflects the strong influence of green chemistry principles on modern quinoline synthesis. These approaches contribute not only to improved reaction efficiency and substrate scope but also to reduced waste generation, lower energy consumption, and enhanced operational simplicity.

An important aspect emerging from recent developments is the increasing mechanistic convergence observed across otherwise diverse quinoline synthetic methodologies. Despite differences in catalyst composition and activation modes, many contemporary approaches rely on recurring tandem condensation–cyclization–aromatization sequences, frequently coupled with hydrogen-transfer, Lewis-acid activation, radical annulation, or metal–ligand cooperative processes. This mechanistic convergence reflects a broader evolution toward tandem and cascade transformations capable of maximizing atom economy, minimizing waste generation, and improving overall process efficiency. Such trends illustrate how advances in catalyst design and mechanistic understanding are increasingly interconnected in the development of sustainable quinoline synthesis.

Despite these advances, several important challenges remain. Many catalytic systems still rely on elevated temperatures, long reaction times, specialized ligands, or relatively high catalyst loadings, which may limit industrial applicability and overall sustainability. In addition, the development of broadly applicable methodologies capable of simultaneously combining mild conditions, low catalyst loading, high selectivity, and broad functional-group tolerance remains an important objective. Further efforts are also needed to improve catalyst recyclability, reduce dependence on toxic or hazardous solvents, and expand the use of renewable feedstocks and biomass-derived substrates.

From a mechanistic perspective, future research will likely continue to benefit from a deeper understanding of hydrogen-transfer processes, metal–ligand cooperation, radical pathways, and photocatalytic activation mechanisms. The integration of computational chemistry, mechanistic studies, and rational catalyst design is expected to accelerate the discovery of next-generation catalytic systems with enhanced efficiency and selectivity. Moreover, the combination of photocatalysis, electrocatalysis, flow chemistry, and artificial intelligence-assisted reaction optimization may reveal new directions for sustainable quinoline synthesis.

Overall, the rapid evolution of catalytic and green synthetic methodologies demonstrates that quinoline chemistry remains a fertile platform for innovation in sustainable organic synthesis. Continued interdisciplinary efforts at the interface of catalysis, materials science, mechanistic chemistry, and green chemistry are expected to further expand the synthetic accessibility and practical utility of quinoline derivatives in both academic and industrial settings.

## Data Availability

Not applicable.

## Abbreviations

bcmim	1,3-bis(carboxymethyl)imidazolium
bmim	1-butyl-3-methylimidazolium
bpy	2,2′-bipyridyne
CSA	Camphorsulfonic acid
DFT	Density Functional Theory
DLS	Dynamic Light Scattering
DMF	Dimethyl formamide
DMS	Dimethyl sulfide
DMSO	Dimethyl sulfoxide
DMSO-d6	Hexadeuterodimethyl sulfoxide
DNA	Deoxyribonucleic acid
dsim	Imidazolium-1,3-disulfonic acid
FTIR	Fourier Transform Infrared Spectroscopy
IL	Ionic liquid
MCR	Multicomponent reactions
MsOH	Methanesulfonic acid
mw	Microwave
ppy	2-phenylpyridine
p-TSA	Para-toluenesulfonic acid
RNA	Ribonucleic acid
TEM	Transmission Electron Microscopy
TFA	Trifluoroacetic acid/trifluoroacetate
TfOH	Triflic acid
TGA	Thermogravimetric Analysis
XRD	X-ray Diffraction
